# Exploring the Causal Roles of Circulating Remnant Lipid Profile on Cardiovascular and Cerebrovascular Diseases: Mendelian Randomization Study

**DOI:** 10.2188/jea.JE20200305

**Published:** 2022-05-05

**Authors:** Shucheng Si, Lei Hou, Xiaolu Chen, Wenchao Li, Xinhui Liu, Congcong Liu, Yunxia Li, Tonghui Yuan, Jiqing Li, Bojie Wang, Hongkai Li, Fuzhong Xue

**Affiliations:** 1 Department of Biostatistics, School of Public Health, Cheeloo College of Medicine, Shandong University, Jinan, China; 2 Institute for Medical Dataology, Shandong University, Jinan, China; 3 National Institute of Health Data Science of China, Jinnan, China

**Keywords:** remnant lipids, coronary heart disease, ischemic stroke, Mendelian randomization

## Abstract

**Background:**

Causal evidence of circulating lipids especially the remnant cholesterol with cardiovascular and cerebrovascular disease (CVD) is lacking. This research aimed to explore the causal roles of extensive lipid traits especially the remnant lipids in CVD.

**Methods:**

Two-sample Mendelian randomization (TSMR) analysis was performed based on large-scale meta-analysis datasets in European ancestry. The causal effect of 15 circulating lipid profiles including 6 conventional lipids and 9 remnant lipids on coronary heart disease (CHD) and ischemic stroke (IS), as well as the subtypes, was assessed.

**Results:**

Apolipoprotein B (Apo B), total cholesterol (TC), low-density lipoprotein cholesterol (LDL-C), and triglyceride (TG) were still important risk factors for CHD and myocardial infarction (MI) but not for IS. Apo B is the strongest which increased the CHD and MI risk by 44% and 41%, respectively. The odds ratios (ORs) of total TG on CHD and MI were 1.25 (95% confidence interval [CI], 1.13–1.38) and 1.24 (95% CI, 1.11–1.38), respectively. A one standard deviation difference increased TG in medium very-low-density lipoproteins (M.VLDL.TG), TG in small VLDL (S.VLDL.TG), TG in very small VLDL (XS.VLDL.TG), TG in intermediate-density lipoproteins (IDL.TG), TG in very large HDL (XL.HDL.TG), and TG in small HDL (S.HDL.TG) particles also robustly increased the risk of CHD and MI by 9–28% and 9–27%, respectively. TG in very/extremely large VLDL (XXL.VLDL.TG and XL.VLDL.TG) were insignificant or even negatively associated with CHD (in multivariable TSMR), and negatively associated with IS as well.

**Conclusion:**

The remnant lipids presented heterogeneity and two-sided effects for the risk of CHD and IS that may partially rely on the particle size. The findings suggested that the remnant lipids were required to be intervened according to specific components. This research confirms the importance of remnant lipids and provides causal evidence for potential targets for intervention.

## INTRODUCTION

The plasma lipids, especially elevated low-density lipoprotein cholesterol (LDL-C), has been a well-known risk factor for cardiovascular and cerebrovascular disease.^1^ Wherefore, according to international guidelines, LDL-C has been recommended as a primary biomarker of cardiovascular and cerebrovascular disease and to guide lipid-lowering therapy.^2^^,^^3^ However, some increased risk of mortality was still reported among in-hospital patients with low LDL-C levels, which referred to as the “lipid paradox”.^4^^,^^5^ Moreover, a considerable residual risk remains after achieving the recommended LDL-C targets.^6^^,^^7^ Therefore, LDL-C and conventional lipid profiles could not explain all risks.

Studies showed that the above residual risk can be partly attributed to triglyceride-rich lipoproteins (TGLs), which include chylomicrons, very-low-density lipoproteins (VLDL), intermediate-density lipoproteins (IDL), and others.^8^ Cholesterol content of the TGLs is usually called remnant cholesterol.^9^ It has been noted that a third of plasma cholesterol was present in remnant lipoproteins and the large LDL and IDL were containing most of them.^10^ Since most research mainly focusing on the total cholesterol (TC), triglyceride (TG), LDL-C, and high-density lipoprotein cholesterol (HDL-C), remnant lipids have almost become “forgotten lipids”.^11^

Thus, the role of the circulating metabolic lipids especially the remnant lipids deserves further investigation. To date, causal evidence (‘causal’ in this article means the ‘statistical causality’) of associations between remnant lipids and risk of CVD and subtypes remains lacking. Considering the complexity of lipids and the inconsistent reports about cardiovascular and cerebrovascular disease,^5^^,^^12^^,^^13^ the causative effects of the extensive remnant lipid profiles on cardiovascular and cerebrovascular disease may be different. This may pose a challenge to the intervention of lipid-lowering therapy.

Mendelian randomization (MR) is a method for using genetic variants as proxies of risk factors to achieve robust causal inference of exposures on outcomes, which could avoid confounding and reverse causation bias effectively. In this work, we used the MR design to test the causal roles of circulating remnant lipid profile by using summary-level statistics of large-scale genome-wide association study (GWAS) including 15 lipid traits (6 conventional lipids and 9 remnant lipids) from Kettunen et al.^14^ This study aimed to provide an overview of the causal roles of plasma remnant lipids and exploring potential risk components to be intervened for coronary heart disease and ischemic stroke using two-sample Mendelian randomization (TSMR) analysis.

## METHODS

### Study population and variable ascertainment

Data sources used in this study are shown in Table 1. We retrieved summary-level data for the association between genetic instrumental variables (SNPs) and lipid profiles as well as the main cardiovascular and cerebrovascular disease. Genetic association with circulating lipid profiles was obtained from the available summary-level data published by Kettunen et al in the MR-Base platform (http://www.mrbase.org), which involved up to 24,925 individuals with European ancestry (details available in the original report).^14^ The exposures were further divided into group (a) and group (b), as shown in Table 1. Group (a) was 6 conventional lipids/lipoproteins including apolipoprotein A1 (Apo A1), apolipoprotein B (Apo B), TG, TC, LDL-C, and HDL-C. Group (b) included 9 main remnant lipids showed from large to small by particles as follows: triglycerides in largest VLDL (XXL.VLDL.TG), triglycerides in very large VLDL (XL.VLDL.TG), triglycerides in large VLDL (L.VLDL.TG), triglycerides in medium VLDL (M.VLDL.TG), triglycerides in small VLDL (S.VLDL.TG), triglycerides in very small VLDL (XS.VLDL.TG), triglycerides in IDL (IDL.TG), triglyceride in very large HDL (XL.HDL.TG), and triglycerides in small HDL (S.HDL.TG). In the original GWAS analysis, the effects of SNPs on these lipids (*β*x) were first adjusted for age, sex, fasting time (if applicable), and the top ten genetic principal components in 14 cohorts, and the resulting residuals were transformed into normal distribution by the inverse rank-based normal transformation.^14^ Each cohort was analyzed separately and combined in a fixed-effects meta-analysis. The effect size of each SNP on each exposure (*β*x) refers to per standard deviation (SD)-unit change of metabolites. SNPs in GWAS analysis were imputed with info >0.4 using 1,000 Genomes Project March 2012 version and the genomic positions referred to the human genome (build 39). For more details see the original GWAS study by Kettunen et al.^14^

**Table 1.** Data source and characteristics of included participants and phenotypes

Phenotypes	Mean^a^	Mean (SD)^b^	Consortium	Number of variants	Samplesize
**Exposures**					
**Group (a)**					
Apo A1, g/L	1.70	0.89 (0.32)	Kettunen et al	11,760,646	20,687
Apo B, g/L	0.98	0.81 (0.52)	Kettunen et al	11,813,266	20,690
TC, mmol/L	5.37	0.45 (0.39)	Kettunen et al	11,855,845	21,491
TG, mmol/L	1.18	1.05 (0.14)	Kettunen et al	11,871,391	21,545
LDL-C, mmol/L	2.11	19.00 (0.27)	Kettunen et al	11,871,461	21,559
HDL-C, mmol/L	1.69	2.62 (0.32)	Kettunen et al	11,865,530	21,555
**Group (b)**					
XXL.VLDL.TG, mmol/L	0.01	13.80 (0.53)	Kettunen et al	11,843,388	21,540
XL.VLDL.TG, mmol/L	0.03	9.00 (0.56)	Kettunen et al	11,814,232	21,548
L.VLDL.TG, mmol/L	0.12	0.98 (0.98)	Kettunen et al	11,753,671	21,239
M.VLDL.TG, mmol/L	0.24	0.52 (1.53)	Kettunen et al	11,766,530	21,241
S.VLDL.TG, mmol/L	0.23	5.16 (0.22)	Kettunen et al	11,859,725	21,558
XS.VLDL.TG, mmol/L	0.11	0.38 (0.51)	Kettunen et al	11,820,655	19,273
IDL.TG, mmol/L	0.11	NA	Kettunen et al	11,820,642	19,273
XL.HDL.TG, mmol/L	0.01	34.00 (0.14)	Kettunen et al	11,871,386	21,536
S.HDL.TG, mmol/L	0.04	10.10 (0.61)	Kettunen et al	11,871,440	21,558
**Outcome**					
Coronary heart disease	—	—	CARDIoGRAM plusC4D	9,455,779	184,305
Myocardial infarction	—	—	CARDIoGRAM plusC4D	9,289,492	171,875
Ischemic stroke	—	—	ISGC	2,421,920	29,633
Cardioembolic stroke	—	—	ISGC	2,421,920	21,185
Large vessel disease	—	—	ISGC	2,421,920	21,143
Small vessel disease	—	—	ISGC	2,421,920	20,675

Apo, apolipoprotein; HDL-C, high-density lipoprotein cholesterol; IDL, intermediate density lipoprotein; LDL-C, low-density lipoprotein cholesterol; SD, standard deviation; TC, total cholesterol; TG, triglycerides; VLDL, very low density lipoprotein; XXL.VLDL.TG, triglycerides in largest VLDL; XL.VLDL.TG, triglycerides in very large VLDL; L.VLDL.TG, triglycerides in large VLDL; M.VLDL.TG, triglycerides in medium VLDL; S.VLDL.TG, triglycerides in small VLDL; XS.VLDL.TG, triglycerides in very small VLDL; IDL.TG, triglycerides in IDL; XL.HDL.TG, triglycerides in very large HDL; S.HDL.TG, triglycerides in small HDL; NA, not available.

^a^Summary statistics of untransformed distributions from the largest cohort, Northern Finland Birth Cohort 1966 (NFBC 1966).

^b^Summary statistics of untransformed distributions from the cohort, Erasmus Rucphen Family Study (ERF).

Note: The unit of exposures (phenotypes column) showed in group (a) and group (b) were the unit used in the NFBC 1966 cohort corresponding to the second column. The values of all Mean/SD were recorded from the initial GWAS research reported by Kettunen et al (the supplement material of reference 14).

Summary-level data about outcomes were also selected from the European population as possible to control the potential bias of causality by racial heterogeneity. For cardiovascular diseases, coronary heart disease (CHD) and its subtype myocardial infarction (MI) were acquired from the Coronary ARtery DIsease Genome wide Replication and Meta-analysis (CARDIoGRAM) plus The Coronary Artery Disease (C4D) Genetics (CARDIoGRAMplusC4D) consortium (http://www.cardiogramplusc4d.org/) with 86,995 and 171,875 individuals, respectively.^15^^,^^16^ For cerebrovascular disease, ischemic stroke (IS), and three subtypes including cardioembolic stroke (CES), large vessel disease (LVD), and small vessel disease (SVD) were acquired from the International Stroke Genetics Consortium (ISGC) (https://strokegenetics.org/) with 29,633 participants.^17^

### Genetic correlation analysis

The cross-trait Linkage Disequilibrium Score Regression (LDSC) was a useful epidemiological tool to be utilized for estimating the genetic correlation of two traits.^18^ The whole GWAS dataset utilized in LDSC analysis for these lipid traits was downloaded from the official website http://www.computationalmedicine.fi/data#NMR_GWAS and the entire dataset for 6 outcomes was acquired from the website https://gwas.mrcieu.ac.uk/. In this analysis, linkage disequilibrium (LD) scores were calculated using the genetic dataset of European from the 1,000 Genomes Project as the reference panel. We further pruned the SNPs according to the recommended SNP list (named “w_hm3.noMHC.snplist” included about 1.2 million SNPs based on the HapMap 3 reference panel) in ‘ldsc’ software and the LD Hub website to improve computing performance (http://ldsc.broadinstitute.org) and restrict to a set of common variants. Each GWAS file was further matched with this common list and only retained the SNPs with the same alleles. Then, we adjusted the direction of effect values (*β*-coefficients) of each SNP across all traits to make sure they corresponded to consistent effect alleles (EA). These processes were performed by R software. In the LDSC analysis, we further excluded the SNPs with minor allele frequency (MAF) <0.01 by setting the parameter ‘- -maf-min 0.01’ in the ‘ldsc’ software. The LDSC analysis were performed based on the *β*-coefficients and *P* values of these SNPs. The genetic correlation coefficient was called *r*_g_ which ranged from −1 to 1.

### Genetic instrumental variables (IVs)

We utilized SNPs that were associated with above 15 circulating lipid profiles (group [a] plus group [b]) at the standard genome-wide significance threshold (*P* < 5 × 10^−8^). Then, we further adopted a stricter threshold (*P* < 2.27 × 10^−9^) that was set by Kettunen et al correcting for 22 independent tests in the initial GWAS dataset as one of the sensitivity analyses. Then we pruned these variants to avoid the correlation of SNPs according to the LD by setting the parameters in the ‘extract_instruments’ and ‘mv_extract_exposures’ functions of the ‘TwoSampleMR’ R package (parameter setting: *R*^2^ < 0.01, and <10,000 kb physical distances). For instrumental variables (IVs) within the LD threshold, the SNP has the lowest *P* values (usually called the top SNP) would be retained through above the functions. Since the study involves multi-exposure and multi-outcome, different instrumental SNPs for each pair of exposures and outcomes were required. We assessed the proportion of variance explained (*R*^2^ statistic) for each pair to avoid the weak instrumental bias. The *R*^2^ statistics were calculated by the formula 2*β*^2^ × *MAF* × (1 − *MAF*), where *β* was the effect of an SNP with the exposure and *MAF* is the minor allele frequency. Then, the strength of instrumentals was also evaluated by the *F* statistic using the formula *F* = [*R*^2^(*N* − 1 − *K*)]/[(1 − *R*^2^) × *K*], where *K* is the number of variants and *N* is the sample size. In general, the *F*-statistic >10 would be considered strong. We also mapped the gene information for these IV SNPs from the website: SNP and CNV Annotation Database http://www.scandb.org/newinterface/index_v1.html.

### Two-sample Mendelian randomization

Our research was based on the TSMR framework using the summarized associations (*β*-coefficients) of each instrumental SNP with exposures and outcomes. First, the association of genetic instruments and exposure (*β*_X_) and outcome (*β*_Y_) was obtained. We applied the inverse-variance weighted (IVW) method in the linear MR model. This conventional linear regression for each IV was weighted by inverse variance under a fixed-effect meta-analysis model.^19^ Besides, we also run the Weighted Median Estimator (WME) method^20^ simultaneously to assess the robustness of causal findings. This could consider as one of the sensitivity analysis when multiple genetic variants were used as instrumental variables.^21^ The WME method could produce robust estimates in the presence of some invalid genetic instruments (when the number of invalid IVs <50%). When the assumption of horizontal pleiotropy (the genetic instruments are associated with the outcome through other pathways beyond the exposure) is not met, we further applied a series of methods that could deal with such problems in different ways. First, the MR-Egger regression test for intercept was utilized to evaluate the existence of directional pleiotropy.^22^ Second, we performed the MR-PRESSO test to identify horizontal pleiotropic outliers in multi-instrument summary-level MR testing.^23^ Generally, the pleiotropy included balanced and unbalanced pleiotropy in theory. The balanced pleiotropy would not affect the MR causal estimators, while the unbalanced pleiotropic SNPs usually be identified as outliers and therefore affects the causal effect. So the MR-PRESSO method was utilized to deal with the above problems which could report the causal estimators after ruling out the potential horizontal pleiotropic SNPs. If there are any outliers, the results of MR-PRESSO would not equal the results from raw IVW MR and return the corrected values. In this research, we take the corrected causal effects of the MR-PRESSO method as our principal univariate results rather than the IVW method to avoid the influence of pleiotropic outliers. Furthermore, we applied the multivariable MR (MVMR) method to adjust other potential interactive lipid traits including total HDL-C and LDL-C. Finally, to clarify the influence of pleiotropic SNPs furthermore, the MR-TRYX (from the phrase ‘TReasure Your eXceptions’) method is applied under the TSMR framework as an in-depth sensitivity analysis. This method is designed to use outliers detected by the Radial MR method (generally regarded as an SNP with horizontal pleiotropy) in the original exposure-outcome analysis and re-estimate the original exposure-outcome association by removing or adjusting outlier SNPs for the horizontal pleiotropic pathways (see details in the reference).^24^ The potential horizontal pleiotropic pathways were searched from thousands of traits (here, we used 4,512 traits that the number of SNPs more than 1 million and sample size larger than 10,000) in the MR-Base platform. The MR-TRYX method would return causal estimators including raw (IVW result), outliers removed (remove all outliers), outliers removed (remove candidate outliers that detected pleiotropic pathways), and outliers adjusted results.

Finally, considering the number of tests in our research, the *P* values after Bonferroni correction was also reported at the threshold of 0.05/90 (90 represents the number of exposure-outcome pairs derived from the product of 15 lipid traits and 6 diseases). The significant result after Bonferroni correction (*P* < 0.05/90) was considered as a robust result. The consistent and significant results (*P* < 0.05) in at least two different methods (meant consistent results under different assumptions) could also be viewed as robust associations. *P*-value above the corrected significance threshold but <0.05 in at least one method was also considered as suggestive evidence. All analyses were two-tailed and performed using R software (Version 3.6.1; R Foundation for Statistical Computing, Vienna, Austria) with packages ‘TwoSampleMR’, ‘MRPRESSO’, ‘tryx’, and the ‘ldsc’ software.

## RESULTS

**Figure 1.**
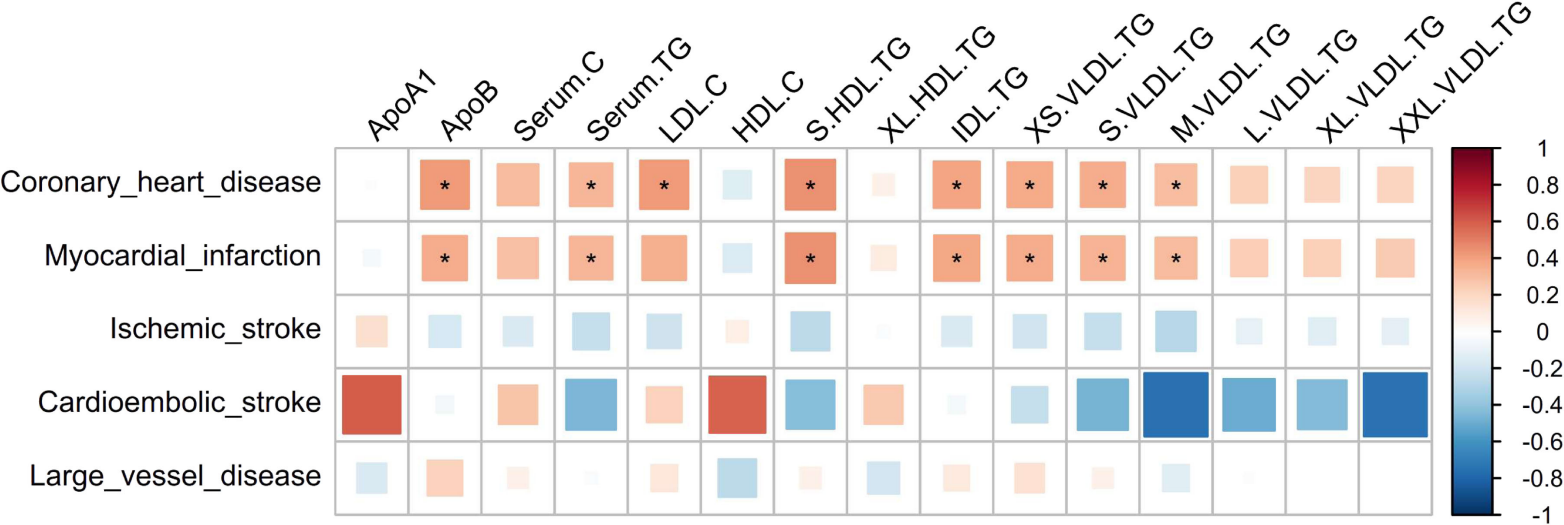
Genetic correlation between 15 lipid traits and 5 CVDs. ^*^: significant results after Bonferroni correction with *P* < 0.05/(5 × 15). The color represents the genetic correlation coefficient (r_g_), ranging from −1 (blue) to 1 (red). The area of the square represents the size of r_g_. In the genetic correlation analysis, small vessel disease was not included due to the small sample size and low heritability that could not perform effective analysis. The correlation coefficients greater than 1 in these results are limited to 1 in this heat map.

According to our criteria, a total of 144 SNPs was satisfying the assumption of the MR method and were used as instrumental variables for the 15 lipid profiles (eTable 1). For each pair of lipid and outcome, *F* statistics were ranged from 54.29 to 192.07, which considered as no weak instrumental bias. The detailed information about the instrumental SNPs sees eTable 2. The results of the lipid metabolism components are shown as follows:

Figure 1 showed the genetic correlation between 15 lipid traits and 5 cardiovascular and cerebrovascular diseases. Among these lipids, the Apo B, Serum.TG, S.HDL.TG, IDL.TG, XS.VLDL.TG, S.VLDL.TG, and M.VLDL.TG were significantly and positively associated with CHD and MI after Bonferroni correction. LDL.C is also significantly associated with CHD but not with MI. The lipid profiles showed no significant results with IS and its subtypes.

**Figure 2.**
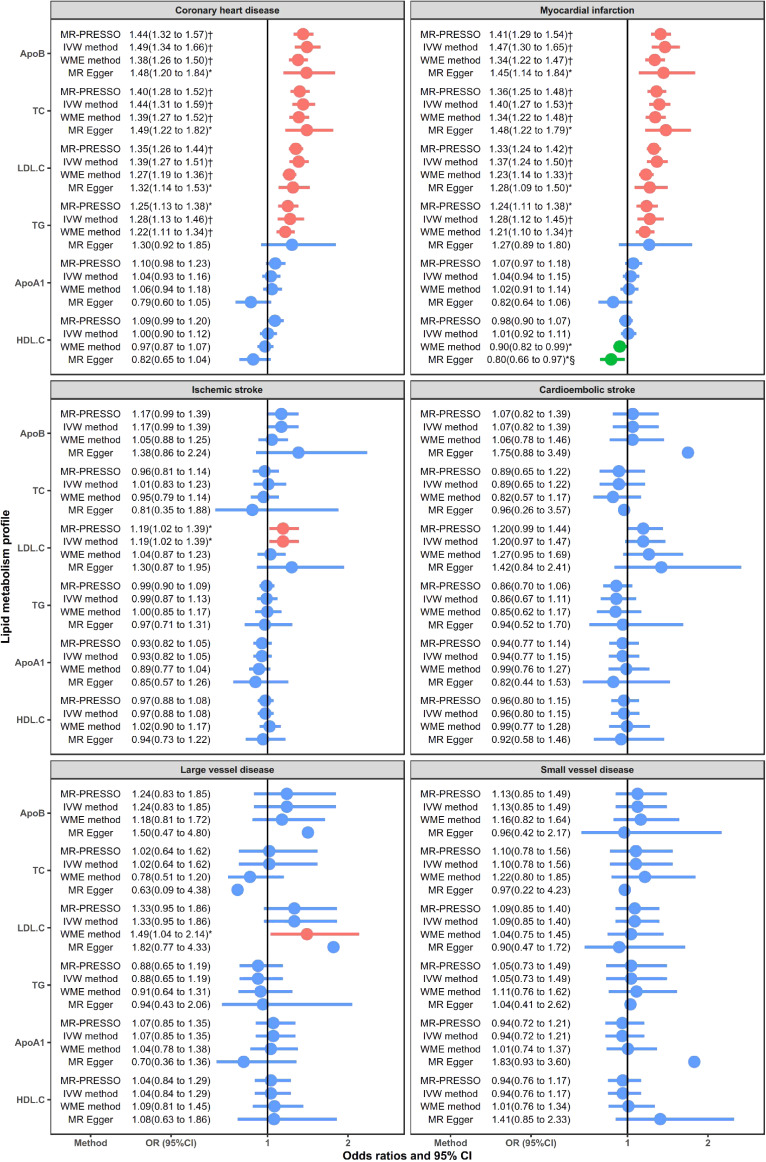
Causal relationship between main lipoprotein/lipids and CVDs. Red error bar: significantly positive association. Green error bar: significantly negative association. Blue error bar: insignificant association. ^*^P < 0.05; ^†^Significant result after Bonferroni correction; ^§^Results with potential horizontal pleiotropic tested using MR-Egger method.

Figure 2 showed the causal relationship between six conventional lipoprotein/lipids and cardiovascular and cerebrovascular diseases. As for the lipoprotein, Apo B presented the largest risk on CHD and its subtype MI. The OR by 1-SD increased Apo B was 1.44 (95% confidence interval [CI], 1.32–1.57) and 1.41 (95% CI, 1.29–1.54), respectively. In contrast, Apo A1 has no significant associations with any outcome. For lipid profiles, TC and LDL-C increased the risk of CHD by 40% (OR 1.40; 95% CI, 1.28–1.52) and 35% (OR 1.35; 95% CI, 1.26–1.44) and increased the risk of MI by 36% and 33%, respectively. In addition, TG also increased the risk of CHD by 25% (OR 1.25; 95% CI, 1.13–1.38) and MI by 24% (OR 1.24; 95% CI, 1.11–1.38). Such causal effects were also demonstrated in the sensitivity analyses by other MR methods or Bonferroni correction, so it could be viewed as a robust relationship. Besides, we found a positive association of LDL.C on IS (OR 1.19; 95% CI, 1.02–1.39) and LVD (OR 1.49; (95% CI, 1.04–2.14) (in WME method). Sensitivity analysis using stricter instrument variable thresholds repeated the above results (eFigure 1).

**Figure 3.**
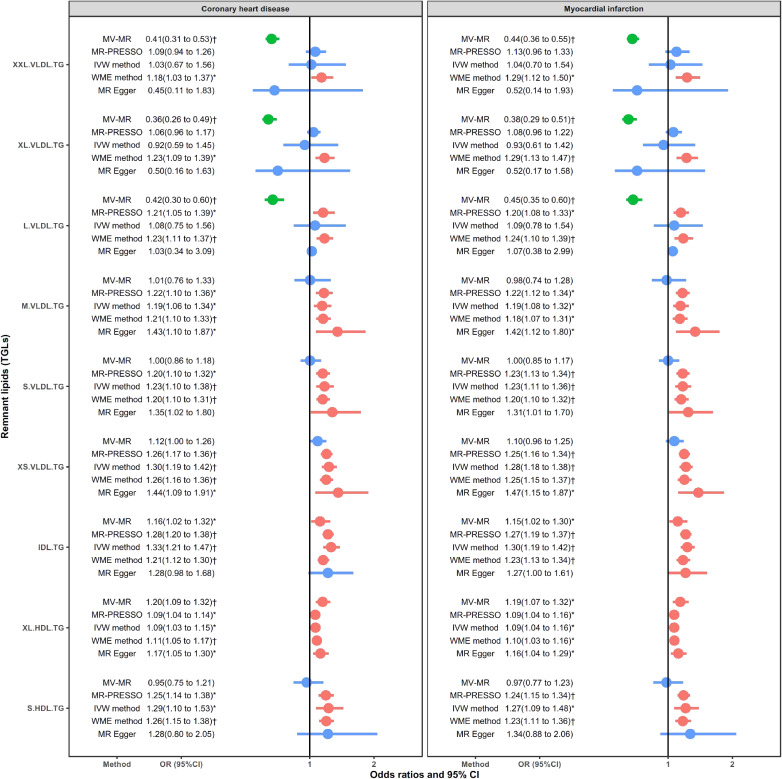
Causal relationship between circulating remnant lipids and CHD and MI. Red error bar: significantly positive association. Green error bar: significantly negative association. Blue error bar: insignificant association. ^*^P < 0.05; ^†^Significant result after Bonferroni correction; ^§^Results with potential horizontal pleiotropic tested using MR-Egger method.

Figure 3 showed 9 circulating remnant lipid profiles in relation to CHD and MI. The remnant lipids showed relatively consistent causal effects on CHD and MI. Overall, M.VLDL.TG, S.VLDL.TG, XS.VLDL.TG, IDL.TG, XL.HDL.TG, and S.HDL.TG presented a robustly positive association with CHD and MI (in all MR-PRESSO, IVW, and WME methods). For XXL.VLDL.TG and XL.VLDL.TG, only the WME method showed significant positive associations with CHD and MI. The L.VLDL.TG is also positively related to CHD and MI in both MR-PRESSO and WME methods. However, MVMR presented strong negative associations of the three largest lipid particles (XXL.VLDL.TG, XL.VLDL.TG, and L.VLDL.TG) when adjusted HDL-C and LDL-C. The ORs were 0.41 (95% CI, 0.31–0.53), 0.36 (95% CI, 0.26–0.49), and 0.42 (95% CI, 0.30–0.60) for CHD and 0.44 (95% CI, 0.36–0.55), 0.38 (95% CI, 0.29–0.51), and 0.45 (95% CI, 0.35–0.60) for MI, respectively. Overall, the remnant lipids showed consistently positive effects of TG in smaller-sized lipoprotein particles (including the IDL.TG and XL_HDL.TG) in both univariate and multivariate MR analysis, the significant positive effects of M.VLDL.TG, S.VLDL.TG, XS.VLDL.TG, and S.HDL.TG in univariate MR analysis, but protective effects of TG in larger-sized lipoprotein like the XXL.VLDL.TG and XL.VLDL.TG in multivariate MR analysis. The above analysis confirmed that remnant lipids are independent factors (both positive and negative) for cardiovascular disease. The results were consistent in the sensitivity analysis using stricter instrument variable thresholds (eFigure 2). MR-Egger regression test for intercept showed no horizontal pleiotropy. The results of MR are generally consistent with genetic correlations.

**Figure 4.**
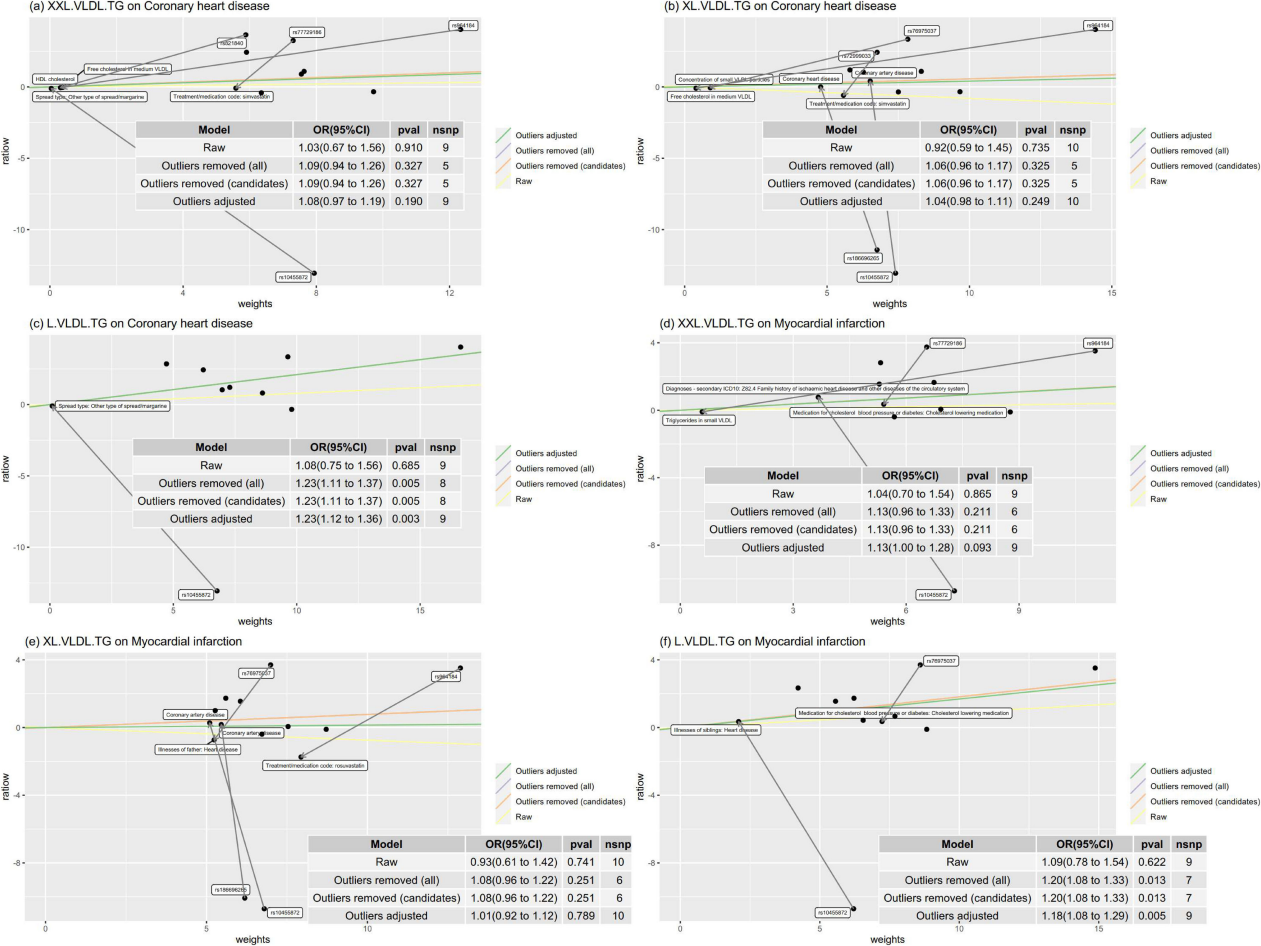
In-depth analysis of XXL.VLDL, XL.VLDL, and L.VLDL.TG on CHD and MI adjusting the SNP effects on the candidate traits using the MR-TRYX method. The x-axis represents the weights of each SNP contributes to the causal estimators, and the y-axis represents the product of the causal effect and weights. The slopes represent causal estimators in different models (different lines). Text annotations indicate the identified outlier SNPs and related pleiotropic traits.

**Figure 5.**
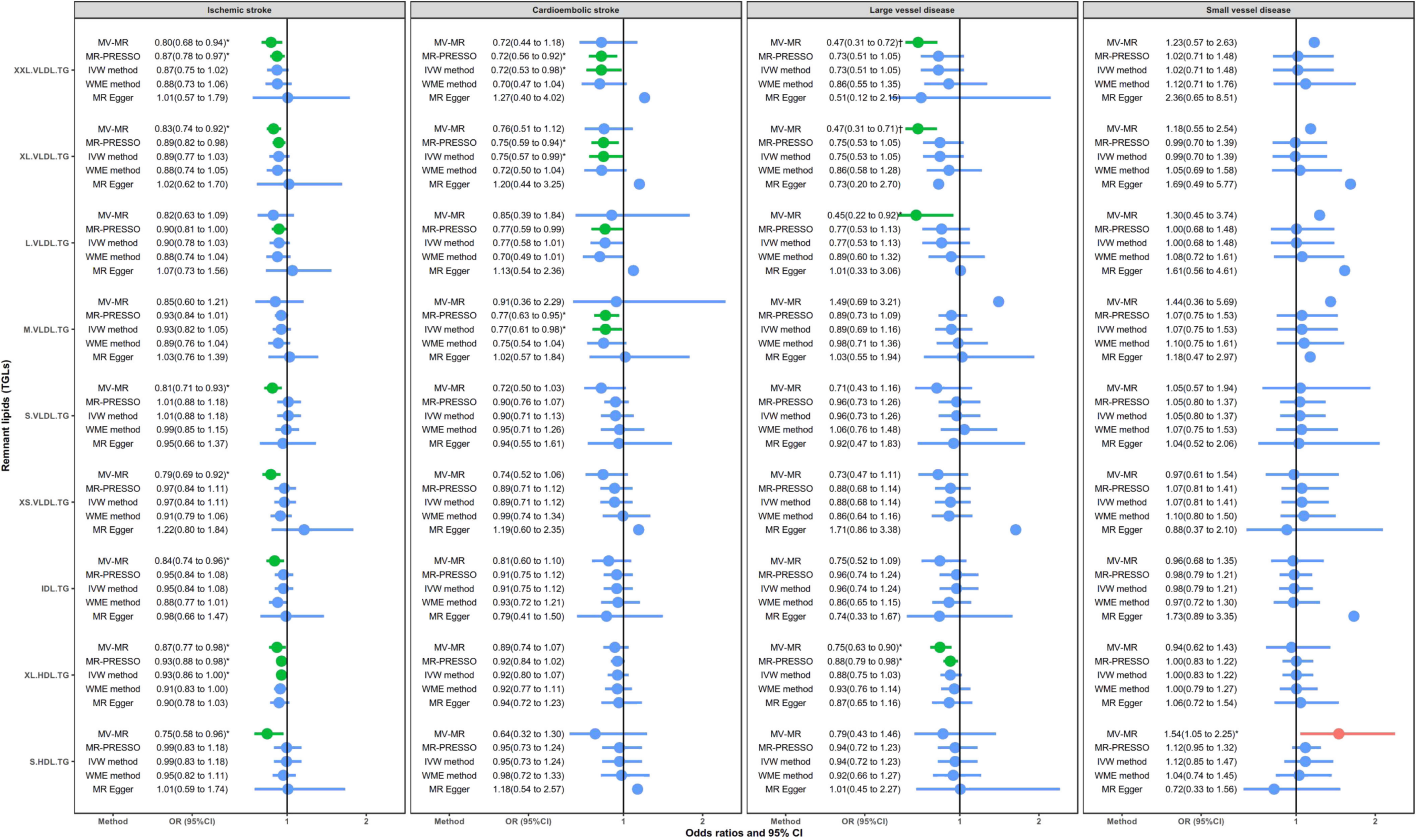
Causal relationship between circulating remnant lipids and IS and subtypes. Red error bar: significantly positive association. Green error bar: significantly negative association. Blue error bar: insignificant association. ^*^P < 0.05; ^†^Significant result after Bonferroni correction; ^§^Results with potential horizontal pleiotropic tested using the MR-Egger method.

Figure 4 showed an in-depth analysis of the concerning results for XXL.VLDL.TG, XL.VLDL.TG, and L.VLDL.TG for CHD and MI using the MR-TRYX method. Among these results, both XXL.VLDL.TG and XL.VLDL.TG has identifiable outliers and insignificantly associated with CHD and MI in any outliers removed or outliers adjusted results. However, the smaller particle L.VLDL.TG was positively associated with CHD and MI when removed or adjusted the outliers. The adjusted ORs of XXL.VLDL.TG, XL.VLDL.TG, and L.VLDL.TG were 1.08 (95% CI, 0.97–1.19), 1.04 (95% CI, 0.98–1.11), and 1.23 (95% CI, 1.12–1.36) for CHD and 1.13 (95% CI, 1.00–1.28), 1.01 (95% CI, 0.92–1.12), and 1.18 (95% CI, 1.08–1.29) for MI, respectively. The MR-TRYX analysis for all lipid traits is presented in eTable 3. A total of 34 exposure-outcome pairs were identified with pleiotropic outliers that could perform the MR-TRYX analysis. After adjusting for any possible pleiotropy or removing the outlier SNPs, the vast majority showed consistency as the raw IVW method. Except for the XXL.VLDL.TG, XL.VLDL.TG, and L.VLDL.TG mentioned above, only Apo A1 presented inconsistent results for CHD and MI and showed significant positive effects. The effect of IDL.TG and XS.VLDL.TG on IS was also inconsistent with the raw IVW results but only slightly changed and consistent with the multivariate MR analysis in Figure 5.

Figure 5 shows circulating remnant lipid profiles in relation to IS and subtypes. Different from the CHD and MI, the remnant lipids showed a wide range of insignificant or protective roles. Except for M.VLDL.TG, all lipids were negatively in relation to IS in at least one method, especially the MVMR. MVMR also showed a negative association of XXL.VLDL.TG, XL.VLDL.TG, L.VLDL.TG, and XL.HDL.TG with LVD. MR-PRESSO or IVW method also presented negative associations for XXL.VLDL.TG, XL.VLDL.TG, L.VLDL.TG, and M.VLDL.TG on CES. Besides, S.HDL.TG significantly increased the risk of SVD in MVMR analysis. MR-Egger regression test for intercept showed no horizontal pleiotropy. Results in the sensitivity analysis using stricter instrument variable thresholds further support these findings (eFigure 3).

Overall, the causal role of remnant lipids is heterogeneous in cardiovascular disease and cerebrovascular disease. Cardiovascular metabolic outcomes are more sensitive to the remnant lipids. The role of remnant lipids also showed a heterogeneous that depends on the size of lipoproteins. TG in particles like XL.VLDL and XXL.VLDL will not significantly increase or even decrease the risk of CVD. TG in particles that smaller than M.VLDL will generate a robustly positive association with CHD and MI, but less significantly associated with the IS and its subtypes.

## DISCUSSION

Our findings from this TSMR study suggest that Apo B, TC, LDL.C, and TG were important risk factors for CHD and MI. Importantly, the effects of “remnant lipids” should not be ignored. The hazard of remnant lipids was prone to depend on the particle size, since the TG in very/extremely large VLDL (XL.VLDL/XXL.VLDL) may not be a harmful component considering the insignificant results in univariate MR analysis, the protective effect in multivariate MR analysis for CHD, and the protective role in IS. The evidence suggests that circulating lipids are heterogeneous for cardiovascular and cerebrovascular diseases depending on the remnant lipids subclass. The above findings added new information to the understanding of the remnant lipids in causality and focus attention toward other lipoproteins targets beyond LDL-C in cardiovascular and cerebrovascular prevention.

For CHD and MI, the high risk of LDL-C, total TG, and TG in VLDL and IDL (not XXL.VLDL, XL.VLDL, and L.VLDL) was consistent with related studies^1^^,^^8^^,^^11^^,^^25^^–^^27^ and MR study have also reported that 1-mmol/L higher genetically determined LDL-C was associated with a 50% higher risk of CHD.^27^ Our research further confirms the causal effects of triglyceride-rich lipoproteins, not just the total TG and LDL-C. Evidence from this study partly explains the residual risk of LDL-C, as some TG in VLDL appear to have a similar role compared with LDL-C. From the biological mechanism, the above results seem to be reasonable. Previous experimental studies have shown that the TGLs, VLDL, and IDL can enter the arterial intima and become trapped,^10^^,^^28^^–^^30^ while large chylomicrons and the largest VLDL particles (eg, XXL.VLDL and XL.VLDL) cannot enter the arterial intima due to their size.^31^^–^^33^ Therefore, TG in smaller-sized lipoprotein particles exhibit more chance of inducing an atherogenic response in the arterial intima. By combining with proteoglycans and other components of the arterial intima, these particles get trapped and difficult to return to the arterial lumen, causing accumulation of cholesterol.^30^ After being trapped, they would be modified before being taken up by scavenger receptors on the surface of macrophages, leading to the formation of foam cells and the development of atherosclerotic plaque.^11^ Moreover, TGLs could also produce lipolytic products, such as oxidized FFAs, which induce the production of cytokines, interleukins, and proatherogenic adhesion molecules that may generate local inflammation in the arterial wall.^34^ So the major atherogenic effect of high TG-rich lipoprotein is largely due to its particle size, which leads to a prolonged arterial intima residence time, increased penetration into the arterial wall, and increased susceptibility to oxidation. Thus, our study showed the aggregation of the atherogenic effect of lipoprotein, in other words, the magnitude of CHD risk is prone to depend on the number of lipoprotein particles that could enter arterial intima and been trapped.

For IS and three subtypes, our research showed a weaker even protective effect of lipids on IS than CHD. In fact, only LDL-C was positively associated with IS and LVD subtypes. Such results seem to be very common. A study from METASTROKE has mentioned that genetic variants that confer lifelong LDL-C differences show a weaker effect on IS than on CHD.^27^ Another study also reported that LDL-C lowering is likely to prevent LVD but may not prevent SVD nor CS,^35^ which is consistent with our findings. The heterogeneity with CHD and IS has also been pointed out by previous GWAS studies, which indicated enrichment of lipid pathways in CAD but not in ischemic stroke pathogenesis.^17^^,^^36^ In mechanism, LVD is related to the atherothrombotic lesions within large extracranial or intracranial arteries, while SVD is caused by lipohyalinosis of the small penetrating arteries within the brain,^37^ so hazard of lipids should be more evident in CHD and LVD than in other diseases. In addition to the above findings, we also found the protective effect of TG in XXL.VLDL, XL.VLDL, and L.VLVL on IS and subtypes. An important reason may be that these extremely large particles have trouble entering the vessel wall,^31^^–^^33^ thereby avoiding the risk of atherosclerosis. Moreover, due to the large size, the above lipoproteins could carry more lipids than LDL-C,^38^ so it shares and decreases the transport of LDL-C, resulting in a lower risk of disease.

The above findings provide new evidence for the clinical practice of cardiovascular and cerebrovascular disease prevention. Previous research has proved the key role of LDL-C and Apo B, which was set as the primary target goal for anti-atherogenic therapy in the clinical guideline in different countries.^2^^,^^39^ Our research further confirmed that TG-rich remnant lipids may be independent risk factors for cardiovascular and cerebrovascular diseases after adjusted for LDL-C and HDL-C. This suggests that remnant lipids may become a new target for the prevention of cardiovascular and cerebrovascular diseases. However, our analysis also showed the causal effect of remnant lipids is more likely to depend on the particle size of its lipoproteins (larger particles were insignificant or even protective for cardiovascular and cerebrovascular disease). Therefore, clinical interventions for remnant lipids should follow the perspective of precision medicine. Since very few clinical trials show additional effects by TG lowering therapy under statin treatment, future lipid-lowering measures may require targeted drugs based on specific lipoproteins, especially the smaller lipoprotein particles.

Overall, our research has some advantages. First, we performed a MR study that covered 6 conventional lipids and 9 circulating remnant lipid profiles. This has considerable value in identifying potential biomarkers and drug targets. Second, we found the heterogeneity of lipids on CHD and IS by particle size. This would help researchers to further discover or confirm the underlying pathogenesis. Third, this study showed the causal effects of the remnant lipids and proved that not all remnant lipids will be harmful. Of course, our research also has some limitations. Though the MR method could rule out confounding, it has trouble in dealing with horizontal pleiotropic effects, especially the common gene regulation mechanism across lipids. However, perform a study on detailed phenotypes, such as lipoprotein subclass profiling, has been advised in addressing the validity of the genetic instruments used in MR.^40^ Besides, we also took some measures such as MR-Egger, MR-PRESSO, MVMR, and MR-TRYX to test and correct some potential pleiotropic. Despite these limitations, our research still provides an overview of relevant fields, and most of the results could be explained by biological mechanisms. More causal and experimental research needs to further confirm our findings.

In conclusion, the remnant lipid profiles have heterogeneity for the risk of CHD and IS by the particle size of triglyceride-rich lipoproteins. The remnant lipids presented both positive and negative effects for cardiovascular and cerebrovascular disease that suggest not all remnant lipids was required intervention. This research provides causal evidence for potential targets of remnant lipids. More research and design are needed to further confirm its effectiveness.

## APPENDIX A. SUPPLEMENTARY DATA

eTable 1. Detailed information about instrumental SNPs for each pair of exposure-outcome.

eTable 2. Summary information of instrumental variables in TSMR analysis.

eTable 3. In-depth analysis by the MR-TRYX method.

eFigure 1. Causal relationship between main lipoprotein/lipids and CVDs in sensitivity analysis.

eFigure 2. Causal relationship between circulating remnant lipids and CHD and MI in sensitivity analysis.

eFigure 3. Causal relationship between circulating remnant lipids and IS and subtypes in sensitivity analysis.
