# Supplementary material for: Exploring the Causal Roles of Circulating Remnant Lipid Profile on Cardiovascular and Cerebrovascular Diseases: Mendelian Randomization Study
Source: J Epidemiol. 2022 May 5;32(5):205–14. doi: 10.2188/jea.JE20200305 (PMC8979919; doi:10.2188/jea.JE20200305)
Supplement: Supplementary file 1 [file je-32-205-s001.pdf]

Exploring the causal roles of circulating remnant lipid profile on cardiovascular and cerebrovascular diseases: Mendelian randomization study

Running title: Causal effect of remnant lipid profiles on CVD

Authors: Shucheng Si[1], PhD, Lei Hou[1], PhD, Xiaolu Chen[1], MSc, Wenchao Li[1], MSc, Xinhui Liu[1], MSc, Congcong Liu[1], MSc, Yunxia Li[1], MSc, Tonghui Yuan[1], MSc, Jiqing Li[1], PhD, Bojie Wang[1], MSc, Hongkai Li[1,2], PhD, Fuzhong Xue[1,2,3], PhD

1. Department of Biostatistics, School of Public Health, Cheeloo College of Medicine, Shandong University, Jinan, 250012, China.

2. Institute for Medical Dataology, Shandong University, Jinan, 250002, China.

3. National Institute of Health Data Science of China, China.

Correspondence to: Fuzhong Xue, PhD, Department of Biostatistics, School of Public Health, Cheeloo College of Medicine, Shandong University, No.44 Wenhuaxi Road, Jinan, 250012, China. (Email: xuefzh@sdu.edu.cn; Tel: (+86)-531-88380280; Fax: (+86)-531-88380280).

Conflict of interest: The authors do not have any conflict of interest.

eTable 1. Detailed information about instrumental SNPs for each pair of exposure-outcome.

No.	SNP	chr	pos	exposure	outcome	beta.x	se.x	beta.y	se.y	gene	left_gene	right_gene
1	rs144064722	4	73406173	ApoA1	Coronary heart disease	0.203723	0.03504	0.028313	0.031588			
2	rs144064722	4	73406173	ApoA1	Myocardial infarction	0.203723	0.03504	0.032534	0.034056			
3	rs1461729	8	9187242	ApoA1	Coronary heart disease	0.086363	0.01519	0.010384	0.015457		PPP1R3B	LOC100129150
4	rs1461729	8	9187242	ApoA1	Ischemic stroke	0.086363	0.01519	-0.0261	0.0274		PPP1R3B	LOC100129150
5	rs1461729	8	9187242	ApoA1	Myocardial infarction	0.086363	0.01519	0.00022829	0.017174		PPP1R3B	LOC100129150
6	rs1461729	8	9187242	ApoA1	Cardioembolic stroke	0.086363	0.01519	-0.0919	0.0537		PPP1R3B	LOC100129150
7	rs1461729	8	9187242	ApoA1	Large vessel disease	0.086363	0.01519	-0.0264	0.0594		PPP1R3B	LOC100129150
8	rs1461729	8	9187242	ApoA1	Small vessel disease	0.086363	0.01519	-0.0119	0.0603		PPP1R3B	LOC100129150
9	rs174594	11	61623140	ApoA1	Large vessel disease	0.071714	0.0105	0.0832	0.0401	FADS2	LOC100131326	FADS3
10	rs174594	11	61619829	ApoA1	Myocardial infarction	0.071714	0.0105	0.0196856	0.0115	FADS2	LOC100131326	FADS3
11	rs174594	11	61623140	ApoA1	Cardioembolic stroke	0.071714	0.0105	0.0366	0.0374	FADS2	LOC100131326	FADS3
12	rs174594	11	61623140	ApoA1	Ischemic stroke	0.071714	0.0105	0.0452	0.0185	FADS2	LOC100131326	FADS3
13	rs174594	11	61623140	ApoA1	Small vessel disease	0.071714	0.0105	-0.0368	0.041	FADS2	LOC100131326	FADS3
14	rs174594	11	61619829	ApoA1	Coronary heart disease	0.071714	0.0105	0.016821	0.010328	FADS2	LOC100131326	FADS3
15	rs1883025	9	107664301	ApoA1	Cardioembolic stroke	-0.07984	0.01332	-0.0129	0.0368	ABCA1	NIPSNAP3B	SLC44A1
16	rs1883025	9	107664301	ApoA1	Large vessel disease	-0.07984	0.01332	-0.0302	0.04	ABCA1	NIPSNAP3B	SLC44A1
17	rs1883025	9	107664301	ApoA1	Ischemic stroke	-0.07984	0.01332	0.021	0.0189	ABCA1	NIPSNAP3B	SLC44A1
18	rs1883025	9	107664301	ApoA1	Coronary heart disease	-0.07984	0.01332	-0.025887	0.010457	ABCA1	NIPSNAP3B	SLC44A1
19	rs1883025	9	107664301	ApoA1	Small vessel disease	-0.07984	0.01332	0.1045	0.0409	ABCA1	NIPSNAP3B	SLC44A1
20	rs1883025	9	107664301	ApoA1	Myocardial infarction	-0.07984	0.01332	-0.0258134	0.011584	ABCA1	NIPSNAP3B	SLC44A1
21	rs247617	16	56993324	ApoA1	Cardioembolic stroke	0.197243	0.01169	0.0074	0.0345		HERPUD1	CETP
22	rs247617	16	56993324	ApoA1	Small vessel disease	0.197243	0.01169	0.0027	0.0382		HERPUD1	CETP
23	rs247617	16	56993324	ApoA1	Ischemic stroke	0.197243	0.01169	0.0102	0.0175		HERPUD1	CETP
24	rs247617	16	56990716	ApoA1	Myocardial infarction	0.197243	0.01169	-0.0261377	0.011415		HERPUD1	CETP
25	rs247617	16	56990716	ApoA1	Coronary heart disease	0.197243	0.01169	-0.030884	0.010212		HERPUD1	CETP
26	rs247617	16	56993324	ApoA1	Large vessel disease	0.197243	0.01169	0.0256	0.0369		HERPUD1	CETP
27	rs261291	15	58683366	ApoA1	Ischemic stroke	0.144314	0.01091	-0.0215	0.0161		LOC441726	LIPC
28	rs261291	15	58680178	ApoA1	Myocardial infarction	0.144314	0.01091	0.00516156	0.010716		LOC441726	LIPC
29	rs261291	15	58683366	ApoA1	Small vessel disease	0.144314	0.01091	-0.0405	0.0359		LOC441726	LIPC
30	rs261291	15	58683366	ApoA1	Large vessel disease	0.144314	0.01091	-0.016	0.0343		LOC441726	LIPC
31	rs261291	15	58680178	ApoA1	Coronary heart disease	0.144314	0.01091	0.012987	0.009619		LOC441726	LIPC
32	rs261291	15	58683366	ApoA1	Cardioembolic stroke	0.144314	0.01091	-0.0091	0.0317		LOC441726	LIPC
33	rs261334	15	58726744	ApoA1	Ischemic stroke	-0.15655	0.01256	0.0383	0.0211	LIPC	LOC441726	ADAM10
34	rs261334	15	58726744	ApoA1	Cardioembolic stroke	-0.15655	0.01256	0.0623	0.0423	LIPC	LOC441726	ADAM10
35	rs261334	15	58726744	ApoA1	Coronary heart disease	-0.15655	0.01256	-0.03756	0.011043	LIPC	LOC441726	ADAM10
36	rs261334	15	58726744	ApoA1	Small vessel disease	-0.15655	0.01256	-0.0739	0.0451	LIPC	LOC441726	ADAM10
37	rs261334	15	58726744	ApoA1	Myocardial infarction	-0.15655	0.01256	-0.0402744	0.012395	LIPC	LOC441726	ADAM10
38	rs261334	15	58726744	ApoA1	Large vessel disease	-0.15655	0.01256	0.0677	0.0452	LIPC	LOC441726	ADAM10
39	rs4860951	4	69375736	ApoA1	Coronary heart disease	0.073728	0.01322	0.040654	0.016657		TMPRSS11E	UGT2B29P

40	rs4860951	4	69375736	ApoA1	Myocardial infarction	0.073728	0.01322	0.0228272	0.017995		TMPRSS11E	UGT2B29P
41	rs4939873	18	47062054	ApoA1	Large vessel disease	0.13435	0.02395	-0.0047	0.1188		LOC647073	LIPG
42	rs4939873	18	47062054	ApoA1	Cardioembolic stroke	0.13435	0.02395	0.1185	0.0976		LOC647073	LIPG
43	rs4939873	18	47062054	ApoA1	Small vessel disease	0.13435	0.02395	0.0847	0.1432		LOC647073	LIPG
44	rs4939873	18	47062054	ApoA1	Coronary heart disease	0.13435	0.02395	0.012152	0.03531		LOC647073	LIPG
45	rs4939873	18	47062054	ApoA1	Myocardial infarction	0.13435	0.02395	0.0301786	0.03942		LOC647073	LIPG
46	rs4939873	18	47062054	ApoA1	Ischemic stroke	0.13435	0.02395	-0.0123	0.0573		LOC647073	LIPG
47	rs5880	16	57015091	ApoA1	Coronary heart disease	-0.18686	0.03198	0.007277	0.022421	CETP	HERPUD1	LOC100130044
48	rs5880	16	57015091	ApoA1	Myocardial infarction	-0.18686	0.03198	0.0247342	0.0245	CETP	HERPUD1	LOC100130044
49	rs5880	16	57015091	ApoA1	Large vessel disease	-0.18686	0.03198	-0.0992	0.0884	CETP	HERPUD1	LOC100130044
50	rs5880	16	57015091	ApoA1	Ischemic stroke	-0.18686	0.03198	0.0364	0.0422	CETP	HERPUD1	LOC100130044
51	rs5880	16	57015091	ApoA1	Cardioembolic stroke	-0.18686	0.03198	0.0874	0.0838	CETP	HERPUD1	LOC100130044
52	rs5880	16	57015091	ApoA1	Small vessel disease	-0.18686	0.03198	-0.0283	0.0961	CETP	HERPUD1	LOC100130044
53	rs6494025	15	58876786	ApoA1	Ischemic stroke	0.184905	0.02993	-0.047	0.048		LIPC	ADAM10
54	rs6494025	15	58876786	ApoA1	Large vessel disease	0.184905	0.02993	-0.0016	0.0975		LIPC	ADAM10
55	rs6494025	15	58876786	ApoA1	Coronary heart disease	0.184905	0.02993	0.043578	0.02554		LIPC	ADAM10
56	rs6494025	15	58876786	ApoA1	Myocardial infarction	0.184905	0.02993	0.0413404	0.029307		LIPC	ADAM10
57	rs6494025	15	58876786	ApoA1	Small vessel disease	0.184905	0.02993	-0.0599	0.1171		LIPC	ADAM10
58	rs6494025	15	58876786	ApoA1	Cardioembolic stroke	0.184905	0.02993	-0.0048	0.0842		LIPC	ADAM10
59	rs6507939	18	47176261	ApoA1	Coronary heart disease	0.108424	0.01427	-0.024621	0.013496		LIPG	LOC100129143
60	rs6507939	18	47176261	ApoA1	Ischemic stroke	0.108424	0.01427	-0.0068	0.0237		LIPG	LOC100129143
61	rs6507939	18	47176261	ApoA1	Large vessel disease	0.108424	0.01427	0.0528	0.0514		LIPG	LOC100129143
62	rs6507939	18	47176261	ApoA1	Myocardial infarction	0.108424	0.01427	-0.0174663	0.015114		LIPG	LOC100129143
63	rs6507939	18	47176261	ApoA1	Cardioembolic stroke	0.108424	0.01427	0.0079	0.0479		LIPG	LOC100129143
64	rs6507939	18	47176261	ApoA1	Small vessel disease	0.108424	0.01427	-0.0005	0.0522		LIPG	LOC100129143
65	rs75835816	8	19885513	ApoA1	Myocardial infarction	-0.22095	0.0388	0.0295779	0.050057			
66	rs75835816	8	19885513	ApoA1	Coronary heart disease	-0.22095	0.0388	0.066618	0.043542			
67	rs10056811	5	74605220	ApoB	Myocardial infarction	0.085783	0.01057	0.0221538	0.010655		ANKRD31	LOC728775
68	rs10056811	5	74605220	ApoB	Cardioembolic stroke	0.085783	0.01057	-0.023	0.0348		ANKRD31	LOC728775
69	rs10056811	5	74605220	ApoB	Coronary heart disease	0.085783	0.01057	0.025525	0.009749		ANKRD31	LOC728775
70	rs10056811	5	74605220	ApoB	Large vessel disease	0.085783	0.01057	-0.0701	0.038		ANKRD31	LOC728775
71	rs10056811	5	74605220	ApoB	Small vessel disease	0.085783	0.01057	0.0149	0.0382		ANKRD31	LOC728775
72	rs10056811	5	74605220	ApoB	Ischemic stroke	0.085783	0.01057	-0.0082	0.0176		ANKRD31	LOC728775
73	rs1081105	19	45412955	ApoB	Coronary heart disease	0.222868	0.03928	0.086187	0.041158	APOE	APOE	APOC1
74	rs1081105	19	45412955	ApoB	Myocardial infarction	0.222868	0.03928	0.142826	0.048309	APOE	APOE	APOC1
75	rs115849089	8	19912370	ApoB	Myocardial infarction	-0.09957	0.01713	-0.0667741	0.017021			
76	rs115849089	8	19912370	ApoB	Coronary heart disease	-0.09957	0.01713	-0.057989	0.015294			
77	rs11591147	1	55505647	ApoB	Myocardial infarction	-0.43794	0.0353	-0.354653	0.068555	PCSK9	BSND	USP24
78	rs11591147	1	55505647	ApoB	Coronary heart disease	-0.43794	0.0353	-0.256502	0.057259	PCSK9	BSND	USP24
79	rs1168041	1	62960250	ApoB	Small vessel disease	0.070994	0.01146	-0.0781	0.0389	DOCK7	USP1	ANGPTL3
80	rs1168041	1	62960250	ApoB	Large vessel disease	0.070994	0.01146	-0.0152	0.0373	DOCK7	USP1	ANGPTL3

81	rs1168041	1	62960250	ApoB	Coronary heart disease	0.070994	0.01146	0.011014	0.01011	DOCK7	USP1	ANGPTL3
82	rs1168041	1	62960250	ApoB	Ischemic stroke	0.070994	0.01146	-0.0095	0.0178	DOCK7	USP1	ANGPTL3
83	rs1168041	1	62960250	ApoB	Cardioembolic stroke	0.070994	0.01146	-0.0236	0.0353	DOCK7	USP1	ANGPTL3
84	rs1168041	1	62960250	ApoB	Myocardial infarction	0.070994	0.01146	0.00067632	0.011148	DOCK7	USP1	ANGPTL3
85	rs1260326	2	27730940	ApoB	Myocardial infarction	-0.06679	0.01039	0.00111651	0.010666	GCKR	FNDC4	LOC100130981
86	rs1260326	2	27730940	ApoB	Large vessel disease	-0.06679	0.01039	0.0392	0.0341	GCKR	FNDC4	LOC100130981
87	rs1260326	2	27730940	ApoB	Cardioembolic stroke	-0.06679	0.01039	0.0401	0.0314	GCKR	FNDC4	LOC100130981
88	rs1260326	2	27730940	ApoB	Coronary heart disease	-0.06679	0.01039	0.003257	0.00962	GCKR	FNDC4	LOC100130981
89	rs1260326	2	27730940	ApoB	Ischemic stroke	-0.06679	0.01039	0.012	0.0162	GCKR	FNDC4	LOC100130981
90	rs1260326	2	27730940	ApoB	Small vessel disease	-0.06679	0.01039	-0.0441	0.0353	GCKR	FNDC4	LOC100130981
91	rs1367117	2	21263900	ApoB	Large vessel disease	0.108852	0.01119	-0.0405	0.0398	APOB	C2orf43	LOC100129278
92	rs1367117	2	21263900	ApoB	Ischemic stroke	0.108852	0.01119	0.0067	0.0188	APOB	C2orf43	LOC100129278
93	rs1367117	2	21263900	ApoB	Cardioembolic stroke	0.108852	0.01119	-0.0051	0.0375	APOB	C2orf43	LOC100129278
94	rs1367117	2	21263900	ApoB	Myocardial infarction	0.108852	0.01119	0.0382631	0.01173	APOB	C2orf43	LOC100129278
95	rs1367117	2	21263900	ApoB	Small vessel disease	0.108852	0.01119	0.0213	0.0414	APOB	C2orf43	LOC100129278
96	rs1367117	2	21263900	ApoB	Coronary heart disease	0.108852	0.01119	0.041139	0.010635	APOB	C2orf43	LOC100129278
97	rs137992968	19	11239696	ApoB	Myocardial infarction	-0.19469	0.03466	-0.0815463	0.039958			
98	rs137992968	19	11239696	ApoB	Coronary heart disease	-0.19469	0.03466	-0.071649	0.036181			
99	rs142130958	19	11202306	ApoB	Large vessel disease	-0.19965	0.01674	-0.0924	0.0525			
100	rs142130958	19	11202306	ApoB	Ischemic stroke	-0.19965	0.01674	-0.0708	0.0249			
101	rs142130958	19	11190652	ApoB	Coronary heart disease	-0.19965	0.01674	-0.125647	0.016786			
102	rs142130958	19	11190652	ApoB	Myocardial infarction	-0.19965	0.01674	-0.100247	0.018695			
103	rs142130958	19	11202306	ApoB	Small vessel disease	-0.19965	0.01674	-0.013	0.0549			
104	rs142130958	19	11202306	ApoB	Cardioembolic stroke	-0.19965	0.01674	-0.0567	0.048			
105	rs143341434	1	54759547	ApoB	Myocardial infarction	-0.21853	0.02904	-0.0393462	0.060482			
106	rs143341434	1	54759547	ApoB	Coronary heart disease	-0.21853	0.02904	-0.016655	0.053934			
107	rs144064722	4	73406173	ApoB	Coronary heart disease	0.199001	0.03506	0.028313	0.031588			
108	rs144064722	4	73406173	ApoB	Myocardial infarction	0.199001	0.03506	0.032534	0.034056			
109	rs150617279	19	20139234	ApoB	Coronary heart disease	-0.11238	0.01769	0.035103	0.023945			
110	rs150617279	19	20139234	ApoB	Myocardial infarction	-0.11238	0.01769	0.0521612	0.026788			
111	rs17395160	1	55085141	ApoB	Myocardial infarction	-0.07105	0.01234	-0.013664	0.01264	FAM151A	LOC645436	C1orf175
112	rs17395160	1	55085141	ApoB	Ischemic stroke	-0.07105	0.01234	-0.0578	0.0207	FAM151A	LOC645436	C1orf175
113	rs17395160	1	55085141	ApoB	Cardioembolic stroke	-0.07105	0.01234	-0.0181	0.0396	FAM151A	LOC645436	C1orf175
114	rs17395160	1	55085141	ApoB	Small vessel disease	-0.07105	0.01234	-0.053	0.0447	FAM151A	LOC645436	C1orf175
115	rs17395160	1	55085141	ApoB	Large vessel disease	-0.07105	0.01234	-0.1123	0.0423	FAM151A	LOC645436	C1orf175
116	rs17395160	1	55085141	ApoB	Coronary heart disease	-0.07105	0.01234	-0.012783	0.011336	FAM151A	LOC645436	C1orf175
117	rs1883711	20	39179822	ApoB	Coronary heart disease	0.144092	0.02526	0.133601	0.034913		HSPEP1	MAFB
118	rs1883711	20	39179822	ApoB	Cardioembolic stroke	0.144092	0.02526	0.178	0.205		HSPEP1	MAFB
119	rs1883711	20	39179822	ApoB	Myocardial infarction	0.144092	0.02526	0.0985739	0.040277		HSPEP1	MAFB
120	rs207179	1	55788230	ApoB	Ischemic stroke	0.108802	0.01744	-0.0057	0.0275		LOC645506	GOT2L1
121	rs207179	1	55788230	ApoB	Cardioembolic stroke	0.108802	0.01744	-0.0576	0.0547		LOC645506	GOT2L1

122	rs207179	1	55788230	ApoB	Small vessel disease	0.108802	0.01744	-0.0849	0.06		LOC645506	GOT2L1
123	rs207179	1	55788230	ApoB	Coronary heart disease	0.108802	0.01744	-0.007173	0.017729		LOC645506	GOT2L1
124	rs207179	1	55788230	ApoB	Myocardial infarction	0.108802	0.01744	-0.00771559	0.019203		LOC645506	GOT2L1
125	rs207179	1	55788230	ApoB	Large vessel disease	0.108802	0.01744	-0.0055	0.0592		LOC645506	GOT2L1
126	rs2495477	1	55518467	ApoB	Myocardial infarction	-0.0619	0.01101	-0.0362507	0.011685	PCSK9	BSND	USP24
127	rs2495477	1	55518467	ApoB	Coronary heart disease	-0.0619	0.01101	-0.035937	0.010473	PCSK9	BSND	USP24
128	rs2980875	8	126481747	ApoB	Small vessel disease	-0.06968	0.00999	-0.0521	0.0356		TRIB1	LOC100130231
129	rs2980875	8	126481747	ApoB	Myocardial infarction	-0.06968	0.00999	-0.0432531	0.010383		TRIB1	LOC100130231
130	rs2980875	8	126481747	ApoB	Large vessel disease	-0.06968	0.00999	-0.0391	0.0344		TRIB1	LOC100130231
131	rs2980875	8	126481747	ApoB	Ischemic stroke	-0.06968	0.00999	-0.0207	0.0162		TRIB1	LOC100130231
132	rs2980875	8	126481747	ApoB	Cardioembolic stroke	-0.06968	0.00999	0.0188	0.032		TRIB1	LOC100130231
133	rs2980875	8	126481747	ApoB	Coronary heart disease	-0.06968	0.00999	-0.042618	0.0093		TRIB1	LOC100130231
134	rs4722043	7	21697529	ApoB	Coronary heart disease	-0.06594	0.01067	0.000996	0.010127	DNAH11	SP4	CDCA7L
135	rs4722043	7	21697529	ApoB	Myocardial infarction	-0.06594	0.01067	-0.00052221	0.011319	DNAH11	SP4	CDCA7L
136	rs62123891	2	21083052	ApoB	Coronary heart disease	-0.09929	0.01522	-0.019138	0.015934		C2orf43	APOB
137	rs62123891	2	21083052	ApoB	Myocardial infarction	-0.09929	0.01522	-0.00474096	0.017913		C2orf43	APOB
138	rs629301	1	109818306	ApoB	Coronary heart disease	0.090052	0.01219	0.101444	0.011423	CELSR2	SARS	PSRC1
139	rs629301	1	109818306	ApoB	Myocardial infarction	0.090052	0.01219	0.0875246	0.012685	CELSR2	SARS	PSRC1
140	rs629301	1	109818306	ApoB	Ischemic stroke	0.090052	0.01219	0.0022	0.0191	CELSR2	SARS	PSRC1
141	rs629301	1	109818306	ApoB	Cardioembolic stroke	0.090052	0.01219	0.0349	0.0376	CELSR2	SARS	PSRC1
142	rs629301	1	109818306	ApoB	Small vessel disease	0.090052	0.01219	-0.0002	0.0422	CELSR2	SARS	PSRC1
143	rs629301	1	109818306	ApoB	Large vessel disease	0.090052	0.01219	0.1	0.0414	CELSR2	SARS	PSRC1
144	rs635634	9	136155000	ApoB	Coronary heart disease	0.073971	0.01261	0.077157	0.011712		ABO	LOC653163
145	rs635634	9	136155000	ApoB	Myocardial infarction	0.073971	0.01261	0.10933	0.013093		ABO	LOC653163
146	rs6756629	2	44065090	ApoB	Cardioembolic stroke	-0.11325	0.01857	-0.1936	0.069	ABCG5 ABCG8	DYNC2LI1	ABCG8
147	rs6756629	2	44065090	ApoB	Myocardial infarction	-0.11325	0.01857	-0.0798551	0.023391	ABCG5 ABCG8	DYNC2LI1	ABCG8
148	rs6756629	2	44065090	ApoB	Ischemic stroke	-0.11325	0.01857	-0.1013	0.0335	ABCG5 ABCG8	DYNC2LI1	ABCG8
149	rs6756629	2	44065090	ApoB	Large vessel disease	-0.11325	0.01857	-0.1785	0.074	ABCG5 ABCG8	DYNC2LI1	ABCG8
150	rs6756629	2	44065090	ApoB	Small vessel disease	-0.11325	0.01857	-0.0699	0.0738	ABCG5 ABCG8	DYNC2LI1	ABCG8
151	rs6756629	2	44065090	ApoB	Coronary heart disease	-0.11325	0.01857	-0.098614	0.020976	ABCG5 ABCG8	DYNC2LI1	ABCG8
152	rs7256200	19	45415935	ApoB	Myocardial infarction	0.177158	0.01443	0.0922879	0.017993	APOC1	APOE	APOC1
153	rs7256200	19	45415935	ApoB	Coronary heart disease	0.177158	0.01443	0.09126	0.016203	APOC1	APOE	APOC1
154	rs7412	19	45412079	ApoB	Myocardial infarction	-0.42755	0.02598	-0.122218	0.023946	APOE LOC100129500 LOC100129500	TOMM40	APOC1

155	rs7412	19	45412079	ApoB	Coronary heart disease	-0.42755	0.02598	-0.137045	0.021092	APOE<br>LOC100129500<br>LOC100129500	TOMM40	APOC1
156	rs964184	11	116648917	ApoB	Small vessel disease	-0.16577	0.01427	-0.0253	0.0533	ZNF259	BUD13	ZNF259
157	rs964184	11	116648917	ApoB	Ischemic stroke	-0.16577	0.01427	-0.0074	0.024	ZNF259	BUD13	ZNF259
158	rs964184	11	116648917	ApoB	Large vessel disease	-0.16577	0.01427	-0.0184	0.0505	ZNF259	BUD13	ZNF259
159	rs964184	11	116648917	ApoB	Cardioembolic stroke	-0.16577	0.01427	-0.0127	0.0464	ZNF259	BUD13	ZNF259
160	rs964184	11	116648917	ApoB	Myocardial infarction	-0.16577	0.01427	-0.0487708	0.013873	ZNF259	BUD13	ZNF259
161	rs964184	11	116648917	ApoB	Coronary heart disease	-0.16577	0.01427	-0.049958	0.012399	ZNF259	BUD13	ZNF259
162	rs111894427	11	116773653	HDL.C	Large vessel disease	0.133814	0.02204	0.0769	0.0962			
163	rs111894427	11	116721405	HDL.C	Myocardial infarction	0.133814	0.02204	0.0070412	0.032006			
164	rs111894427	11	116721405	HDL.C	Coronary heart disease	0.133814	0.02204	-0.032958	0.029109			
165	rs111894427	11	116773653	HDL.C	Cardioembolic stroke	0.133814	0.02204	0.0443	0.0994			
166	rs111894427	11	116773653	HDL.C	Ischemic stroke	0.133814	0.02204	-0.0377	0.0497			
167	rs111894427	11	116773653	HDL.C	Small vessel disease	0.133814	0.02204	-0.153	0.1138			
168	rs117376818	15	58798961	HDL.C	Coronary heart disease	0.317736	0.05697	-0.009311	0.058182			
169	rs117376818	15	58798961	HDL.C	Myocardial infarction	0.317736	0.05697	-0.0221881	0.065023			
170	rs1461729	8	9187242	HDL.C	Large vessel disease	0.093989	0.01449	-0.0264	0.0594		PPP1R3B	LOC100129150
171	rs1461729	8	9187242	HDL.C	Myocardial infarction	0.093989	0.01449	0.00022829	0.017174		PPP1R3B	LOC100129150
172	rs1461729	8	9187242	HDL.C	Ischemic stroke	0.093989	0.01449	-0.0261	0.0274		PPP1R3B	LOC100129150
173	rs1461729	8	9187242	HDL.C	Small vessel disease	0.093989	0.01449	-0.0119	0.0603		PPP1R3B	LOC100129150
174	rs1461729	8	9187242	HDL.C	Cardioembolic stroke	0.093989	0.01449	-0.0919	0.0537		PPP1R3B	LOC100129150
175	rs1461729	8	9187242	HDL.C	Coronary heart disease	0.093989	0.01449	0.010384	0.015457		PPP1R3B	LOC100129150
176	rs16940810	15	59115159	HDL.C	Large vessel disease	0.186188	0.02974	0.0414	0.1051	FAM63B	ADAM10	LOC729208
177	rs16940810	15	59115159	HDL.C	Ischemic stroke	0.186188	0.02974	0.0256	0.0484	FAM63B	ADAM10	LOC729208
178	rs16940810	15	59115159	HDL.C	Small vessel disease	0.186188	0.02974	0.0854	0.1187	FAM63B	ADAM10	LOC729208
179	rs16940810	15	59115159	HDL.C	Cardioembolic stroke	0.186188	0.02974	0.1429	0.0849	FAM63B	ADAM10	LOC729208
180	rs16940810	15	59115159	HDL.C	Coronary heart disease	0.186188	0.02974	0.045851	0.028452	FAM63B	ADAM10	LOC729208
181	rs16940810	15	59115159	HDL.C	Myocardial infarction	0.186188	0.02974	0.0114989	0.031765	FAM63B	ADAM10	LOC729208
182	rs174594	11	61623140	HDL.C	Cardioembolic stroke	0.068823	0.01028	0.0366	0.0374	FADS2	LOC100131326	FADS3
183	rs174594	11	61619829	HDL.C	Coronary heart disease	0.068823	0.01028	0.016821	0.010328	FADS2	LOC100131326	FADS3
184	rs174594	11	61623140	HDL.C	Ischemic stroke	0.068823	0.01028	0.0452	0.0185	FADS2	LOC100131326	FADS3
185	rs174594	11	61623140	HDL.C	Large vessel disease	0.068823	0.01028	0.0832	0.0401	FADS2	LOC100131326	FADS3
186	rs174594	11	61619829	HDL.C	Myocardial infarction	0.068823	0.01028	0.0196856	0.0115	FADS2	LOC100131326	FADS3
187	rs174594	11	61623140	HDL.C	Small vessel disease	0.068823	0.01028	-0.0368	0.041	FADS2	LOC100131326	FADS3
188	rs1800961	20	43042364	HDL.C	Coronary heart disease	-0.15675	0.02649	-0.028051	0.028278	HNF4A	R3HDML	C20orf62
189	rs1800961	20	43042364	HDL.C	Myocardial infarction	-0.15675	0.02649	0.00598266	0.03186	HNF4A	R3HDML	C20orf62
190	rs1800961	20	43042364	HDL.C	Large vessel disease	-0.15675	0.02649	-0.0389	0.1014	HNF4A	R3HDML	C20orf62
191	rs1800961	20	43042364	HDL.C	Cardioembolic stroke	-0.15675	0.02649	0.0687	0.0868	HNF4A	R3HDML	C20orf62
192	rs1800961	20	43042364	HDL.C	Small vessel disease	-0.15675	0.02649	-0.0113	0.1052	HNF4A	R3HDML	C20orf62
193	rs1800961	20	43042364	HDL.C	Ischemic stroke	-0.15675	0.02649	-0.0368	0.044	HNF4A	R3HDML	C20orf62

194	rs1883025	9	107664301	HDL.C	Large vessel disease	-0.07606	0.01246	-0.0302	0.04	ABCA1	NIPSNAP3B	SLC44A1
195	rs1883025	9	107664301	HDL.C	Coronary heart disease	-0.07606	0.01246	-0.025887	0.010457	ABCA1	NIPSNAP3B	SLC44A1
196	rs1883025	9	107664301	HDL.C	Ischemic stroke	-0.07606	0.01246	0.021	0.0189	ABCA1	NIPSNAP3B	SLC44A1
197	rs1883025	9	107664301	HDL.C	Cardioembolic stroke	-0.07606	0.01246	-0.0129	0.0368	ABCA1	NIPSNAP3B	SLC44A1
198	rs1883025	9	107664301	HDL.C	Myocardial infarction	-0.07606	0.01246	-0.0258134	0.011584	ABCA1	NIPSNAP3B	SLC44A1
199	rs1883025	9	107664301	HDL.C	Small vessel disease	-0.07606	0.01246	0.1045	0.0409	ABCA1	NIPSNAP3B	SLC44A1
200	rs2070895	15	58723939	HDL.C	Coronary heart disease	0.130965	0.01164	0.037159	0.010782	LIPC	LOC441726	LIPC
201	rs2070895	15	58723939	HDL.C	Myocardial infarction	0.130965	0.01164	0.0413675	0.012105	LIPC	LOC441726	LIPC
202	rs2070895	15	58723939	HDL.C	Small vessel disease	0.130965	0.01164	0.0715	0.0449	LIPC	LOC441726	LIPC
203	rs2070895	15	58723939	HDL.C	Cardioembolic stroke	0.130965	0.01164	-0.0622	0.0419	LIPC	LOC441726	LIPC
204	rs2070895	15	58723939	HDL.C	Ischemic stroke	0.130965	0.01164	-0.0355	0.0209	LIPC	LOC441726	LIPC
205	rs2070895	15	58723939	HDL.C	Large vessel disease	0.130965	0.01164	-0.0729	0.0446	LIPC	LOC441726	LIPC
206	rs247617	16	56993324	HDL.C	Large vessel disease	0.227319	0.01078	0.0256	0.0369		HERPUD1	CETP
207	rs247617	16	56993324	HDL.C	Cardioembolic stroke	0.227319	0.01078	0.0074	0.0345		HERPUD1	CETP
208	rs247617	16	56993324	HDL.C	Small vessel disease	0.227319	0.01078	0.0027	0.0382		HERPUD1	CETP
209	rs247617	16	56990716	HDL.C	Coronary heart disease	0.227319	0.01078	-0.030884	0.010212		HERPUD1	CETP
210	rs247617	16	56993324	HDL.C	Ischemic stroke	0.227319	0.01078	0.0102	0.0175		HERPUD1	CETP
211	rs247617	16	56990716	HDL.C	Myocardial infarction	0.227319	0.01078	-0.0261377	0.011415		HERPUD1	CETP
212	rs261291	15	58683366	HDL.C	Cardioembolic stroke	0.128746	0.0102	-0.0091	0.0317		LOC441726	LIPC
213	rs261291	15	58683366	HDL.C	Large vessel disease	0.128746	0.0102	-0.016	0.0343		LOC441726	LIPC
214	rs261291	15	58683366	HDL.C	Ischemic stroke	0.128746	0.0102	-0.0215	0.0161		LOC441726	LIPC
215	rs261291	15	58683366	HDL.C	Small vessel disease	0.128746	0.0102	-0.0405	0.0359		LOC441726	LIPC
216	rs261291	15	58680178	HDL.C	Myocardial infarction	0.128746	0.0102	0.00516156	0.010716		LOC441726	LIPC
217	rs261291	15	58680178	HDL.C	Coronary heart disease	0.128746	0.0102	0.012987	0.009619		LOC441726	LIPC
218	rs291	8	19815852	HDL.C	Coronary heart disease	0.096436	0.01168	-0.04804	0.011493	LPL	INTS10	SLC18A1
219	rs291	8	19815852	HDL.C	Small vessel disease	0.096436	0.01168	0.0029	0.0429	LPL	INTS10	SLC18A1
220	rs291	8	19815852	HDL.C	Myocardial infarction	0.096436	0.01168	-0.0348685	0.012907	LPL	INTS10	SLC18A1
221	rs291	8	19815852	HDL.C	Cardioembolic stroke	0.096436	0.01168	-0.0356	0.0388	LPL	INTS10	SLC18A1
222	rs291	8	19815852	HDL.C	Large vessel disease	0.096436	0.01168	-0.0674	0.0414	LPL	INTS10	SLC18A1
223	rs291	8	19815852	HDL.C	Ischemic stroke	0.096436	0.01168	0.002	0.0194	LPL	INTS10	SLC18A1
224	rs4149307	9	107589744	HDL.C	Myocardial infarction	0.096471	0.01725	0.0204321	0.013422	ABCA1	NIPSNAP3B	SLC44A1
225	rs4149307	9	107589744	HDL.C	Coronary heart disease	0.096471	0.01725	0.027493	0.011999	ABCA1	NIPSNAP3B	SLC44A1
226	rs5880	16	57015091	HDL.C	Myocardial infarction	-0.23068	0.03155	0.0247342	0.0245	CETP	HERPUD1	LOC100130044
227	rs5880	16	57015091	HDL.C	Small vessel disease	-0.23068	0.03155	-0.0283	0.0961	CETP	HERPUD1	LOC100130044
228	rs5880	16	57015091	HDL.C	Ischemic stroke	-0.23068	0.03155	0.0364	0.0422	CETP	HERPUD1	LOC100130044
229	rs5880	16	57015091	HDL.C	Large vessel disease	-0.23068	0.03155	-0.0992	0.0884	CETP	HERPUD1	LOC100130044
230	rs5880	16	57015091	HDL.C	Cardioembolic stroke	-0.23068	0.03155	0.0874	0.0838	CETP	HERPUD1	LOC100130044
231	rs5880	16	57015091	HDL.C	Coronary heart disease	-0.23068	0.03155	0.007277	0.022421	CETP	HERPUD1	LOC100130044
232	rs6065904	20	44534651	HDL.C	Cardioembolic stroke	-0.06627	0.0117	-0.005	0.0387	PLTP	CTSA	FLJ40606
233	rs6065904	20	44534651	HDL.C	Large vessel disease	-0.06627	0.0117	-0.0002	0.042	PLTP	CTSA	FLJ40606
234	rs6065904	20	44534651	HDL.C	Myocardial infarction	-0.06627	0.0117	-0.0231151	0.01205	PLTP	CTSA	FLJ40606

235	rs6065904	20	44534651	HDL.C	Coronary heart disease	-0.06627	0.0117	-0.019307	0.010831	PLTP	CTSA	FLJ40606
236	rs6065904	20	44534651	HDL.C	Small vessel disease	-0.06627	0.0117	0.039	0.0432	PLTP	CTSA	FLJ40606
237	rs6065904	20	44534651	HDL.C	Ischemic stroke	-0.06627	0.0117	-0.0149	0.0197	PLTP	CTSA	FLJ40606
238	rs6507939	18	47176261	HDL.C	Myocardial infarction	0.092625	0.01346	-0.0174663	0.015114		LIPG	LOC100129143
239	rs6507939	18	47176261	HDL.C	Large vessel disease	0.092625	0.01346	0.0528	0.0514		LIPG	LOC100129143
240	rs6507939	18	47176261	HDL.C	Cardioembolic stroke	0.092625	0.01346	0.0079	0.0479		LIPG	LOC100129143
241	rs6507939	18	47176261	HDL.C	Ischemic stroke	0.092625	0.01346	-0.0068	0.0237		LIPG	LOC100129143
242	rs6507939	18	47176261	HDL.C	Small vessel disease	0.092625	0.01346	-0.0005	0.0522		LIPG	LOC100129143
243	rs6507939	18	47176261	HDL.C	Coronary heart disease	0.092625	0.01346	-0.024621	0.013496		LIPG	LOC100129143
244	rs67053123	12	125380232	HDL.C	Ischemic stroke	0.080931	0.01455	-0.0034	0.0257			
245	rs67053123	12	125380232	HDL.C	Small vessel disease	0.080931	0.01455	0.0385	0.0581			
246	rs67053123	12	125353810	HDL.C	Coronary heart disease	0.080931	0.01455	0.008938	0.014264			
247	rs67053123	12	125353810	HDL.C	Myocardial infarction	0.080931	0.01455	0.0278404	0.015985			
248	rs67053123	12	125380232	HDL.C	Cardioembolic stroke	0.080931	0.01455	0.0483	0.051			
249	rs67053123	12	125380232	HDL.C	Large vessel disease	0.080931	0.01455	-0.0563	0.0558			
250	rs75835816	8	19885513	HDL.C	Myocardial infarction	-0.29237	0.03814	0.0295779	0.050057			
251	rs75835816	8	19885513	HDL.C	Coronary heart disease	-0.29237	0.03814	0.066618	0.043542			
252	rs113105798	15	59301460	IDL.TG	Myocardial infarction	0.197768	0.0355	-0.0400261	0.044667			
253	rs113105798	15	59301460	IDL.TG	Coronary heart disease	0.197768	0.0355	-0.055219	0.040092			
254	rs113531395	17	4886829	IDL.TG	Coronary heart disease	-0.2459	0.03628	-0.046539	0.061719			
255	rs113531395	17	4886829	IDL.TG	Myocardial infarction	-0.2459	0.03628	-0.0573797	0.067105			
256	rs115849089	8	19912370	IDL.TG	Myocardial infarction	-0.09785	0.01719	-0.0667741	0.017021			
257	rs115849089	8	19912370	IDL.TG	Coronary heart disease	-0.09785	0.01719	-0.057989	0.015294			
258	rs11633043	15	58837722	IDL.TG	Coronary heart disease	0.078896	0.0142	0.009123	0.01474	LIPC	LOC441726	ADAM10
259	rs11633043	15	58837722	IDL.TG	Myocardial infarction	0.078896	0.0142	-0.00253185	0.016463	LIPC	LOC441726	ADAM10
260	rs1168041	1	62960250	IDL.TG	Ischemic stroke	0.083815	0.01173	-0.0095	0.0178	DOCK7	USP1	ANGPTL3
261	rs1168041	1	62960250	IDL.TG	Coronary heart disease	0.083815	0.01173	0.011014	0.01011	DOCK7	USP1	ANGPTL3
262	rs1168041	1	62960250	IDL.TG	Large vessel disease	0.083815	0.01173	-0.0152	0.0373	DOCK7	USP1	ANGPTL3
263	rs1168041	1	62960250	IDL.TG	Small vessel disease	0.083815	0.01173	-0.0781	0.0389	DOCK7	USP1	ANGPTL3
264	rs1168041	1	62960250	IDL.TG	Myocardial infarction	0.083815	0.01173	0.00067632	0.011148	DOCK7	USP1	ANGPTL3
265	rs1168041	1	62960250	IDL.TG	Cardioembolic stroke	0.083815	0.01173	-0.0236	0.0353	DOCK7	USP1	ANGPTL3
266	rs144064722	4	73406173	IDL.TG	Myocardial infarction	0.24965	0.03404	0.032534	0.034056			
267	rs144064722	4	73406173	IDL.TG	Coronary heart disease	0.24965	0.03404	0.028313	0.031588			
268	rs146842281	15	59356659	IDL.TG	Myocardial infarction	0.145519	0.02193	0.0331942	0.039409			
269	rs146842281	15	59356659	IDL.TG	Coronary heart disease	0.145519	0.02193	0.025121	0.033363			
270	rs1532085	15	58683366	IDL.TG	Myocardial infarction	-0.15608	0.01048	-0.00988838	0.010417		LOC441726	LIPC
271	rs1532085	15	58683366	IDL.TG	Cardioembolic stroke	-0.15608	0.01048	0.0091	0.0317		LOC441726	LIPC
272	rs1532085	15	58683366	IDL.TG	Ischemic stroke	-0.15608	0.01048	0.0215	0.0161		LOC441726	LIPC
273	rs1532085	15	58683366	IDL.TG	Small vessel disease	-0.15608	0.01048	0.0405	0.0359		LOC441726	LIPC
274	rs1532085	15	58683366	IDL.TG	Coronary heart disease	-0.15608	0.01048	-0.01812	0.009354		LOC441726	LIPC
275	rs1532085	15	58683366	IDL.TG	Large vessel disease	-0.15608	0.01048	0.016	0.0343		LOC441726	LIPC

276	rs157594	19	45425175	IDL.TG	Coronary heart disease	0.125692	0.01172	0.021906	0.011888	LOC100129500	APOC1	APOC4
277	rs157594	19	45425175	IDL.TG	Myocardial infarction	0.125692	0.01172	0.0265114	0.013237	LOC100129500	APOC1	APOC4
278	rs1883711	20	39179822	IDL.TG	Coronary heart disease	0.154938	0.02472	0.133601	0.034913		HSPEP1	MAFB
279	rs1883711	20	39179822	IDL.TG	Myocardial infarction	0.154938	0.02472	0.0985739	0.040277		HSPEP1	MAFB
280	rs1883711	20	39179822	IDL.TG	Cardioembolic stroke	0.154938	0.02472	0.178	0.205		HSPEP1	MAFB
281	rs247617	16	56993324	IDL.TG	Cardioembolic stroke	-0.08426	0.01147	0.0074	0.0345		HERPUD1	CETP
282	rs247617	16	56993324	IDL.TG	Small vessel disease	-0.08426	0.01147	0.0027	0.0382		HERPUD1	CETP
283	rs247617	16	56990716	IDL.TG	Myocardial infarction	-0.08426	0.01147	-0.0261377	0.011415		HERPUD1	CETP
284	rs247617	16	56990716	IDL.TG	Coronary heart disease	-0.08426	0.01147	-0.030884	0.010212		HERPUD1	CETP
285	rs247617	16	56993324	IDL.TG	Large vessel disease	-0.08426	0.01147	0.0256	0.0369		HERPUD1	CETP
286	rs247617	16	56993324	IDL.TG	Ischemic stroke	-0.08426	0.01147	0.0102	0.0175		HERPUD1	CETP
287	rs261334	15	58726744	IDL.TG	Cardioembolic stroke	-0.19736	0.01226	0.0623	0.0423	LIPC	LOC441726	ADAM10
288	rs261334	15	58726744	IDL.TG	Ischemic stroke	-0.19736	0.01226	0.0383	0.0211	LIPC	LOC441726	ADAM10
289	rs261334	15	58726744	IDL.TG	Large vessel disease	-0.19736	0.01226	0.0677	0.0452	LIPC	LOC441726	ADAM10
290	rs261334	15	58726744	IDL.TG	Coronary heart disease	-0.19736	0.01226	-0.03756	0.011043	LIPC	LOC441726	ADAM10
291	rs261334	15	58726744	IDL.TG	Myocardial infarction	-0.19736	0.01226	-0.0402744	0.012395	LIPC	LOC441726	ADAM10
292	rs261334	15	58726744	IDL.TG	Small vessel disease	-0.19736	0.01226	-0.0739	0.0451	LIPC	LOC441726	ADAM10
293	rs312030	2	21462743	IDL.TG	Myocardial infarction	0.11074	0.01814	-0.0151187	0.021444		LOC100129278	LOC645949
294	rs312030	2	21462743	IDL.TG	Coronary heart disease	0.11074	0.01814	0.007792	0.019535		LOC100129278	LOC645949
295	rs34722314	2	21303470	IDL.TG	Large vessel disease	-0.12461	0.01498	0.0482	0.05		APOB	LOC100129278
296	rs34722314	2	21271707	IDL.TG	Myocardial infarction	-0.12461	0.01498	-0.0465062	0.015487		APOB	LOC100129278
297	rs34722314	2	21303470	IDL.TG	Ischemic stroke	-0.12461	0.01498	0.0079	0.024		APOB	LOC100129278
298	rs34722314	2	21303470	IDL.TG	Cardioembolic stroke	-0.12461	0.01498	0.035	0.0475		APOB	LOC100129278
299	rs34722314	2	21303470	IDL.TG	Small vessel disease	-0.12461	0.01498	0.0056	0.0525		APOB	LOC100129278
300	rs34722314	2	21271707	IDL.TG	Coronary heart disease	-0.12461	0.01498	-0.06419	0.014243		APOB	LOC100129278
301	rs4075673	2	21150787	IDL.TG	Myocardial infarction	-0.10248	0.01048	-0.0115867	0.010676			
302	rs4075673	2	21150787	IDL.TG	Coronary heart disease	-0.10248	0.01048	-0.013347	0.009722			
303	rs4609471	1	55493584	IDL.TG	Coronary heart disease	-0.25447	0.02976	-0.088176	0.037828		BSND	PCSK9
304	rs4609471	1	55493584	IDL.TG	Myocardial infarction	-0.25447	0.02976	-0.110147	0.042779		BSND	PCSK9
305	rs55966152	15	58561006	IDL.TG	Myocardial infarction	-0.09174	0.01607	-0.0291511	0.013851		LOC441726	LIPC
306	rs55966152	15	58561006	IDL.TG	Coronary heart disease	-0.09174	0.01607	-0.022246	0.012545		LOC441726	LIPC
307	rs58542926	19	19610596	IDL.TG	Cardioembolic stroke	-0.14101	0.02107	0.1322	0.0595	TM6SF2	HAPLN4	SF4
308	rs58542926	19	19610596	IDL.TG	Ischemic stroke	-0.14101	0.02107	0.0224	0.0304	TM6SF2	HAPLN4	SF4
309	rs58542926	19	19379549	IDL.TG	Coronary heart disease	-0.14101	0.02107	-0.051253	0.018343	TM6SF2	HAPLN4	SF4
310	rs58542926	19	19610596	IDL.TG	Large vessel disease	-0.14101	0.02107	0.0518	0.0641	TM6SF2	HAPLN4	SF4
311	rs58542926	19	19610596	IDL.TG	Small vessel disease	-0.14101	0.02107	0.0136	0.0675	TM6SF2	HAPLN4	SF4
312	rs58542926	19	19379549	IDL.TG	Myocardial infarction	-0.14101	0.02107	-0.0459691	0.020412	TM6SF2	HAPLN4	SF4
313	rs61999891	15	58299599	IDL.TG	Coronary heart disease	0.102936	0.01736	-0.022129	0.015103	ALDH1A2<br>ALDH1A2	LOC100132719	AQP9
314	rs61999891	15	58299599	IDL.TG	Myocardial infarction	0.102936	0.01736	0.00774116	0.016926	ALDH1A2<br>ALDH1A2	LOC100132719	AQP9

315	rs6511720	19	11202306	IDL.TG	Ischemic stroke	-0.17567	0.01733	-0.0708	0.0249	LDLR	SMARCA4	SPC24
316	rs6511720	19	11202306	IDL.TG	Large vessel disease	-0.17567	0.01733	-0.0924	0.0525	LDLR	SMARCA4	SPC24
317	rs6511720	19	11202306	IDL.TG	Myocardial infarction	-0.17567	0.01733	-0.100786	0.01894	LDLR	SMARCA4	SPC24
318	rs6511720	19	11202306	IDL.TG	Coronary heart disease	-0.17567	0.01733	-0.125298	0.016945	LDLR	SMARCA4	SPC24
319	rs6511720	19	11202306	IDL.TG	Cardioembolic stroke	-0.17567	0.01733	-0.0567	0.048	LDLR	SMARCA4	SPC24
320	rs6511720	19	11202306	IDL.TG	Small vessel disease	-0.17567	0.01733	-0.013	0.0549	LDLR	SMARCA4	SPC24
321	rs660240	1	109817838	IDL.TG	Cardioembolic stroke	0.078525	0.01248	0.0225	0.0383	CELSR2	SARS	PSRC1
322	rs660240	1	109817838	IDL.TG	Ischemic stroke	0.078525	0.01248	-0.0067	0.0194	CELSR2	SARS	PSRC1
323	rs660240	1	109817838	IDL.TG	Myocardial infarction	0.078525	0.01248	0.0881729	0.012683	CELSR2	SARS	PSRC1
324	rs660240	1	109817838	IDL.TG	Coronary heart disease	0.078525	0.01248	0.102043	0.011529	CELSR2	SARS	PSRC1
325	rs660240	1	109817838	IDL.TG	Large vessel disease	0.078525	0.01248	0.0982	0.042	CELSR2	SARS	PSRC1
326	rs660240	1	109817838	IDL.TG	Small vessel disease	0.078525	0.01248	-0.0169	0.0426	CELSR2	SARS	PSRC1
327	rs72660594	1	55636240	IDL.TG	Coronary heart disease	-0.27171	0.02881	-0.080262	0.046373			
328	rs72660594	1	55636240	IDL.TG	Myocardial infarction	-0.27171	0.02881	-0.0986201	0.051013			
329	rs7412	19	45412079	IDL.TG	Myocardial infarction	-0.26322	0.02608	-0.122218	0.023946	APOE LOC100129500 LOC100129500	TOMM40	APOC1
330	rs7412	19	45412079	IDL.TG	Coronary heart disease	-0.26322	0.02608	-0.137045	0.021092	APOE LOC100129500 LOC100129500	TOMM40	APOC1
331	rs75679663	17	4667972	IDL.TG	Coronary heart disease	-0.22281	0.03874	-0.011031	0.066703			
332	rs75679663	17	4667972	IDL.TG	Myocardial infarction	-0.22281	0.03874	0.0280457	0.073714			
333	rs79225634	5	74619639	IDL.TG	Coronary heart disease	0.062054	0.01082	0.024313	0.009685			
334	rs79225634	5	74619639	IDL.TG	Myocardial infarction	0.062054	0.01082	0.0202878	0.010617			
335	rs9302635	16	72144174	IDL.TG	Myocardial infarction	-0.0778	0.01347	-0.0166367	0.014983	DHX38	TXNL4B	PMFBP1
336	rs9302635	16	72144174	IDL.TG	Ischemic stroke	-0.0778	0.01347	-0.0059	0.0221	DHX38	TXNL4B	PMFBP1
337	rs9302635	16	72144174	IDL.TG	Coronary heart disease	-0.0778	0.01347	-0.007167	0.013264	DHX38	TXNL4B	PMFBP1
338	rs9302635	16	72144174	IDL.TG	Large vessel disease	-0.0778	0.01347	-0.01	0.0464	DHX38	TXNL4B	PMFBP1
339	rs9302635	16	72144174	IDL.TG	Cardioembolic stroke	-0.0778	0.01347	-0.038	0.0446	DHX38	TXNL4B	PMFBP1
340	rs9302635	16	72144174	IDL.TG	Small vessel disease	-0.0778	0.01347	0.0143	0.0489	DHX38	TXNL4B	PMFBP1
341	rs964184	11	116648917	IDL.TG	Cardioembolic stroke	-0.14886	0.01457	-0.0127	0.0464	ZNF259	BUD13	ZNF259
342	rs964184	11	116648917	IDL.TG	Coronary heart disease	-0.14886	0.01457	-0.049958	0.012399	ZNF259	BUD13	ZNF259
343	rs964184	11	116648917	IDL.TG	Ischemic stroke	-0.14886	0.01457	-0.0074	0.024	ZNF259	BUD13	ZNF259
344	rs964184	11	116648917	IDL.TG	Small vessel disease	-0.14886	0.01457	-0.0253	0.0533	ZNF259	BUD13	ZNF259
345	rs964184	11	116648917	IDL.TG	Large vessel disease	-0.14886	0.01457	-0.0184	0.0505	ZNF259	BUD13	ZNF259
346	rs964184	11	116648917	IDL.TG	Myocardial infarction	-0.14886	0.01457	-0.0487708	0.013873	ZNF259	BUD13	ZNF259
347	rs10455872	6	161010118	L.VLDL.TG	Cardioembolic stroke	-0.16521	0.02808	0.0167	0.0957	LPA	LPAL2	PLG
348	rs10455872	6	161010118	L.VLDL.TG	Small vessel disease	-0.16521	0.02808	-0.0807	0.1015	LPA	LPAL2	PLG
349	rs10455872	6	161010118	L.VLDL.TG	Coronary heart disease	-0.16521	0.02808	0.318598	0.024399	LPA	LPAL2	PLG
350	rs10455872	6	161010118	L.VLDL.TG	Large vessel disease	-0.16521	0.02808	0.227	0.0906	LPA	LPAL2	PLG
351	rs10455872	6	161010118	L.VLDL.TG	Myocardial infarction	-0.16521	0.02808	0.284774	0.026592	LPA	LPAL2	PLG

352	rs10455872	6	161010118	L.VLDL.TG	Ischemic stroke	-0.16521	0.02808	0.0418	0.045	LPA	LPAL2	PLG
353	rs1260326	2	27730940	L.VLDL.TG	Small vessel disease	-0.09421	0.01028	-0.0441	0.0353	GCKR	FNDC4	LOC100130981
354	rs1260326	2	27730940	L.VLDL.TG	Coronary heart disease	-0.09421	0.01028	0.003257	0.00962	GCKR	FNDC4	LOC100130981
355	rs1260326	2	27730940	L.VLDL.TG	Cardioembolic stroke	-0.09421	0.01028	0.0401	0.0314	GCKR	FNDC4	LOC100130981
356	rs1260326	2	27730940	L.VLDL.TG	Ischemic stroke	-0.09421	0.01028	0.012	0.0162	GCKR	FNDC4	LOC100130981
357	rs1260326	2	27730940	L.VLDL.TG	Large vessel disease	-0.09421	0.01028	0.0392	0.0341	GCKR	FNDC4	LOC100130981
358	rs1260326	2	27730940	L.VLDL.TG	Myocardial infarction	-0.09421	0.01028	0.00111651	0.010666	GCKR	FNDC4	LOC100130981
359	rs34346326	7	73016181	L.VLDL.TG	Myocardial infarction	-0.1103	0.01316	-0.00956639	0.014338	MLXIPL<br>MLXIPL<br>MLXIPL	TBL2	VPS37D
360	rs34346326	7	73016181	L.VLDL.TG	Coronary heart disease	-0.1103	0.01316	-0.010364	0.012808	MLXIPL<br>MLXIPL<br>MLXIPL	TBL2	VPS37D
361	rs4350231	1	62922660	L.VLDL.TG	Ischemic stroke	-0.07104	0.01074	0.018	0.017	DOCK7	USP1	ANGPTL3
362	rs4350231	1	62922660	L.VLDL.TG	Cardioembolic stroke	-0.07104	0.01074	0.027	0.0336	DOCK7	USP1	ANGPTL3
363	rs4350231	1	62922660	L.VLDL.TG	Large vessel disease	-0.07104	0.01074	0.0145	0.0361	DOCK7	USP1	ANGPTL3
364	rs4350231	1	62922660	L.VLDL.TG	Small vessel disease	-0.07104	0.01074	0.0809	0.0375	DOCK7	USP1	ANGPTL3
365	rs4350231	1	62922660	L.VLDL.TG	Coronary heart disease	-0.07104	0.01074	-0.01175	0.00975	DOCK7	USP1	ANGPTL3
366	rs4350231	1	62922660	L.VLDL.TG	Myocardial infarction	-0.07104	0.01074	-0.00466521	0.010839	DOCK7	USP1	ANGPTL3
367	rs438811	19	45416741	L.VLDL.TG	Myocardial infarction	0.089567	0.01217	0.0249193	0.014388	LOC100129500	APOE	APOC1
368	rs438811	19	45416741	L.VLDL.TG	Coronary heart disease	0.089567	0.01217	0.013252	0.012834	LOC100129500	APOE	APOC1
369	rs72999033	19	19407718	L.VLDL.TG	Small vessel disease	-0.13285	0.02114	0.0398	0.0744			
370	rs72999033	19	19407718	L.VLDL.TG	Ischemic stroke	-0.13285	0.02114	0.0212	0.0335			
371	rs72999033	19	19366632	L.VLDL.TG	Myocardial infarction	-0.13285	0.02114	-0.0370382	0.023873			
372	rs72999033	19	19407718	L.VLDL.TG	Cardioembolic stroke	-0.13285	0.02114	0.0972	0.0645			
373	rs72999033	19	19366632	L.VLDL.TG	Coronary heart disease	-0.13285	0.02114	-0.051959	0.021377			
374	rs72999033	19	19407718	L.VLDL.TG	Large vessel disease	-0.13285	0.02114	0.1255	0.0702			
375	rs76975037	8	19851508	L.VLDL.TG	Myocardial infarction	-0.15445	0.01786	-0.0665203	0.017954			
376	rs76975037	8	19852134	L.VLDL.TG	Cardioembolic stroke	-0.15445	0.01786	0.0885	0.0497			
377	rs76975037	8	19852134	L.VLDL.TG	Ischemic stroke	-0.15445	0.01786	0.0376	0.0262			
378	rs76975037	8	19851508	L.VLDL.TG	Coronary heart disease	-0.15445	0.01786	-0.053571	0.016022			
379	rs76975037	8	19852134	L.VLDL.TG	Small vessel disease	-0.15445	0.01786	0.0289	0.0596			
380	rs76975037	8	19852134	L.VLDL.TG	Large vessel disease	-0.15445	0.01786	0.0167	0.0536			
381	rs9472125	6	43756169	L.VLDL.TG	Myocardial infarction	-0.09079	0.01626	-0.0503078	0.021507		VEGFA	LOC100132354
382	rs9472125	6	43756169	L.VLDL.TG	Coronary heart disease	-0.09079	0.01626	-0.054856	0.019233		VEGFA	LOC100132354
383	rs964184	11	116648917	L.VLDL.TG	Cardioembolic stroke	-0.20631	0.01408	-0.0127	0.0464	ZNF259	BUD13	ZNF259
384	rs964184	11	116648917	L.VLDL.TG	Myocardial infarction	-0.20631	0.01408	-0.0487708	0.013873	ZNF259	BUD13	ZNF259
385	rs964184	11	116648917	L.VLDL.TG	Ischemic stroke	-0.20631	0.01408	-0.0074	0.024	ZNF259	BUD13	ZNF259
386	rs964184	11	116648917	L.VLDL.TG	Coronary heart disease	-0.20631	0.01408	-0.049958	0.012399	ZNF259	BUD13	ZNF259
387	rs964184	11	116648917	L.VLDL.TG	Small vessel disease	-0.20631	0.01408	-0.0253	0.0533	ZNF259	BUD13	ZNF259
388	rs964184	11	116648917	L.VLDL.TG	Large vessel disease	-0.20631	0.01408	-0.0184	0.0505	ZNF259	BUD13	ZNF259

389	rs10056811	5	74605220	LDL.C	Cardioembolic stroke	0.08841	0.01036	-0.023	0.0348		ANKRD31	LOC728775
390	rs10056811	5	74605220	LDL.C	Small vessel disease	0.08841	0.01036	0.0149	0.0382		ANKRD31	LOC728775
391	rs10056811	5	74605220	LDL.C	Ischemic stroke	0.08841	0.01036	-0.0082	0.0176		ANKRD31	LOC728775
392	rs10056811	5	74605220	LDL.C	Large vessel disease	0.08841	0.01036	-0.0701	0.038		ANKRD31	LOC728775
393	rs10056811	5	74605220	LDL.C	Myocardial infarction	0.08841	0.01036	0.0221538	0.010655		ANKRD31	LOC728775
394	rs10056811	5	74605220	LDL.C	Coronary heart disease	0.08841	0.01036	0.025525	0.009749		ANKRD31	LOC728775
395	rs1081105	19	45412955	LDL.C	Myocardial infarction	0.232041	0.03866	0.142826	0.048309	APOE	APOE	APOC1
396	rs1081105	19	45412955	LDL.C	Coronary heart disease	0.232041	0.03866	0.086187	0.041158	APOE	APOE	APOC1
397	rs112635299	14	94838142	LDL.C	Myocardial infarction	0.232048	0.03966	-0.16874	0.054411			
398	rs112635299	14	94838142	LDL.C	Coronary heart disease	0.232048	0.03966	-0.168269	0.047485			
399	rs11591147	1	55505647	LDL.C	Myocardial infarction	-0.51524	0.03472	-0.354653	0.068555	PCSK9	BSND	USP24
400	rs11591147	1	55505647	LDL.C	Coronary heart disease	-0.51524	0.03472	-0.256502	0.057259	PCSK9	BSND	USP24
401	rs11878174	19	45723379	LDL.C	Coronary heart disease	0.075095	0.01166	0.028498	0.01273	EXOC3L2	BLOC1S3	LOC100132177
402	rs11878174	19	45723379	LDL.C	Myocardial infarction	0.075095	0.01166	0.0201506	0.014108	EXOC3L2	BLOC1S3	LOC100132177
403	rs12029058	1	55719042	LDL.C	Small vessel disease	-0.06599	0.01154	-0.0854	0.0518		LOC645506	GOT2L1
404	rs12029058	1	55719042	LDL.C	Ischemic stroke	-0.06599	0.01154	-0.0243	0.0232		LOC645506	GOT2L1
405	rs12029058	1	55717274	LDL.C	Myocardial infarction	-0.06599	0.01154	-0.027904	0.013167		LOC645506	GOT2L1
406	rs12029058	1	55717274	LDL.C	Coronary heart disease	-0.06599	0.01154	-0.028159	0.011885		LOC645506	GOT2L1
407	rs12029058	1	55719042	LDL.C	Cardioembolic stroke	-0.06599	0.01154	-0.0152	0.0455		LOC645506	GOT2L1
408	rs12029058	1	55719042	LDL.C	Large vessel disease	-0.06599	0.01154	-0.0199	0.0493		LOC645506	GOT2L1
409	rs142130958	19	11202306	LDL.C	Cardioembolic stroke	-0.23071	0.01633	-0.0567	0.048			
410	rs142130958	19	11202306	LDL.C	Small vessel disease	-0.23071	0.01633	-0.013	0.0549			
411	rs142130958	19	11202306	LDL.C	Large vessel disease	-0.23071	0.01633	-0.0924	0.0525			
412	rs142130958	19	11190652	LDL.C	Myocardial infarction	-0.23071	0.01633	-0.100247	0.018695			
413	rs142130958	19	11190652	LDL.C	Coronary heart disease	-0.23071	0.01633	-0.125647	0.016786			
414	rs142130958	19	11202306	LDL.C	Ischemic stroke	-0.23071	0.01633	-0.0708	0.0249			
415	rs143341434	1	54759547	LDL.C	Myocardial infarction	-0.2658	0.02841	-0.0393462	0.060482			
416	rs143341434	1	54759547	LDL.C	Coronary heart disease	-0.2658	0.02841	-0.016655	0.053934			
417	rs144064722	4	73406173	LDL.C	Myocardial infarction	0.241631	0.03401	0.032534	0.034056			
418	rs144064722	4	73406173	LDL.C	Coronary heart disease	0.241631	0.03401	0.028313	0.031588			
419	rs1712249	2	21385974	LDL.C	Coronary heart disease	0.062156	0.01114	0.039574	0.011268		LOC100129278	LOC645949
420	rs1712249	2	21386957	LDL.C	Ischemic stroke	0.062156	0.01114	0.0049	0.0209		LOC100129278	LOC645949
421	rs1712249	2	21386957	LDL.C	Large vessel disease	0.062156	0.01114	-0.0275	0.0441		LOC100129278	LOC645949
422	rs1712249	2	21386957	LDL.C	Cardioembolic stroke	0.062156	0.01114	0.004	0.0415		LOC100129278	LOC645949
423	rs1712249	2	21385974	LDL.C	Myocardial infarction	0.062156	0.01114	0.0313648	0.012366		LOC100129278	LOC645949
424	rs1712249	2	21386957	LDL.C	Small vessel disease	0.062156	0.01114	-0.0023	0.0462		LOC100129278	LOC645949
425	rs17395160	1	55085141	LDL.C	Coronary heart disease	-0.08501	0.01207	-0.012783	0.011336	FAM151A	LOC645436	C1orf175
426	rs17395160	1	55085141	LDL.C	Myocardial infarction	-0.08501	0.01207	-0.013664	0.01264	FAM151A	LOC645436	C1orf175
427	rs17395160	1	55085141	LDL.C	Large vessel disease	-0.08501	0.01207	-0.1123	0.0423	FAM151A	LOC645436	C1orf175
428	rs17395160	1	55085141	LDL.C	Small vessel disease	-0.08501	0.01207	-0.053	0.0447	FAM151A	LOC645436	C1orf175
429	rs17395160	1	55085141	LDL.C	Cardioembolic stroke	-0.08501	0.01207	-0.0181	0.0396	FAM151A	LOC645436	C1orf175

430	rs17395160	1	55085141	LDL.C	Ischemic stroke	-0.08501	0.01207	-0.0578	0.0207	FAM151A	LOC645436	C1orf175
431	rs185415345	1	56625395	LDL.C	Myocardial infarction	-0.16811	0.02727	-0.0279401	0.04292			
432	rs185415345	1	56625395	LDL.C	Coronary heart disease	-0.16811	0.02727	-0.016587	0.037988			
433	rs193084249	1	26987646	LDL.C	Coronary heart disease	0.180124	0.03124	0.06489	0.040959			
434	rs193084249	1	26987646	LDL.C	Myocardial infarction	0.180124	0.03124	0.0372222	0.045195			
435	rs1962352	19	11236180	LDL.C	Small vessel disease	0.065621	0.00993	0.0852	0.0373	LDLR	SMARCA4	SPC24
436	rs1962352	19	11236180	LDL.C	Ischemic stroke	0.065621	0.00993	0.054	0.017	LDLR	SMARCA4	SPC24
437	rs1962352	19	11236180	LDL.C	Large vessel disease	0.065621	0.00993	0.0806	0.0363	LDLR	SMARCA4	SPC24
438	rs1962352	19	11226543	LDL.C	Coronary heart disease	0.065621	0.00993	0.035179	0.009641	LDLR	SMARCA4	SPC24
439	rs1962352	19	11226543	LDL.C	Myocardial infarction	0.065621	0.00993	0.0450183	0.010589	LDLR	SMARCA4	SPC24
440	rs1962352	19	11236180	LDL.C	Cardioembolic stroke	0.065621	0.00993	0.0327	0.0342	LDLR	SMARCA4	SPC24
441	rs207177	1	55790861	LDL.C	Small vessel disease	0.132501	0.01706	-0.0774	0.0599		LOC645506	GOT2L1
442	rs207177	1	55790861	LDL.C	Large vessel disease	0.132501	0.01706	-0.0321	0.0575		LOC645506	GOT2L1
443	rs207177	1	55790861	LDL.C	Ischemic stroke	0.132501	0.01706	0.001	0.0271		LOC645506	GOT2L1
444	rs207177	1	55790861	LDL.C	Cardioembolic stroke	0.132501	0.01706	-0.025	0.0537		LOC645506	GOT2L1
445	rs207177	1	55790336	LDL.C	Myocardial infarction	0.132501	0.01706	-0.00682553	0.019207		LOC645506	GOT2L1
446	rs207177	1	55790336	LDL.C	Coronary heart disease	0.132501	0.01706	-0.006045	0.017737		LOC645506	GOT2L1
447	rs2129944	19	10516198	LDL.C	Coronary heart disease	-0.06425	0.01156	-0.009696	0.010861		CDC37	PDE4A
448	rs2129944	19	10516198	LDL.C	Myocardial infarction	-0.06425	0.01156	-0.0179499	0.012113		CDC37	PDE4A
449	rs2207132	20	39142516	LDL.C	Coronary heart disease	0.139733	0.02452	0.136203	0.03514		HSPEP1	MAFB
450	rs2207132	20	39142516	LDL.C	Myocardial infarction	0.139733	0.02452	0.111906	0.040866		HSPEP1	MAFB
451	rs2207132	20	39179822	LDL.C	Cardioembolic stroke	0.139733	0.02452	0.178	0.205		HSPEP1	MAFB
452	rs56028521	2	21074998	LDL.C	Coronary heart disease	-0.11864	0.01542	-0.015153	0.017241		C2orf43	APOB
453	rs56028521	2	21074998	LDL.C	Myocardial infarction	-0.11864	0.01542	0.0159716	0.019108		C2orf43	APOB
454	rs565436	1	55524601	LDL.C	Myocardial infarction	0.075169	0.01159	0.012141	0.012084	PCSK9	BSND	USP24
455	rs565436	1	55524842	LDL.C	Ischemic stroke	0.075169	0.01159	-0.0035	0.0177	PCSK9	BSND	USP24
456	rs565436	1	55524842	LDL.C	Cardioembolic stroke	0.075169	0.01159	0.0287	0.0349	PCSK9	BSND	USP24
457	rs565436	1	55524842	LDL.C	Large vessel disease	0.075169	0.01159	-0.0286	0.0376	PCSK9	BSND	USP24
458	rs565436	1	55524601	LDL.C	Coronary heart disease	0.075169	0.01159	0.010546	0.010958	PCSK9	BSND	USP24
459	rs565436	1	55524842	LDL.C	Small vessel disease	0.075169	0.01159	-0.0159	0.0394	PCSK9	BSND	USP24
460	rs62523994	8	145031265	LDL.C	Ischemic stroke	0.057408	0.01014	0.0021	0.0165	PLEC1 PLEC1	EPPK1	PARP10
461	rs62523994	8	145026582	LDL.C	Coronary heart disease	0.057408	0.01014	-0.003448	0.01003	PLEC1 PLEC1	EPPK1	PARP10
462	rs62523994	8	145031265	LDL.C	Large vessel disease	0.057408	0.01014	0.0228	0.0353	PLEC1 PLEC1	EPPK1	PARP10
463	rs62523994	8	145026582	LDL.C	Myocardial infarction	0.057408	0.01014	-0.00505999	0.011087	PLEC1 PLEC1	EPPK1	PARP10
464	rs62523994	8	145031265	LDL.C	Small vessel disease	0.057408	0.01014	-0.0497	0.0362	PLEC1 PLEC1	EPPK1	PARP10
465	rs62523994	8	145031265	LDL.C	Cardioembolic stroke	0.057408	0.01014	0.0001	0.0325	PLEC1 PLEC1	EPPK1	PARP10
466	rs629301	1	109818306	LDL.C	Small vessel disease	0.127323	0.0119	-0.0002	0.0422	CELSR2	SARS	PSRC1
467	rs629301	1	109818306	LDL.C	Ischemic stroke	0.127323	0.0119	0.0022	0.0191	CELSR2	SARS	PSRC1
468	rs629301	1	109818306	LDL.C	Coronary heart disease	0.127323	0.0119	0.101444	0.011423	CELSR2	SARS	PSRC1
469	rs629301	1	109818306	LDL.C	Large vessel disease	0.127323	0.0119	0.1	0.0414	CELSR2	SARS	PSRC1
470	rs629301	1	109818306	LDL.C	Myocardial infarction	0.127323	0.0119	0.0875246	0.012685	CELSR2	SARS	PSRC1

471	rs629301	1	109818306	LDL.C	Cardioembolic stroke	0.127323	0.0119	0.0349	0.0376	CELSR2	SARS	PSRC1
472	rs635634	9	136155000	LDL.C	Coronary heart disease	0.069575	0.01235	0.077157	0.011712		ABO	LOC653163
473	rs635634	9	136155000	LDL.C	Myocardial infarction	0.069575	0.01235	0.10933	0.013093		ABO	LOC653163
474	rs6756629	2	44065090	LDL.C	Small vessel disease	-0.14254	0.01817	-0.0699	0.0738	ABCG5<br>ABCG8	DYNC2LI1	ABCG8
475	rs6756629	2	44065090	LDL.C	Myocardial infarction	-0.14254	0.01817	-0.0798551	0.023391	ABCG5<br>ABCG8	DYNC2LI1	ABCG8
476	rs6756629	2	44065090	LDL.C	Coronary heart disease	-0.14254	0.01817	-0.098614	0.020976	ABCG5<br>ABCG8	DYNC2LI1	ABCG8
477	rs6756629	2	44065090	LDL.C	Cardioembolic stroke	-0.14254	0.01817	-0.1936	0.069	ABCG5<br>ABCG8	DYNC2LI1	ABCG8
478	rs6756629	2	44065090	LDL.C	Large vessel disease	-0.14254	0.01817	-0.1785	0.074	ABCG5<br>ABCG8	DYNC2LI1	ABCG8
479	rs6756629	2	44065090	LDL.C	Ischemic stroke	-0.14254	0.01817	-0.1013	0.0335	ABCG5<br>ABCG8	DYNC2LI1	ABCG8
480	rs7256200	19	45415935	LDL.C	Myocardial infarction	0.215084	0.01414	0.0922879	0.017993	APOC1	APOE	APOC1
481	rs7256200	19	45415935	LDL.C	Coronary heart disease	0.215084	0.01414	0.09126	0.016203	APOC1	APOE	APOC1
482	rs73066442	7	21592973	LDL.C	Myocardial infarction	0.071078	0.01143	0.0134498	0.013185			
483	rs73066442	7	21600902	LDL.C	Cardioembolic stroke	0.071078	0.01143	-0.0133	0.0389			
484	rs73066442	7	21600902	LDL.C	Ischemic stroke	0.071078	0.01143	-0.0092	0.0195			
485	rs73066442	7	21592973	LDL.C	Coronary heart disease	0.071078	0.01143	0.017463	0.011775			
486	rs73066442	7	21600902	LDL.C	Large vessel disease	0.071078	0.01143	-0.0617	0.0421			
487	rs73066442	7	21600902	LDL.C	Small vessel disease	0.071078	0.01143	0.0198	0.0423			
488	rs73107473	7	44577622	LDL.C	Coronary heart disease	0.077046	0.01342	0.025694	0.015067			
489	rs73107473	7	44577622	LDL.C	Myocardial infarction	0.077046	0.01342	0.0136987	0.016804			
490	rs7412	19	45412079	LDL.C	Myocardial infarction	-0.59188	0.02506	-0.122218	0.023946	APOE<br>LOC100129500<br>LOC100129500	TOMM40	APOC1
491	rs7412	19	45412079	LDL.C	Coronary heart disease	-0.59188	0.02506	-0.137045	0.021092	APOE<br>LOC100129500<br>LOC100129500	TOMM40	APOC1
492	rs76670936	19	45196581	LDL.C	Myocardial infarction	-0.15027	0.01847	-0.0194139	0.018375			
493	rs76670936	19	45196581	LDL.C	Coronary heart disease	-0.15027	0.01847	-0.019526	0.016483			
494	rs8106814	19	45447161	LDL.C	Small vessel disease	-0.10275	0.01439	0.0264	0.0389		LOC100129500	APOC4
495	rs8106814	19	45447161	LDL.C	Ischemic stroke	-0.10275	0.01439	0.0147	0.0175		LOC100129500	APOC4
496	rs8106814	19	45447161	LDL.C	Large vessel disease	-0.10275	0.01439	-0.0053	0.0379		LOC100129500	APOC4
497	rs8106814	19	45441608	LDL.C	Myocardial infarction	-0.10275	0.01439	-0.0212562	0.015495		LOC100129500	APOC4
498	rs8106814	19	45447161	LDL.C	Cardioembolic stroke	-0.10275	0.01439	0.0207	0.0345		LOC100129500	APOC4
499	rs8106814	19	45441608	LDL.C	Coronary heart disease	-0.10275	0.01439	-0.006333	0.013953		LOC100129500	APOC4
500	rs934197	2	21267461	LDL.C	Myocardial infarction	0.119953	0.01096	0.0403677	0.011725		APOB	LOC100129278
501	rs934197	2	21267461	LDL.C	Coronary heart disease	0.119953	0.01096	0.043338	0.010625		APOB	LOC100129278
502	rs964184	11	116648917	LDL.C	Cardioembolic stroke	-0.08761	0.01402	-0.0127	0.0464	ZNF259	BUD13	ZNF259
503	rs964184	11	116648917	LDL.C	Myocardial infarction	-0.08761	0.01402	-0.0487708	0.013873	ZNF259	BUD13	ZNF259

504	rs964184	11	116648917	LDL.C	Small vessel disease	-0.08761	0.01402	-0.0253	0.0533	ZNF259	BUD13	ZNF259
505	rs964184	11	116648917	LDL.C	Large vessel disease	-0.08761	0.01402	-0.0184	0.0505	ZNF259	BUD13	ZNF259
506	rs964184	11	116648917	LDL.C	Ischemic stroke	-0.08761	0.01402	-0.0074	0.024	ZNF259	BUD13	ZNF259
507	rs964184	11	116648917	LDL.C	Coronary heart disease	-0.08761	0.01402	-0.049958	0.012399	ZNF259	BUD13	ZNF259
508	rs1168001	1	62943954	M.VLDL.TG	Ischemic stroke	0.071966	0.01071	-0.0191	0.0169	DOCK7	USP1	ANGPTL3
509	rs1168001	1	62933758	M.VLDL.TG	Coronary heart disease	0.071966	0.01071	0.011256	0.009631	DOCK7	USP1	ANGPTL3
510	rs1168001	1	62943954	M.VLDL.TG	Small vessel disease	0.071966	0.01071	-0.0881	0.0369	DOCK7	USP1	ANGPTL3
511	rs1168001	1	62933758	M.VLDL.TG	Myocardial infarction	0.071966	0.01071	0.00130512	0.010626	DOCK7	USP1	ANGPTL3
512	rs1168001	1	62943954	M.VLDL.TG	Large vessel disease	0.071966	0.01071	-0.0135	0.0357	DOCK7	USP1	ANGPTL3
513	rs1168001	1	62943954	M.VLDL.TG	Cardioembolic stroke	0.071966	0.01071	-0.0324	0.0332	DOCK7	USP1	ANGPTL3
514	rs116843064	19	8429323	M.VLDL.TG	Coronary heart disease	-0.21024	0.0353	-0.140783	0.042934			
515	rs116843064	19	8429323	M.VLDL.TG	Myocardial infarction	-0.21024	0.0353	-0.0785914	0.047735			
516	rs1260326	2	27730940	M.VLDL.TG	Ischemic stroke	-0.09462	0.01028	0.012	0.0162	GCKR	FNDC4	LOC100130981
517	rs1260326	2	27730940	M.VLDL.TG	Coronary heart disease	-0.09462	0.01028	0.003257	0.00962	GCKR	FNDC4	LOC100130981
518	rs1260326	2	27730940	M.VLDL.TG	Small vessel disease	-0.09462	0.01028	-0.0441	0.0353	GCKR	FNDC4	LOC100130981
519	rs1260326	2	27730940	M.VLDL.TG	Myocardial infarction	-0.09462	0.01028	0.00111651	0.010666	GCKR	FNDC4	LOC100130981
520	rs1260326	2	27730940	M.VLDL.TG	Large vessel disease	-0.09462	0.01028	0.0392	0.0341	GCKR	FNDC4	LOC100130981
521	rs1260326	2	27730940	M.VLDL.TG	Cardioembolic stroke	-0.09462	0.01028	0.0401	0.0314	GCKR	FNDC4	LOC100130981
522	rs34121855	7	73040814	M.VLDL.TG	Coronary heart disease	-0.11673	0.01322	-0.008264	0.012812		MLXIPL	VPS37D
523	rs34121855	7	73040814	M.VLDL.TG	Myocardial infarction	-0.11673	0.01322	-0.0057277	0.01437		MLXIPL	VPS37D
524	rs439401	19	45414451	M.VLDL.TG	Myocardial infarction	0.079744	0.01113	0.0073697	0.011585	LOC100129500	APOE	APOC1
525	rs439401	19	45414451	M.VLDL.TG	Ischemic stroke	0.079744	0.01113	0.0117	0.0185	LOC100129500	APOE	APOC1
526	rs439401	19	45414451	M.VLDL.TG	Large vessel disease	0.079744	0.01113	0.0074	0.0374	LOC100129500	APOE	APOC1
527	rs439401	19	45414451	M.VLDL.TG	Small vessel disease	0.079744	0.01113	0.0657	0.039	LOC100129500	APOE	APOC1
528	rs439401	19	45414451	M.VLDL.TG	Coronary heart disease	0.079744	0.01113	-0.002693	0.010339	LOC100129500	APOE	APOC1
529	rs439401	19	45414451	M.VLDL.TG	Cardioembolic stroke	0.079744	0.01113	-0.039	0.0357	LOC100129500	APOE	APOC1
530	rs6065904	20	44534651	M.VLDL.TG	Myocardial infarction	0.076963	0.01178	-0.0231151	0.01205	PLTP	CTSA	FLJ40606
531	rs6065904	20	44534651	M.VLDL.TG	Ischemic stroke	0.076963	0.01178	-0.0149	0.0197	PLTP	CTSA	FLJ40606
532	rs6065904	20	44534651	M.VLDL.TG	Small vessel disease	0.076963	0.01178	0.039	0.0432	PLTP	CTSA	FLJ40606
533	rs6065904	20	44534651	M.VLDL.TG	Cardioembolic stroke	0.076963	0.01178	-0.005	0.0387	PLTP	CTSA	FLJ40606
534	rs6065904	20	44534651	M.VLDL.TG	Large vessel disease	0.076963	0.01178	-0.0002	0.042	PLTP	CTSA	FLJ40606
535	rs6065904	20	44534651	M.VLDL.TG	Coronary heart disease	0.076963	0.01178	-0.019307	0.010831	PLTP	CTSA	FLJ40606
536	rs673548	2	21237544	M.VLDL.TG	Small vessel disease	-0.0795	0.01125	0.0267	0.0422	APOB	C2orf43	LOC100129278
537	rs673548	2	21237544	M.VLDL.TG	Myocardial infarction	-0.0795	0.01125	-0.00869972	0.011657	APOB	C2orf43	LOC100129278
538	rs673548	2	21237544	M.VLDL.TG	Ischemic stroke	-0.0795	0.01125	0.0108	0.019	APOB	C2orf43	LOC100129278
539	rs673548	2	21237544	M.VLDL.TG	Large vessel disease	-0.0795	0.01125	0.0047	0.0411	APOB	C2orf43	LOC100129278
540	rs673548	2	21237544	M.VLDL.TG	Cardioembolic stroke	-0.0795	0.01125	0.0189	0.0372	APOB	C2orf43	LOC100129278
541	rs673548	2	21237544	M.VLDL.TG	Coronary heart disease	-0.0795	0.01125	0.004686	0.010499	APOB	C2orf43	LOC100129278
542	rs72999033	19	19407718	M.VLDL.TG	Large vessel disease	-0.13825	0.02116	0.1255	0.0702			
543	rs72999033	19	19366632	M.VLDL.TG	Coronary heart disease	-0.13825	0.02116	-0.051959	0.021377			
544	rs72999033	19	19407718	M.VLDL.TG	Cardioembolic stroke	-0.13825	0.02116	0.0972	0.0645			

545	rs72999033	19	19366632	M.VLDL.TG	Myocardial infarction	-0.13825	0.02116	-0.0370382	0.023873			
546	rs72999033	19	19407718	M.VLDL.TG	Small vessel disease	-0.13825	0.02116	0.0398	0.0744			
547	rs72999033	19	19407718	M.VLDL.TG	Ischemic stroke	-0.13825	0.02116	0.0212	0.0335			
548	rs77697917	17	41840849	M.VLDL.TG	Myocardial infarction	0.208758	0.03728	0.100482	0.046662			
549	rs77697917	17	41840849	M.VLDL.TG	Coronary heart disease	0.208758	0.03728	0.086417	0.042583			
550	rs79236614	8	19863471	M.VLDL.TG	Large vessel disease	-0.16649	0.01734	0.0137	0.0575			
551	rs79236614	8	19860460	M.VLDL.TG	Coronary heart disease	-0.16649	0.01734	-0.048868	0.016017			
552	rs79236614	8	19863471	M.VLDL.TG	Ischemic stroke	-0.16649	0.01734	0.0356	0.0277			
553	rs79236614	8	19863471	M.VLDL.TG	Small vessel disease	-0.16649	0.01734	0.0002	0.0624			
554	rs79236614	8	19860460	M.VLDL.TG	Myocardial infarction	-0.16649	0.01734	-0.0613922	0.017899			
555	rs79236614	8	19863471	M.VLDL.TG	Cardioembolic stroke	-0.16649	0.01734	0.093	0.0542			
556	rs821840	16	56993886	M.VLDL.TG	Myocardial infarction	-0.06991	0.01173	-0.0346335	0.012304	CETP	HERPUD1	CETP
557	rs821840	16	56993886	M.VLDL.TG	Coronary heart disease	-0.06991	0.01173	-0.040648	0.011129	CETP	HERPUD1	CETP
558	rs9472125	6	43756169	M.VLDL.TG	Coronary heart disease	-0.0913	0.01627	-0.054856	0.019233		VEGFA	LOC100132354
559	rs9472125	6	43756169	M.VLDL.TG	Myocardial infarction	-0.0913	0.01627	-0.0503078	0.021507		VEGFA	LOC100132354
560	rs964184	11	116648917	M.VLDL.TG	Cardioembolic stroke	-0.22829	0.01407	-0.0127	0.0464	ZNF259	BUD13	ZNF259
561	rs964184	11	116648917	M.VLDL.TG	Ischemic stroke	-0.22829	0.01407	-0.0074	0.024	ZNF259	BUD13	ZNF259
562	rs964184	11	116648917	M.VLDL.TG	Small vessel disease	-0.22829	0.01407	-0.0253	0.0533	ZNF259	BUD13	ZNF259
563	rs964184	11	116648917	M.VLDL.TG	Coronary heart disease	-0.22829	0.01407	-0.049958	0.012399	ZNF259	BUD13	ZNF259
564	rs964184	11	116648917	M.VLDL.TG	Large vessel disease	-0.22829	0.01407	-0.0184	0.0505	ZNF259	BUD13	ZNF259
565	rs964184	11	116648917	M.VLDL.TG	Myocardial infarction	-0.22829	0.01407	-0.0487708	0.013873	ZNF259	BUD13	ZNF259
566	rs1260326	2	27730940	S.HDL.TG	Coronary heart disease	-0.06854	0.01024	0.003257	0.00962	GCKR	FNDC4	LOC100130981
567	rs1260326	2	27730940	S.HDL.TG	Small vessel disease	-0.06854	0.01024	-0.0441	0.0353	GCKR	FNDC4	LOC100130981
568	rs1260326	2	27730940	S.HDL.TG	Ischemic stroke	-0.06854	0.01024	0.012	0.0162	GCKR	FNDC4	LOC100130981
569	rs1260326	2	27730940	S.HDL.TG	Cardioembolic stroke	-0.06854	0.01024	0.0401	0.0314	GCKR	FNDC4	LOC100130981
570	rs1260326	2	27730940	S.HDL.TG	Myocardial infarction	-0.06854	0.01024	0.00111651	0.010666	GCKR	FNDC4	LOC100130981
571	rs1260326	2	27730940	S.HDL.TG	Large vessel disease	-0.06854	0.01024	0.0392	0.0341	GCKR	FNDC4	LOC100130981
572	rs1848922	2	21471603	S.HDL.TG	Myocardial infarction	0.07423	0.01213	0.0301768	0.013003		LOC100129278	LOC645949
573	rs1848922	2	21471603	S.HDL.TG	Coronary heart disease	0.07423	0.01213	0.047369	0.011832		LOC100129278	LOC645949
574	rs34356624	8	19903935	S.HDL.TG	Myocardial infarction	0.22161	0.0392	0.031392	0.051351		LPL	SLC18A1
575	rs34356624	8	19903935	S.HDL.TG	Coronary heart disease	0.22161	0.0392	0.067869	0.04467		LPL	SLC18A1
576	rs3764261	16	56993324	S.HDL.TG	Ischemic stroke	-0.14805	0.01076	0.0102	0.0175		HERPUD1	CETP
577	rs3764261	16	56993324	S.HDL.TG	Coronary heart disease	-0.14805	0.01076	-0.032189	0.010108		HERPUD1	CETP
578	rs3764261	16	56993324	S.HDL.TG	Small vessel disease	-0.14805	0.01076	0.0027	0.0382		HERPUD1	CETP
579	rs3764261	16	56993324	S.HDL.TG	Large vessel disease	-0.14805	0.01076	0.0256	0.0369		HERPUD1	CETP
580	rs3764261	16	56993324	S.HDL.TG	Myocardial infarction	-0.14805	0.01076	-0.0282979	0.011199		HERPUD1	CETP
581	rs3764261	16	56993324	S.HDL.TG	Cardioembolic stroke	-0.14805	0.01076	0.0074	0.0345		HERPUD1	CETP
582	rs429358	19	45411941	S.HDL.TG	Coronary heart disease	0.100423	0.01344	0.090851	0.015181	LOC100129500 APOE APOE	TOMM40	APOC1
583	rs429358	19	45411941	S.HDL.TG	Myocardial infarction	0.100423	0.01344	0.0961904	0.016943	LOC100129500 APOE APOE	TOMM40	APOC1

584	rs4296389	2	21142994	S.HDL.TG	Myocardial infarction	-0.07718	0.01057	-0.0112562	0.01124		C2orf43	APOB
585	rs4296389	2	21142994	S.HDL.TG	Coronary heart disease	-0.07718	0.01057	-0.012159	0.010207		C2orf43	APOB
586	rs5880	16	57015091	S.HDL.TG	Myocardial infarction	0.185425	0.03162	0.0247342	0.0245	CETP	HERPUD1	LOC100130044
587	rs5880	16	57015091	S.HDL.TG	Small vessel disease	0.185425	0.03162	-0.0283	0.0961	CETP	HERPUD1	LOC100130044
588	rs5880	16	57015091	S.HDL.TG	Coronary heart disease	0.185425	0.03162	0.007277	0.022421	CETP	HERPUD1	LOC100130044
589	rs5880	16	57015091	S.HDL.TG	Large vessel disease	0.185425	0.03162	-0.0992	0.0884	CETP	HERPUD1	LOC100130044
590	rs5880	16	57015091	S.HDL.TG	Ischemic stroke	0.185425	0.03162	0.0364	0.0422	CETP	HERPUD1	LOC100130044
591	rs5880	16	57015091	S.HDL.TG	Cardioembolic stroke	0.185425	0.03162	0.0874	0.0838	CETP	HERPUD1	LOC100130044
592	rs6065904	20	44534651	S.HDL.TG	Large vessel disease	0.09016	0.01172	-0.0002	0.042	PLTP	CTSA	FLJ40606
593	rs6065904	20	44534651	S.HDL.TG	Myocardial infarction	0.09016	0.01172	-0.0231151	0.01205	PLTP	CTSA	FLJ40606
594	rs6065904	20	44534651	S.HDL.TG	Ischemic stroke	0.09016	0.01172	-0.0149	0.0197	PLTP	CTSA	FLJ40606
595	rs6065904	20	44534651	S.HDL.TG	Coronary heart disease	0.09016	0.01172	-0.019307	0.010831	PLTP	CTSA	FLJ40606
596	rs6065904	20	44534651	S.HDL.TG	Small vessel disease	0.09016	0.01172	0.039	0.0432	PLTP	CTSA	FLJ40606
597	rs6065904	20	44534651	S.HDL.TG	Cardioembolic stroke	0.09016	0.01172	-0.005	0.0387	PLTP	CTSA	FLJ40606
598	rs6511720	19	11202306	S.HDL.TG	Myocardial infarction	-0.09761	0.01668	-0.100786	0.01894	LDLR	SMARCA4	SPC24
599	rs6511720	19	11202306	S.HDL.TG	Coronary heart disease	-0.09761	0.01668	-0.125298	0.016945	LDLR	SMARCA4	SPC24
600	rs6511720	19	11202306	S.HDL.TG	Cardioembolic stroke	-0.09761	0.01668	-0.0567	0.048	LDLR	SMARCA4	SPC24
601	rs6511720	19	11202306	S.HDL.TG	Small vessel disease	-0.09761	0.01668	-0.013	0.0549	LDLR	SMARCA4	SPC24
602	rs6511720	19	11202306	S.HDL.TG	Large vessel disease	-0.09761	0.01668	-0.0924	0.0525	LDLR	SMARCA4	SPC24
603	rs6511720	19	11202306	S.HDL.TG	Ischemic stroke	-0.09761	0.01668	-0.0708	0.0249	LDLR	SMARCA4	SPC24
604	rs6957745	7	73056750	S.HDL.TG	Myocardial infarction	-0.08997	0.01332	0.00089487	0.0143		MLXIPL	VPS37D
605	rs6957745	7	73056750	S.HDL.TG	Coronary heart disease	-0.08997	0.01332	-0.002156	0.012657		MLXIPL	VPS37D
606	rs79236614	8	19863471	S.HDL.TG	Cardioembolic stroke	-0.16453	0.01727	0.093	0.0542			
607	rs79236614	8	19860460	S.HDL.TG	Myocardial infarction	-0.16453	0.01727	-0.0613922	0.017899			
608	rs79236614	8	19863471	S.HDL.TG	Ischemic stroke	-0.16453	0.01727	0.0356	0.0277			
609	rs79236614	8	19863471	S.HDL.TG	Large vessel disease	-0.16453	0.01727	0.0137	0.0575			
610	rs79236614	8	19860460	S.HDL.TG	Coronary heart disease	-0.16453	0.01727	-0.048868	0.016017			
611	rs79236614	8	19863471	S.HDL.TG	Small vessel disease	-0.16453	0.01727	0.0002	0.0624			
612	rs9472125	6	43756169	S.HDL.TG	Coronary heart disease	-0.09179	0.01615	-0.054856	0.019233		VEGFA	LOC100132354
613	rs9472125	6	43756169	S.HDL.TG	Myocardial infarction	-0.09179	0.01615	-0.0503078	0.021507		VEGFA	LOC100132354
614	rs964184	11	116648917	S.HDL.TG	Large vessel disease	-0.19875	0.01403	-0.0184	0.0505	ZNF259	BUD13	ZNF259
615	rs964184	11	116648917	S.HDL.TG	Small vessel disease	-0.19875	0.01403	-0.0253	0.0533	ZNF259	BUD13	ZNF259
616	rs964184	11	116648917	S.HDL.TG	Cardioembolic stroke	-0.19875	0.01403	-0.0127	0.0464	ZNF259	BUD13	ZNF259
617	rs964184	11	116648917	S.HDL.TG	Ischemic stroke	-0.19875	0.01403	-0.0074	0.024	ZNF259	BUD13	ZNF259
618	rs964184	11	116648917	S.HDL.TG	Myocardial infarction	-0.19875	0.01403	-0.0487708	0.013873	ZNF259	BUD13	ZNF259
619	rs964184	11	116648917	S.HDL.TG	Coronary heart disease	-0.19875	0.01403	-0.049958	0.012399	ZNF259	BUD13	ZNF259
620	rs10401845	19	11195030	S.VLDL.TG	Large vessel disease	-0.07756	0.01311	-0.0754	0.0454		SMARCA4	LDLR
621	rs10401845	19	11191536	S.VLDL.TG	Myocardial infarction	-0.07756	0.01311	-0.0545598	0.014377		SMARCA4	LDLR
622	rs10401845	19	11195030	S.VLDL.TG	Small vessel disease	-0.07756	0.01311	0.0049	0.0464		SMARCA4	LDLR
623	rs10401845	19	11195030	S.VLDL.TG	Cardioembolic stroke	-0.07756	0.01311	-0.0289	0.0421		SMARCA4	LDLR
624	rs10401845	19	11191536	S.VLDL.TG	Coronary heart disease	-0.07756	0.01311	-0.068144	0.012854		SMARCA4	LDLR

625	rs10401845	19	11195030	S.VLDL.TG	Ischemic stroke	-0.07756	0.01311	-0.0645	0.0213		SMARCA4	LDLR
626	rs1042034	2	21225281	S.VLDL.TG	Cardioembolic stroke	0.105215	0.01116	-0.0065	0.037	APOB	C2orf43	LOC100129278
627	rs1042034	2	21225281	S.VLDL.TG	Large vessel disease	0.105215	0.01116	0.0053	0.0411	APOB	C2orf43	LOC100129278
628	rs1042034	2	21225281	S.VLDL.TG	Ischemic stroke	0.105215	0.01116	-0.0078	0.0189	APOB	C2orf43	LOC100129278
629	rs1042034	2	21225281	S.VLDL.TG	Myocardial infarction	0.105215	0.01116	0.0144442	0.011604	APOB	C2orf43	LOC100129278
630	rs1042034	2	21225281	S.VLDL.TG	Small vessel disease	0.105215	0.01116	-0.0211	0.0422	APOB	C2orf43	LOC100129278
631	rs1042034	2	21225281	S.VLDL.TG	Coronary heart disease	0.105215	0.01116	0.001246	0.010546	APOB	C2orf43	LOC100129278
632	rs115849089	8	19912370	S.VLDL.TG	Coronary heart disease	-0.17615	0.01665	-0.057989	0.015294			
633	rs115849089	8	19912370	S.VLDL.TG	Myocardial infarction	-0.17615	0.01665	-0.0667741	0.017021			
634	rs1168041	1	62960250	S.VLDL.TG	Ischemic stroke	0.085968	0.01122	-0.0095	0.0178	DOCK7	USP1	ANGPTL3
635	rs1168041	1	62960250	S.VLDL.TG	Myocardial infarction	0.085968	0.01122	0.00067632	0.011148	DOCK7	USP1	ANGPTL3
636	rs1168041	1	62960250	S.VLDL.TG	Large vessel disease	0.085968	0.01122	-0.0152	0.0373	DOCK7	USP1	ANGPTL3
637	rs1168041	1	62960250	S.VLDL.TG	Cardioembolic stroke	0.085968	0.01122	-0.0236	0.0353	DOCK7	USP1	ANGPTL3
638	rs1168041	1	62960250	S.VLDL.TG	Small vessel disease	0.085968	0.01122	-0.0781	0.0389	DOCK7	USP1	ANGPTL3
639	rs1168041	1	62960250	S.VLDL.TG	Coronary heart disease	0.085968	0.01122	0.011014	0.01011	DOCK7	USP1	ANGPTL3
640	rs116843064	19	8429323	S.VLDL.TG	Coronary heart disease	-0.21144	0.03501	-0.140783	0.042934			
641	rs116843064	19	8429323	S.VLDL.TG	Myocardial infarction	-0.21144	0.03501	-0.0785914	0.047735			
642	rs117001569	8	19574920	S.VLDL.TG	Coronary heart disease	-0.23226	0.04118	-0.090808	0.071186			
643	rs1260326	2	27730940	S.VLDL.TG	Ischemic stroke	-0.09945	0.0102	0.012	0.0162	GCKR	FNDC4	LOC100130981
644	rs1260326	2	27730940	S.VLDL.TG	Myocardial infarction	-0.09945	0.0102	0.00111651	0.010666	GCKR	FNDC4	LOC100130981
645	rs1260326	2	27730940	S.VLDL.TG	Coronary heart disease	-0.09945	0.0102	0.003257	0.00962	GCKR	FNDC4	LOC100130981
646	rs1260326	2	27730940	S.VLDL.TG	Cardioembolic stroke	-0.09945	0.0102	0.0401	0.0314	GCKR	FNDC4	LOC100130981
647	rs1260326	2	27730940	S.VLDL.TG	Small vessel disease	-0.09945	0.0102	-0.0441	0.0353	GCKR	FNDC4	LOC100130981
648	rs1260326	2	27730940	S.VLDL.TG	Large vessel disease	-0.09945	0.0102	0.0392	0.0341	GCKR	FNDC4	LOC100130981
649	rs2980853	8	126478350	S.VLDL.TG	Cardioembolic stroke	-0.0722	0.00983	0.014	0.0315		TRIB1	LOC100130231
650	rs2980853	8	126478350	S.VLDL.TG	Large vessel disease	-0.0722	0.00983	-0.0372	0.0342		TRIB1	LOC100130231
651	rs2980853	8	126478350	S.VLDL.TG	Ischemic stroke	-0.0722	0.00983	-0.0183	0.016		TRIB1	LOC100130231
652	rs2980853	8	126478350	S.VLDL.TG	Coronary heart disease	-0.0722	0.00983	-0.04431	0.009307		TRIB1	LOC100130231
653	rs2980853	8	126478350	S.VLDL.TG	Small vessel disease	-0.0722	0.00983	-0.044	0.0351		TRIB1	LOC100130231
654	rs2980853	8	126478350	S.VLDL.TG	Myocardial infarction	-0.0722	0.00983	-0.0457093	0.01039		TRIB1	LOC100130231
655	rs34346326	7	73016181	S.VLDL.TG	Myocardial infarction	-0.11253	0.01438	-0.00956639	0.014338	MLXIPL<br>MLXIPL<br>MLXIPL	TBL2	VPS37D
656	rs34346326	7	73016181	S.VLDL.TG	Coronary heart disease	-0.11253	0.01438	-0.010364	0.012808	MLXIPL<br>MLXIPL<br>MLXIPL	TBL2	VPS37D
657	rs3826688	19	45418961	S.VLDL.TG	Myocardial infarction	0.089897	0.01111	0.00625308	0.011862	LOC100129500	APOE	APOC4
658	rs3826688	19	45418961	S.VLDL.TG	Coronary heart disease	0.089897	0.01111	-0.001932	0.010616	LOC100129500	APOE	APOC4
659	rs5167	19	45448465	S.VLDL.TG	Coronary heart disease	0.058389	0.01048	-0.011396	0.009972	APOC2<br>APOC4	LOC100129500	APOC2
660	rs5167	19	45448465	S.VLDL.TG	Large vessel disease	0.058389	0.01048	-0.0336	0.0367	APOC2<br>APOC4	LOC100129500	APOC2

661	rs5167	19	45448465	S.VLDL.TG	Cardioembolic stroke	0.058389	0.01048	0.0132	0.0337	APOC2 APOC4	LOC100129500	APOC2
662	rs5167	19	45448465	S.VLDL.TG	Myocardial infarction	0.058389	0.01048	0.00215658	0.011172	APOC2 APOC4	LOC100129500	APOC2
663	rs5167	19	45448465	S.VLDL.TG	Ischemic stroke	0.058389	0.01048	-0.0007	0.0172	APOC2 APOC4	LOC100129500	APOC2
664	rs5167	19	45448465	S.VLDL.TG	Small vessel disease	0.058389	0.01048	-0.0064	0.0383	APOC2 APOC4	LOC100129500	APOC2
665	rs6065904	20	44534651	S.VLDL.TG	Cardioembolic stroke	0.075779	0.0117	-0.005	0.0387	PLTP	CTSA	FLJ40606
666	rs6065904	20	44534651	S.VLDL.TG	Ischemic stroke	0.075779	0.0117	-0.0149	0.0197	PLTP	CTSA	FLJ40606
667	rs6065904	20	44534651	S.VLDL.TG	Myocardial infarction	0.075779	0.0117	-0.0231151	0.01205	PLTP	CTSA	FLJ40606
668	rs6065904	20	44534651	S.VLDL.TG	Small vessel disease	0.075779	0.0117	0.039	0.0432	PLTP	CTSA	FLJ40606
669	rs6065904	20	44534651	S.VLDL.TG	Large vessel disease	0.075779	0.0117	-0.0002	0.042	PLTP	CTSA	FLJ40606
670	rs6065904	20	44534651	S.VLDL.TG	Coronary heart disease	0.075779	0.0117	-0.019307	0.010831	PLTP	CTSA	FLJ40606
671	rs72836561	17	41926126	S.VLDL.TG	Myocardial infarction	0.215165	0.03583	0.0776109	0.045358			
672	rs72836561	17	41926126	S.VLDL.TG	Coronary heart disease	0.215165	0.03583	0.0642	0.041047			
673	rs72999033	19	19407718	S.VLDL.TG	Cardioembolic stroke	-0.15488	0.02101	0.0972	0.0645			
674	rs72999033	19	19407718	S.VLDL.TG	Large vessel disease	-0.15488	0.02101	0.1255	0.0702			
675	rs72999033	19	19366632	S.VLDL.TG	Myocardial infarction	-0.15488	0.02101	-0.0370382	0.023873			
676	rs72999033	19	19407718	S.VLDL.TG	Ischemic stroke	-0.15488	0.02101	0.0212	0.0335			
677	rs72999033	19	19407718	S.VLDL.TG	Small vessel disease	-0.15488	0.02101	0.0398	0.0744			
678	rs72999033	19	19366632	S.VLDL.TG	Coronary heart disease	-0.15488	0.02101	-0.051959	0.021377			
679	rs821840	16	56993886	S.VLDL.TG	Coronary heart disease	-0.10861	0.01163	-0.040648	0.011129	CETP	HERPUD1	CETP
680	rs821840	16	56993886	S.VLDL.TG	Myocardial infarction	-0.10861	0.01163	-0.0346335	0.012304	CETP	HERPUD1	CETP
681	rs9472125	6	43756169	S.VLDL.TG	Coronary heart disease	-0.09517	0.01611	-0.054856	0.019233		VEGFA	LOC100132354
682	rs9472125	6	43756169	S.VLDL.TG	Myocardial infarction	-0.09517	0.01611	-0.0503078	0.021507		VEGFA	LOC100132354
683	rs964184	11	116648917	S.VLDL.TG	Large vessel disease	-0.24229	0.01396	-0.0184	0.0505	ZNF259	BUD13	ZNF259
684	rs964184	11	116648917	S.VLDL.TG	Cardioembolic stroke	-0.24229	0.01396	-0.0127	0.0464	ZNF259	BUD13	ZNF259
685	rs964184	11	116648917	S.VLDL.TG	Myocardial infarction	-0.24229	0.01396	-0.0487708	0.013873	ZNF259	BUD13	ZNF259
686	rs964184	11	116648917	S.VLDL.TG	Ischemic stroke	-0.24229	0.01396	-0.0074	0.024	ZNF259	BUD13	ZNF259
687	rs964184	11	116648917	S.VLDL.TG	Coronary heart disease	-0.24229	0.01396	-0.049958	0.012399	ZNF259	BUD13	ZNF259
688	rs964184	11	116648917	S.VLDL.TG	Small vessel disease	-0.24229	0.01396	-0.0253	0.0533	ZNF259	BUD13	ZNF259
689	rs10402112	19	11191677	TC	Myocardial infarction	-0.20061	0.01695	-0.08984	0.018299		SMARCA4	LDLR
690	rs10402112	19	11191677	TC	Coronary heart disease	-0.20061	0.01695	-0.111993	0.016403		SMARCA4	LDLR
691	rs10449300	1	109381904	TC	Myocardial infarction	-0.06057	0.01082	-0.00383047	0.011533	C1orf62	STXBP3	LOC642864
692	rs10449300	1	109381904	TC	Coronary heart disease	-0.06057	0.01082	-0.008912	0.010388	C1orf62	STXBP3	LOC642864
693	rs111617668	2	44055922	TC	Coronary heart disease	-0.10687	0.01858	-0.094204	0.021066			
694	rs111617668	2	44065090	TC	Small vessel disease	-0.10687	0.01858	-0.0699	0.0738			
695	rs111617668	2	44065090	TC	Large vessel disease	-0.10687	0.01858	-0.1785	0.074			
696	rs111617668	2	44065090	TC	Cardioembolic stroke	-0.10687	0.01858	-0.1936	0.069			
697	rs111617668	2	44065090	TC	Ischemic stroke	-0.10687	0.01858	-0.1013	0.0335			
698	rs111617668	2	44055922	TC	Myocardial infarction	-0.10687	0.01858	-0.0684965	0.023417			

699	rs11591147	1	55505647	TC	Myocardial infarction	-0.46021	0.03486	-0.354653	0.068555	PCSK9	BSND	USP24
700	rs11591147	1	55505647	TC	Coronary heart disease	-0.46021	0.03486	-0.256502	0.057259	PCSK9	BSND	USP24
701	rs1168041	1	62960250	TC	Ischemic stroke	0.063879	0.01124	-0.0095	0.0178	DOCK7	USP1	ANGPTL3
702	rs1168041	1	62960250	TC	Small vessel disease	0.063879	0.01124	-0.0781	0.0389	DOCK7	USP1	ANGPTL3
703	rs1168041	1	62960250	TC	Cardioembolic stroke	0.063879	0.01124	-0.0236	0.0353	DOCK7	USP1	ANGPTL3
704	rs1168041	1	62960250	TC	Myocardial infarction	0.063879	0.01124	0.00067632	0.011148	DOCK7	USP1	ANGPTL3
705	rs1168041	1	62960250	TC	Large vessel disease	0.063879	0.01124	-0.0152	0.0373	DOCK7	USP1	ANGPTL3
706	rs1168041	1	62960250	TC	Coronary heart disease	0.063879	0.01124	0.011014	0.01011	DOCK7	USP1	ANGPTL3
707	rs1260326	2	27730940	TC	Ischemic stroke	-0.06805	0.01022	0.012	0.0162	GCKR	FNDC4	LOC100130981
708	rs1260326	2	27730940	TC	Large vessel disease	-0.06805	0.01022	0.0392	0.0341	GCKR	FNDC4	LOC100130981
709	rs1260326	2	27730940	TC	Coronary heart disease	-0.06805	0.01022	0.003257	0.00962	GCKR	FNDC4	LOC100130981
710	rs1260326	2	27730940	TC	Myocardial infarction	-0.06805	0.01022	0.00111651	0.010666	GCKR	FNDC4	LOC100130981
711	rs1260326	2	27730940	TC	Cardioembolic stroke	-0.06805	0.01022	0.0401	0.0314	GCKR	FNDC4	LOC100130981
712	rs1260326	2	27730940	TC	Small vessel disease	-0.06805	0.01022	-0.0441	0.0353	GCKR	FNDC4	LOC100130981
713	rs143341434	1	54759547	TC	Myocardial infarction	-0.24183	0.02848	-0.0393462	0.060482			
714	rs143341434	1	54759547	TC	Coronary heart disease	-0.24183	0.02848	-0.016655	0.053934			
715	rs144064722	4	73406173	TC	Coronary heart disease	0.257328	0.03404	0.028313	0.031588			
716	rs144064722	4	73406173	TC	Myocardial infarction	0.257328	0.03404	0.032534	0.034056			
717	rs1532085	15	58683366	TC	Myocardial infarction	-0.07682	0.00999	-0.00988838	0.010417		LOC441726	LIPC
718	rs1532085	15	58683366	TC	Cardioembolic stroke	-0.07682	0.00999	0.0091	0.0317		LOC441726	LIPC
719	rs1532085	15	58683366	TC	Coronary heart disease	-0.07682	0.00999	-0.01812	0.009354		LOC441726	LIPC
720	rs1532085	15	58683366	TC	Small vessel disease	-0.07682	0.00999	0.0405	0.0359		LOC441726	LIPC
721	rs1532085	15	58683366	TC	Ischemic stroke	-0.07682	0.00999	0.0215	0.0161		LOC441726	LIPC
722	rs1532085	15	58683366	TC	Large vessel disease	-0.07682	0.00999	0.016	0.0343		LOC441726	LIPC
723	rs17699030	19	11330942	TC	Small vessel disease	-0.14729	0.02544	-0.0209	0.1076	DOCK6	KANK2	LOC55908
724	rs17699030	19	11330942	TC	Cardioembolic stroke	-0.14729	0.02544	0.0981	0.0922	DOCK6	KANK2	LOC55908
725	rs17699030	19	11330942	TC	Ischemic stroke	-0.14729	0.02544	0.0162	0.0481	DOCK6	KANK2	LOC55908
726	rs17699030	19	11330942	TC	Large vessel disease	-0.14729	0.02544	-0.0156	0.1066	DOCK6	KANK2	LOC55908
727	rs17699030	19	11330942	TC	Coronary heart disease	-0.14729	0.02544	0.012748	0.023807	DOCK6	KANK2	LOC55908
728	rs17699030	19	11330942	TC	Myocardial infarction	-0.14729	0.02544	-0.0045677	0.026753	DOCK6	KANK2	LOC55908
729	rs185415345	1	56625395	TC	Coronary heart disease	-0.15302	0.02732	-0.016587	0.037988			
730	rs185415345	1	56625395	TC	Myocardial infarction	-0.15302	0.02732	-0.0279401	0.04292			
731	rs207177	1	55790861	TC	Small vessel disease	0.122368	0.01711	-0.0774	0.0599		LOC645506	GOT2L1
732	rs207177	1	55790336	TC	Myocardial infarction	0.122368	0.01711	-0.00682553	0.019207		LOC645506	GOT2L1
733	rs207177	1	55790861	TC	Cardioembolic stroke	0.122368	0.01711	-0.025	0.0537		LOC645506	GOT2L1
734	rs207177	1	55790336	TC	Coronary heart disease	0.122368	0.01711	-0.006045	0.017737		LOC645506	GOT2L1
735	rs207177	1	55790861	TC	Ischemic stroke	0.122368	0.01711	0.001	0.0271		LOC645506	GOT2L1
736	rs207177	1	55790861	TC	Large vessel disease	0.122368	0.01711	-0.0321	0.0575		LOC645506	GOT2L1
737	rs2207132	20	39142516	TC	Myocardial infarction	0.135773	0.02456	0.111906	0.040866		HSPEP1	MAFB
738	rs2207132	20	39142516	TC	Coronary heart disease	0.135773	0.02456	0.136203	0.03514		HSPEP1	MAFB
739	rs2207132	20	39179822	TC	Cardioembolic stroke	0.135773	0.02456	0.178	0.205		HSPEP1	MAFB

740	rs2495477	1	55518467	TC	Myocardial infarction	-0.06096	0.0108	-0.0362507	0.011685	PCSK9	BSND	USP24
741	rs2495477	1	55518467	TC	Coronary heart disease	-0.06096	0.0108	-0.035937	0.010473	PCSK9	BSND	USP24
742	rs261334	15	58726744	TC	Small vessel disease	-0.11089	0.01193	-0.0739	0.0451	LIPC	LOC441726	ADAM10
743	rs261334	15	58726744	TC	Coronary heart disease	-0.11089	0.01193	-0.03756	0.011043	LIPC	LOC441726	ADAM10
744	rs261334	15	58726744	TC	Large vessel disease	-0.11089	0.01193	0.0677	0.0452	LIPC	LOC441726	ADAM10
745	rs261334	15	58726744	TC	Myocardial infarction	-0.11089	0.01193	-0.0402744	0.012395	LIPC	LOC441726	ADAM10
746	rs261334	15	58726744	TC	Ischemic stroke	-0.11089	0.01193	0.0383	0.0211	LIPC	LOC441726	ADAM10
747	rs261334	15	58726744	TC	Cardioembolic stroke	-0.11089	0.01193	0.0623	0.0423	LIPC	LOC441726	ADAM10
748	rs2980853	8	126478350	TC	Ischemic stroke	-0.0625	0.00984	-0.0183	0.016		TRIB1	LOC100130231
749	rs2980853	8	126478350	TC	Small vessel disease	-0.0625	0.00984	-0.044	0.0351		TRIB1	LOC100130231
750	rs2980853	8	126478350	TC	Cardioembolic stroke	-0.0625	0.00984	0.014	0.0315		TRIB1	LOC100130231
751	rs2980853	8	126478350	TC	Coronary heart disease	-0.0625	0.00984	-0.04431	0.009307		TRIB1	LOC100130231
752	rs2980853	8	126478350	TC	Myocardial infarction	-0.0625	0.00984	-0.0457093	0.01039		TRIB1	LOC100130231
753	rs2980853	8	126478350	TC	Large vessel disease	-0.0625	0.00984	-0.0372	0.0342		TRIB1	LOC100130231
754	rs3741298	11	116657561	TC	Coronary heart disease	-0.09192	0.01181	-0.036789	0.010785	ZNF259	BUD13	APOA5
755	rs3741298	11	116657561	TC	Myocardial infarction	-0.09192	0.01181	-0.043033	0.012111	ZNF259	BUD13	APOA5
756	rs61770425	1	55085125	TC	Coronary heart disease	-0.06968	0.01214	-0.013365	0.011281	ACOT11	LOC645436	C1orf175
757	rs61770425	1	55085141	TC	Small vessel disease	-0.06968	0.01214	-0.053	0.0447	ACOT11	LOC645436	C1orf175
758	rs61770425	1	55085125	TC	Myocardial infarction	-0.06968	0.01214	-0.0143256	0.012498	ACOT11	LOC645436	C1orf175
759	rs61770425	1	55085141	TC	Large vessel disease	-0.06968	0.01214	-0.1123	0.0423	ACOT11	LOC645436	C1orf175
760	rs61770425	1	55085141	TC	Cardioembolic stroke	-0.06968	0.01214	-0.0181	0.0396	ACOT11	LOC645436	C1orf175
761	rs61770425	1	55085141	TC	Ischemic stroke	-0.06968	0.01214	-0.0578	0.0207	ACOT11	LOC645436	C1orf175
762	rs629301	1	109818306	TC	Myocardial infarction	0.101805	0.01195	0.0875246	0.012685	CELSR2	SARS	PSRC1
763	rs629301	1	109818306	TC	Small vessel disease	0.101805	0.01195	-0.0002	0.0422	CELSR2	SARS	PSRC1
764	rs629301	1	109818306	TC	Ischemic stroke	0.101805	0.01195	0.0022	0.0191	CELSR2	SARS	PSRC1
765	rs629301	1	109818306	TC	Cardioembolic stroke	0.101805	0.01195	0.0349	0.0376	CELSR2	SARS	PSRC1
766	rs629301	1	109818306	TC	Coronary heart disease	0.101805	0.01195	0.101444	0.011423	CELSR2	SARS	PSRC1
767	rs629301	1	109818306	TC	Large vessel disease	0.101805	0.01195	0.1	0.0414	CELSR2	SARS	PSRC1
768	rs7256200	19	45415935	TC	Myocardial infarction	0.166001	0.01421	0.0922879	0.017993	APOC1	APOE	APOC1
769	rs7256200	19	45415935	TC	Coronary heart disease	0.166001	0.01421	0.09126	0.016203	APOC1	APOE	APOC1
770	rs72999033	19	19407718	TC	Small vessel disease	-0.12612	0.02104	0.0398	0.0744			
771	rs72999033	19	19407718	TC	Large vessel disease	-0.12612	0.02104	0.1255	0.0702			
772	rs72999033	19	19366632	TC	Myocardial infarction	-0.12612	0.02104	-0.0370382	0.023873			
773	rs72999033	19	19407718	TC	Ischemic stroke	-0.12612	0.02104	0.0212	0.0335			
774	rs72999033	19	19407718	TC	Cardioembolic stroke	-0.12612	0.02104	0.0972	0.0645			
775	rs72999033	19	19366632	TC	Coronary heart disease	-0.12612	0.02104	-0.051959	0.021377			
776	rs7412	19	45412079	TC	Myocardial infarction	-0.41162	0.02529	-0.122218	0.023946	APOE<br>LOC100129500<br>LOC100129500	TOMM40	APOC1

777	rs7412	19	45412079	TC	Coronary heart disease	-0.41162	0.02529	-0.137045	0.021092	APOE LOC100129500 LOC100129500	TOMM40	APOC1
778	rs76670936	19	45196581	TC	Myocardial infarction	-0.10818	0.01853	-0.0194139	0.018375			
779	rs76670936	19	45196581	TC	Coronary heart disease	-0.10818	0.01853	-0.019526	0.016483			
780	rs79225634	5	74619639	TC	Myocardial infarction	0.083426	0.01048	0.0202878	0.010617			
781	rs79225634	5	74619639	TC	Coronary heart disease	0.083426	0.01048	0.024313	0.009685			
782	rs952275	2	21221399	TC	Ischemic stroke	0.088994	0.00989	0.0069	0.0171		C2orf43	APOB
783	rs952275	2	21221399	TC	Myocardial infarction	0.088994	0.00989	0.00687294	0.010567		C2orf43	APOB
784	rs952275	2	21221399	TC	Small vessel disease	0.088994	0.00989	0.0264	0.0391		C2orf43	APOB
785	rs952275	2	21221399	TC	Coronary heart disease	0.088994	0.00989	0.016832	0.00964		C2orf43	APOB
786	rs952275	2	21221399	TC	Large vessel disease	0.088994	0.00989	-0.0492	0.0389		C2orf43	APOB
787	rs952275	2	21221399	TC	Cardioembolic stroke	0.088994	0.00989	0.0069	0.0348		C2orf43	APOB
788	rs115849089	8	19912370	TG	Coronary heart disease	-0.16206	0.01666	-0.057989	0.015294			
789	rs115849089	8	19912370	TG	Myocardial infarction	-0.16206	0.01666	-0.0667741	0.017021			
790	rs1168041	1	62960250	TG	Large vessel disease	0.095641	0.01121	-0.0152	0.0373	DOCK7	USP1	ANGPTL3
791	rs1168041	1	62960250	TG	Small vessel disease	0.095641	0.01121	-0.0781	0.0389	DOCK7	USP1	ANGPTL3
792	rs1168041	1	62960250	TG	Coronary heart disease	0.095641	0.01121	0.011014	0.01011	DOCK7	USP1	ANGPTL3
793	rs1168041	1	62960250	TG	Myocardial infarction	0.095641	0.01121	0.00067632	0.011148	DOCK7	USP1	ANGPTL3
794	rs1168041	1	62960250	TG	Cardioembolic stroke	0.095641	0.01121	-0.0236	0.0353	DOCK7	USP1	ANGPTL3
795	rs1168041	1	62960250	TG	Ischemic stroke	0.095641	0.01121	-0.0095	0.0178	DOCK7	USP1	ANGPTL3
796	rs1260326	2	27730940	TG	Coronary heart disease	-0.106	0.01021	0.003257	0.00962	GCKR	FNDC4	LOC100130981
797	rs1260326	2	27730940	TG	Large vessel disease	-0.106	0.01021	0.0392	0.0341	GCKR	FNDC4	LOC100130981
798	rs1260326	2	27730940	TG	Small vessel disease	-0.106	0.01021	-0.0441	0.0353	GCKR	FNDC4	LOC100130981
799	rs1260326	2	27730940	TG	Cardioembolic stroke	-0.106	0.01021	0.0401	0.0314	GCKR	FNDC4	LOC100130981
800	rs1260326	2	27730940	TG	Myocardial infarction	-0.106	0.01021	0.00111651	0.010666	GCKR	FNDC4	LOC100130981
801	rs1260326	2	27730940	TG	Ischemic stroke	-0.106	0.01021	0.012	0.0162	GCKR	FNDC4	LOC100130981
802	rs2980853	8	126478350	TG	Coronary heart disease	-0.0744	0.00982	-0.04431	0.009307		TRIB1	LOC100130231
803	rs2980853	8	126478350	TG	Small vessel disease	-0.0744	0.00982	-0.044	0.0351		TRIB1	LOC100130231
804	rs2980853	8	126478350	TG	Myocardial infarction	-0.0744	0.00982	-0.0457093	0.01039		TRIB1	LOC100130231
805	rs2980853	8	126478350	TG	Cardioembolic stroke	-0.0744	0.00982	0.014	0.0315		TRIB1	LOC100130231
806	rs2980853	8	126478350	TG	Large vessel disease	-0.0744	0.00982	-0.0372	0.0342		TRIB1	LOC100130231
807	rs2980853	8	126478350	TG	Ischemic stroke	-0.0744	0.00982	-0.0183	0.016		TRIB1	LOC100130231
808	rs34346326	7	73016181	TG	Coronary heart disease	-0.09891	0.01306	-0.010364	0.012808	MLXIPL MLXIPL MLXIPL	TBL2	VPS37D
809	rs34346326	7	73016181	TG	Myocardial infarction	-0.09891	0.01306	-0.00956639	0.014338	MLXIPL MLXIPL MLXIPL	TBL2	VPS37D
810	rs429358	19	45411941	TG	Myocardial infarction	0.117062	0.0134	0.0961904	0.016943	LOC100129500 APOE APOE	TOMM40	APOC1

811	rs429358	19	45411941	TG	Coronary heart disease	0.117062	0.0134	0.090851	0.015181	LOC100129500 APOE APOE	TOMM40	APOC1
812	rs4296389	2	21142994	TG	Myocardial infarction	-0.09141	0.01053	-0.0112562	0.01124		C2orf43	APOB
813	rs4296389	2	21142994	TG	Coronary heart disease	-0.09141	0.01053	-0.012159	0.010207		C2orf43	APOB
814	rs5167	19	45448465	TG	Small vessel disease	0.062703	0.01049	-0.0064	0.0383	APOC2 APOC4	LOC100129500	APOC2
815	rs5167	19	45448465	TG	Ischemic stroke	0.062703	0.01049	-0.0007	0.0172	APOC2 APOC4	LOC100129500	APOC2
816	rs5167	19	45448465	TG	Large vessel disease	0.062703	0.01049	-0.0336	0.0367	APOC2 APOC4	LOC100129500	APOC2
817	rs5167	19	45448465	TG	Myocardial infarction	0.062703	0.01049	0.00215658	0.011172	APOC2 APOC4	LOC100129500	APOC2
818	rs5167	19	45448465	TG	Cardioembolic stroke	0.062703	0.01049	0.0132	0.0337	APOC2 APOC4	LOC100129500	APOC2
819	rs5167	19	45448465	TG	Coronary heart disease	0.062703	0.01049	-0.011396	0.009972	APOC2 APOC4	LOC100129500	APOC2
820	rs72999033	19	19366632	TG	Myocardial infarction	-0.16887	0.02101	-0.0370382	0.023873			
821	rs72999033	19	19407718	TG	Small vessel disease	-0.16887	0.02101	0.0398	0.0744			
822	rs72999033	19	19407718	TG	Large vessel disease	-0.16887	0.02101	0.1255	0.0702			
823	rs72999033	19	19407718	TG	Cardioembolic stroke	-0.16887	0.02101	0.0972	0.0645			
824	rs72999033	19	19366632	TG	Coronary heart disease	-0.16887	0.02101	-0.051959	0.021377			
825	rs72999033	19	19407718	TG	Ischemic stroke	-0.16887	0.02101	0.0212	0.0335			
826	rs821840	16	56993886	TG	Myocardial infarction	-0.08153	0.01165	-0.0346335	0.012304	CETP	HERPUD1	CETP
827	rs821840	16	56993886	TG	Coronary heart disease	-0.08153	0.01165	-0.040648	0.011129	CETP	HERPUD1	CETP
828	rs9472125	6	43756169	TG	Coronary heart disease	-0.09456	0.01612	-0.054856	0.019233		VEGFA	LOC100132354
829	rs9472125	6	43756169	TG	Myocardial infarction	-0.09456	0.01612	-0.0503078	0.021507		VEGFA	LOC100132354
830	rs964184	11	116648917	TG	Small vessel disease	-0.23269	0.01397	-0.0253	0.0533	ZNF259	BUD13	ZNF259
831	rs964184	11	116648917	TG	Cardioembolic stroke	-0.23269	0.01397	-0.0127	0.0464	ZNF259	BUD13	ZNF259
832	rs964184	11	116648917	TG	Coronary heart disease	-0.23269	0.01397	-0.049958	0.012399	ZNF259	BUD13	ZNF259
833	rs964184	11	116648917	TG	Myocardial infarction	-0.23269	0.01397	-0.0487708	0.013873	ZNF259	BUD13	ZNF259
834	rs964184	11	116648917	TG	Ischemic stroke	-0.23269	0.01397	-0.0074	0.024	ZNF259	BUD13	ZNF259
835	rs964184	11	116648917	TG	Large vessel disease	-0.23269	0.01397	-0.0184	0.0505	ZNF259	BUD13	ZNF259
836	rs11096689	2	21140540	XL.HDL.TG	Myocardial infarction	-0.07276	0.01112	-0.014713	0.01202		C2orf43	APOB
837	rs11096689	2	21140540	XL.HDL.TG	Cardioembolic stroke	-0.07276	0.01112	-0.0376	0.038		C2orf43	APOB
838	rs11096689	2	21140540	XL.HDL.TG	Ischemic stroke	-0.07276	0.01112	-0.0003	0.0189		C2orf43	APOB
839	rs11096689	2	21140540	XL.HDL.TG	Large vessel disease	-0.07276	0.01112	0.0637	0.0426		C2orf43	APOB
840	rs11096689	2	21140540	XL.HDL.TG	Coronary heart disease	-0.07276	0.01112	-0.022822	0.010891		C2orf43	APOB
841	rs11096689	2	21140540	XL.HDL.TG	Small vessel disease	-0.07276	0.01112	-0.0216	0.043		C2orf43	APOB
842	rs113105798	15	59301460	XL.HDL.TG	Coronary heart disease	0.319035	0.03426	-0.055219	0.040092			
843	rs113105798	15	59301460	XL.HDL.TG	Myocardial infarction	0.319035	0.03426	-0.0400261	0.044667			
844	rs113531395	17	4886829	XL.HDL.TG	Myocardial infarction	-0.20437	0.03621	-0.0573797	0.067105			
845	rs113531395	17	4886829	XL.HDL.TG	Coronary heart disease	-0.20437	0.03621	-0.046539	0.061719			
846	rs11638718	15	58079462	XL.HDL.TG	Cardioembolic stroke	-0.07099	0.01214	0.0083	0.0378		GRINL1A	ALDH1A2

847	rs11638718	15	58079462	XL.HDL.TG	Myocardial infarction	-0.07099	0.01214	-0.0261497	0.012643		GRINL1A	ALDH1A2
848	rs11638718	15	58079462	XL.HDL.TG	Large vessel disease	-0.07099	0.01214	0.0158	0.0405		GRINL1A	ALDH1A2
849	rs11638718	15	58079462	XL.HDL.TG	Coronary heart disease	-0.07099	0.01214	-0.020832	0.011376		GRINL1A	ALDH1A2
850	rs11638718	15	58079462	XL.HDL.TG	Ischemic stroke	-0.07099	0.01214	-0.0051	0.0197		GRINL1A	ALDH1A2
851	rs11638718	15	58079462	XL.HDL.TG	Small vessel disease	-0.07099	0.01214	-0.0337	0.0434		GRINL1A	ALDH1A2
852	rs11662691	18	47183012	XL.HDL.TG	Coronary heart disease	0.094038	0.01612	-0.027382	0.017857		LOC100129143	ACAA2
853	rs11662691	18	47183012	XL.HDL.TG	Cardioembolic stroke	0.094038	0.01612	0.0063	0.0676		LOC100129143	ACAA2
854	rs11662691	18	47183012	XL.HDL.TG	Ischemic stroke	0.094038	0.01612	-0.0278	0.0341		LOC100129143	ACAA2
855	rs11662691	18	47183012	XL.HDL.TG	Large vessel disease	0.094038	0.01612	-0.0217	0.0717		LOC100129143	ACAA2
856	rs11662691	18	47183012	XL.HDL.TG	Small vessel disease	0.094038	0.01612	-0.0299	0.0744		LOC100129143	ACAA2
857	rs11662691	18	47183012	XL.HDL.TG	Myocardial infarction	0.094038	0.01612	-0.0341334	0.019624		LOC100129143	ACAA2
858	rs1318175	15	58586129	XL.HDL.TG	Myocardial infarction	-0.15738	0.01308	-0.029757	0.014056		LOC441726	LIPC
859	rs1318175	15	58586129	XL.HDL.TG	Coronary heart disease	-0.15738	0.01308	-0.021941	0.012657		LOC441726	LIPC
860	rs146842281	15	59356659	XL.HDL.TG	Coronary heart disease	0.255873	0.02182	0.025121	0.033363			
861	rs146842281	15	59356659	XL.HDL.TG	Myocardial infarction	0.255873	0.02182	0.0331942	0.039409			
862	rs149066100	15	58603692	XL.HDL.TG	Coronary heart disease	0.112049	0.01619	0.02181	0.019703			
863	rs149066100	15	58603692	XL.HDL.TG	Myocardial infarction	0.112049	0.01619	0.0361669	0.021983			
864	rs1532085	15	58683366	XL.HDL.TG	Cardioembolic stroke	-0.26429	0.00986	0.0091	0.0317		LOC441726	LIPC
865	rs1532085	15	58683366	XL.HDL.TG	Small vessel disease	-0.26429	0.00986	0.0405	0.0359		LOC441726	LIPC
866	rs1532085	15	58683366	XL.HDL.TG	Large vessel disease	-0.26429	0.00986	0.016	0.0343		LOC441726	LIPC
867	rs1532085	15	58683366	XL.HDL.TG	Ischemic stroke	-0.26429	0.00986	0.0215	0.0161		LOC441726	LIPC
868	rs1532085	15	58683366	XL.HDL.TG	Coronary heart disease	-0.26429	0.00986	-0.01812	0.009354		LOC441726	LIPC
869	rs1532085	15	58683366	XL.HDL.TG	Myocardial infarction	-0.26429	0.00986	-0.00988838	0.010417		LOC441726	LIPC
870	rs1540037	18	47182664	XL.HDL.TG	Myocardial infarction	0.091687	0.01213	-0.00483348	0.013661		LOC100129143	ACAA2
871	rs1540037	18	47182664	XL.HDL.TG	Coronary heart disease	0.091687	0.01213	-0.012746	0.012202		LOC100129143	ACAA2
872	rs17231506	16	56994528	XL.HDL.TG	Coronary heart disease	0.069101	0.01085	-0.033139	0.010231	CETP	HERPUD1	CETP
873	rs17231506	16	56994528	XL.HDL.TG	Myocardial infarction	0.069101	0.01085	-0.0265218	0.011326	CETP	HERPUD1	CETP
874	rs17414716	1	55759138	XL.HDL.TG	Coronary heart disease	-0.21326	0.03256	-0.144297	0.058125		LOC645506	GOT2L1
875	rs17414716	1	55759138	XL.HDL.TG	Myocardial infarction	-0.21326	0.03256	-0.160083	0.065546		LOC645506	GOT2L1
876	rs181835401	1	63135955	XL.HDL.TG	Coronary heart disease	-0.0897	0.01131	-0.01186	0.009972			
877	rs181835401	1	63139730	XL.HDL.TG	Cardioembolic stroke	-0.0897	0.01131	0.0246	0.0347			
878	rs181835401	1	63139730	XL.HDL.TG	Large vessel disease	-0.0897	0.01131	0.0175	0.0369			
879	rs181835401	1	63139730	XL.HDL.TG	Ischemic stroke	-0.0897	0.01131	0.0189	0.0173			
880	rs181835401	1	63135955	XL.HDL.TG	Myocardial infarction	-0.0897	0.01131	-0.00619888	0.01101			
881	rs181835401	1	63139730	XL.HDL.TG	Small vessel disease	-0.0897	0.01131	0.0816	0.0377			
882	rs192924868	15	59231939	XL.HDL.TG	Coronary heart disease	0.115122	0.02055	0.002444	0.025109			
883	rs192924868	15	59231939	XL.HDL.TG	Myocardial infarction	0.115122	0.02055	0.0053657	0.028532			
884	rs2044332	15	58646641	XL.HDL.TG	Coronary heart disease	0.12199	0.01448	0.005484	0.013358		LOC441726	LIPC
885	rs2044332	15	58646641	XL.HDL.TG	Myocardial infarction	0.12199	0.01448	-0.00567182	0.014995		LOC441726	LIPC
886	rs2070895	15	58723939	XL.HDL.TG	Cardioembolic stroke	0.301697	0.01151	-0.0622	0.0419	LIPC	LOC441726	LIPC
887	rs2070895	15	58723939	XL.HDL.TG	Small vessel disease	0.301697	0.01151	0.0715	0.0449	LIPC	LOC441726	LIPC

888	rs2070895	15	58723939	XL.HDL.TG	Ischemic stroke	0.301697	0.01151	-0.0355	0.0209	LIPC	LOC441726	LIPC
889	rs2070895	15	58723939	XL.HDL.TG	Large vessel disease	0.301697	0.01151	-0.0729	0.0446	LIPC	LOC441726	LIPC
890	rs2070895	15	58723939	XL.HDL.TG	Coronary heart disease	0.301697	0.01151	0.037159	0.010782	LIPC	LOC441726	LIPC
891	rs2070895	15	58723939	XL.HDL.TG	Myocardial infarction	0.301697	0.01151	0.0413675	0.012105	LIPC	LOC441726	LIPC
892	rs28595548	15	58464126	XL.HDL.TG	Small vessel disease	-0.086	0.01109	-0.0199	0.0418	AQP9	ALDH1A2	LOC441726
893	rs28595548	15	58464126	XL.HDL.TG	Cardioembolic stroke	-0.086	0.01109	0.0077	0.0377	AQP9	ALDH1A2	LOC441726
894	rs28595548	15	58464126	XL.HDL.TG	Ischemic stroke	-0.086	0.01109	-0.0018	0.0189	AQP9	ALDH1A2	LOC441726
895	rs28595548	15	58457666	XL.HDL.TG	Myocardial infarction	-0.086	0.01109	0.00804728	0.012505	AQP9	ALDH1A2	LOC441726
896	rs28595548	15	58457666	XL.HDL.TG	Coronary heart disease	-0.086	0.01109	0.010437	0.011338	AQP9	ALDH1A2	LOC441726
897	rs28595548	15	58464126	XL.HDL.TG	Large vessel disease	-0.086	0.01109	0.0202	0.0402	AQP9	ALDH1A2	LOC441726
898	rs2881925	2	20390694	XL.HDL.TG	Cardioembolic stroke	-0.05615	0.00978	0.0148	0.0332		LOC402069	SDC1
899	rs2881925	2	20390694	XL.HDL.TG	Ischemic stroke	-0.05615	0.00978	0.0173	0.0166		LOC402069	SDC1
900	rs2881925	2	20390694	XL.HDL.TG	Coronary heart disease	-0.05615	0.00978	0.015701	0.009477		LOC402069	SDC1
901	rs2881925	2	20390694	XL.HDL.TG	Large vessel disease	-0.05615	0.00978	-0.0261	0.0356		LOC402069	SDC1
902	rs2881925	2	20390694	XL.HDL.TG	Small vessel disease	-0.05615	0.00978	0.0435	0.0363		LOC402069	SDC1
903	rs2881925	2	20390694	XL.HDL.TG	Myocardial infarction	-0.05615	0.00978	0.0242637	0.010491		LOC402069	SDC1
904	rs439401	19	45414451	XL.HDL.TG	Cardioembolic stroke	0.086072	0.01103	-0.039	0.0357	LOC100129500	APOE	APOC1
905	rs439401	19	45414451	XL.HDL.TG	Large vessel disease	0.086072	0.01103	0.0074	0.0374	LOC100129500	APOE	APOC1
906	rs439401	19	45414451	XL.HDL.TG	Myocardial infarction	0.086072	0.01103	0.0073697	0.011585	LOC100129500	APOE	APOC1
907	rs439401	19	45414451	XL.HDL.TG	Coronary heart disease	0.086072	0.01103	-0.002693	0.010339	LOC100129500	APOE	APOC1
908	rs439401	19	45414451	XL.HDL.TG	Small vessel disease	0.086072	0.01103	0.0657	0.039	LOC100129500	APOE	APOC1
909	rs439401	19	45414451	XL.HDL.TG	Ischemic stroke	0.086072	0.01103	0.0117	0.0185	LOC100129500	APOE	APOC1
910	rs6073958	20	44551855	XL.HDL.TG	Coronary heart disease	-0.14546	0.01232	-0.025141	0.011769		PLTP	FLJ40606
911	rs6073958	20	44585420	XL.HDL.TG	Large vessel disease	-0.14546	0.01232	0.041	0.0442		PLTP	FLJ40606
912	rs6073958	20	44585420	XL.HDL.TG	Small vessel disease	-0.14546	0.01232	0.0586	0.0454		PLTP	FLJ40606
913	rs6073958	20	44585420	XL.HDL.TG	Cardioembolic stroke	-0.14546	0.01232	0.0147	0.0415		PLTP	FLJ40606
914	rs6073958	20	44585420	XL.HDL.TG	Ischemic stroke	-0.14546	0.01232	0.0042	0.0207		PLTP	FLJ40606
915	rs6073958	20	44551855	XL.HDL.TG	Myocardial infarction	-0.14546	0.01232	-0.0290105	0.012879		PLTP	FLJ40606
916	rs61999891	15	58299599	XL.HDL.TG	Myocardial infarction	0.12184	0.01731	0.00774116	0.016926	ALDH1A2 ALDH1A2	LOC100132719	AQP9
917	rs61999891	15	58299599	XL.HDL.TG	Coronary heart disease	0.12184	0.01731	-0.022129	0.015103	ALDH1A2 ALDH1A2	LOC100132719	AQP9
918	rs72739708	15	57739519	XL.HDL.TG	Cardioembolic stroke	0.113507	0.02057	-0.0147	0.0906			
919	rs72739708	15	57739519	XL.HDL.TG	Large vessel disease	0.113507	0.02057	0.0786	0.0962			
920	rs72739708	15	57739519	XL.HDL.TG	Small vessel disease	0.113507	0.02057	0.1391	0.1067			
921	rs72739708	15	57733779	XL.HDL.TG	Coronary heart disease	0.113507	0.02057	-0.013291	0.024874			
922	rs72739708	15	57739519	XL.HDL.TG	Ischemic stroke	0.113507	0.02057	0.0697	0.0474			
923	rs72739708	15	57733779	XL.HDL.TG	Myocardial infarction	0.113507	0.02057	-0.00974298	0.028442			
924	rs73424577	15	58869185	XL.HDL.TG	Coronary heart disease	0.305039	0.02803	0.043542	0.027498			
925	rs73424577	15	58891114	XL.HDL.TG	Ischemic stroke	0.305039	0.02803	-0.0391	0.0532			
926	rs73424577	15	58891114	XL.HDL.TG	Cardioembolic stroke	0.305039	0.02803	0.0994	0.0994			

927	rs73424577	15	58891114	XL.HDL.TG	Large vessel disease	0.305039	0.02803	-0.0219	0.1133			
928	rs73424577	15	58891114	XL.HDL.TG	Small vessel disease	0.305039	0.02803	0.0052	0.1173			
929	rs73424577	15	58869185	XL.HDL.TG	Myocardial infarction	0.305039	0.02803	0.0282865	0.030666			
930	rs75679663	17	4667972	XL.HDL.TG	Coronary heart disease	-0.22505	0.03865	-0.011031	0.066703			
931	rs75679663	17	4667972	XL.HDL.TG	Myocardial infarction	-0.22505	0.03865	0.0280457	0.073714			
932	rs8100204	19	19393714	XL.HDL.TG	Coronary heart disease	-0.09559	0.01609	-0.006372	0.01473	SF4	TM6SF2	KIAA0892
933	rs8100204	19	19393714	XL.HDL.TG	Myocardial infarction	-0.09559	0.01609	-0.00557434	0.016349	SF4	TM6SF2	KIAA0892
934	rs964184	11	116648917	XL.HDL.TG	Myocardial infarction	-0.15467	0.01402	-0.0487708	0.013873	ZNF259	BUD13	ZNF259
935	rs964184	11	116648917	XL.HDL.TG	Large vessel disease	-0.15467	0.01402	-0.0184	0.0505	ZNF259	BUD13	ZNF259
936	rs964184	11	116648917	XL.HDL.TG	Cardioembolic stroke	-0.15467	0.01402	-0.0127	0.0464	ZNF259	BUD13	ZNF259
937	rs964184	11	116648917	XL.HDL.TG	Small vessel disease	-0.15467	0.01402	-0.0253	0.0533	ZNF259	BUD13	ZNF259
938	rs964184	11	116648917	XL.HDL.TG	Ischemic stroke	-0.15467	0.01402	-0.0074	0.024	ZNF259	BUD13	ZNF259
939	rs964184	11	116648917	XL.HDL.TG	Coronary heart disease	-0.15467	0.01402	-0.049958	0.012399	ZNF259	BUD13	ZNF259
940	rs97384	11	61624181	XL.HDL.TG	Myocardial infarction	0.083919	0.01036	0.0191417	0.011688	FADS2	LOC100131326	FADS3
941	rs97384	11	61624181	XL.HDL.TG	Coronary heart disease	0.083919	0.01036	0.019915	0.010502	FADS2	LOC100131326	FADS3
942	rs10455872	6	161010118	XL.VLDL.TG	Cardioembolic stroke	-0.18067	0.02784	0.0167	0.0957	LPA	LPAL2	PLG
943	rs10455872	6	161010118	XL.VLDL.TG	Ischemic stroke	-0.18067	0.02784	0.0418	0.045	LPA	LPAL2	PLG
944	rs10455872	6	161010118	XL.VLDL.TG	Large vessel disease	-0.18067	0.02784	0.227	0.0906	LPA	LPAL2	PLG
945	rs10455872	6	161010118	XL.VLDL.TG	Myocardial infarction	-0.18067	0.02784	0.284774	0.026592	LPA	LPAL2	PLG
946	rs10455872	6	161010118	XL.VLDL.TG	Coronary heart disease	-0.18067	0.02784	0.318598	0.024399	LPA	LPAL2	PLG
947	rs10455872	6	161010118	XL.VLDL.TG	Small vessel disease	-0.18067	0.02784	-0.0807	0.1015	LPA	LPAL2	PLG
948	rs1168041	1	62960250	XL.VLDL.TG	Small vessel disease	0.083991	0.0112	-0.0781	0.0389	DOCK7	USP1	ANGPTL3
949	rs1168041	1	62960250	XL.VLDL.TG	Ischemic stroke	0.083991	0.0112	-0.0095	0.0178	DOCK7	USP1	ANGPTL3
950	rs1168041	1	62960250	XL.VLDL.TG	Myocardial infarction	0.083991	0.0112	0.00067632	0.011148	DOCK7	USP1	ANGPTL3
951	rs1168041	1	62960250	XL.VLDL.TG	Coronary heart disease	0.083991	0.0112	0.011014	0.01011	DOCK7	USP1	ANGPTL3
952	rs1168041	1	62960250	XL.VLDL.TG	Large vessel disease	0.083991	0.0112	-0.0152	0.0373	DOCK7	USP1	ANGPTL3
953	rs1168041	1	62960250	XL.VLDL.TG	Cardioembolic stroke	0.083991	0.0112	-0.0236	0.0353	DOCK7	USP1	ANGPTL3
954	rs1260326	2	27730940	XL.VLDL.TG	Large vessel disease	-0.093	0.01019	0.0392	0.0341	GCKR	FNDC4	LOC100130981
955	rs1260326	2	27730940	XL.VLDL.TG	Cardioembolic stroke	-0.093	0.01019	0.0401	0.0314	GCKR	FNDC4	LOC100130981
956	rs1260326	2	27730940	XL.VLDL.TG	Coronary heart disease	-0.093	0.01019	0.003257	0.00962	GCKR	FNDC4	LOC100130981
957	rs1260326	2	27730940	XL.VLDL.TG	Small vessel disease	-0.093	0.01019	-0.0441	0.0353	GCKR	FNDC4	LOC100130981
958	rs1260326	2	27730940	XL.VLDL.TG	Myocardial infarction	-0.093	0.01019	0.00111651	0.010666	GCKR	FNDC4	LOC100130981
959	rs1260326	2	27730940	XL.VLDL.TG	Ischemic stroke	-0.093	0.01019	0.012	0.0162	GCKR	FNDC4	LOC100130981
960	rs13234157	7	72971728	XL.VLDL.TG	Myocardial infarction	-0.1113	0.01516	0.00644804	0.016555	BCL7B	BAZ1B	TBL2
961	rs13234157	7	72971728	XL.VLDL.TG	Coronary heart disease	-0.1113	0.01516	0.005225	0.014834	BCL7B	BAZ1B	TBL2
962	rs13234157	7	72982874	XL.VLDL.TG	Ischemic stroke	-0.1113	0.01516	0.0184	0.0252	BCL7B	BAZ1B	TBL2
963	rs13234157	7	72982874	XL.VLDL.TG	Small vessel disease	-0.1113	0.01516	0.0089	0.0557	BCL7B	BAZ1B	TBL2
964	rs13234157	7	72982874	XL.VLDL.TG	Large vessel disease	-0.1113	0.01516	0.0049	0.0546	BCL7B	BAZ1B	TBL2
965	rs13234157	7	72982874	XL.VLDL.TG	Cardioembolic stroke	-0.1113	0.01516	0.0395	0.0502	BCL7B	BAZ1B	TBL2
966	rs186696265	6	161111700	XL.VLDL.TG	Myocardial infarction	-0.32556	0.05743	0.529852	0.052599			
967	rs186696265	6	161111700	XL.VLDL.TG	Coronary heart disease	-0.32556	0.05743	0.550351	0.048195			

968	rs4296389	2	21142994	XL.VLDL.TG	Myocardial infarction	-0.05923	0.01053	-0.0112562	0.01124		C2orf43	APOB
969	rs4296389	2	21142994	XL.VLDL.TG	Coronary heart disease	-0.05923	0.01053	-0.012159	0.010207		C2orf43	APOB
970	rs438811	19	45416741	XL.VLDL.TG	Myocardial infarction	0.080558	0.01285	0.0249193	0.014388	LOC100129500	APOE	APOC1
971	rs438811	19	45416741	XL.VLDL.TG	Coronary heart disease	0.080558	0.01285	0.013252	0.012834	LOC100129500	APOE	APOC1
972	rs72999033	19	19407718	XL.VLDL.TG	Cardioembolic stroke	-0.14446	0.0223	0.0972	0.0645			
973	rs72999033	19	19407718	XL.VLDL.TG	Small vessel disease	-0.14446	0.0223	0.0398	0.0744			
974	rs72999033	19	19366632	XL.VLDL.TG	Myocardial infarction	-0.14446	0.0223	-0.0370382	0.023873			
975	rs72999033	19	19366632	XL.VLDL.TG	Coronary heart disease	-0.14446	0.0223	-0.051959	0.021377			
976	rs72999033	19	19407718	XL.VLDL.TG	Large vessel disease	-0.14446	0.0223	0.1255	0.0702			
977	rs72999033	19	19407718	XL.VLDL.TG	Ischemic stroke	-0.14446	0.0223	0.0212	0.0335			
978	rs76975037	8	19851508	XL.VLDL.TG	Myocardial infarction	-0.12553	0.0174	-0.0665203	0.017954			
979	rs76975037	8	19851508	XL.VLDL.TG	Coronary heart disease	-0.12553	0.0174	-0.053571	0.016022			
980	rs76975037	8	19852134	XL.VLDL.TG	Ischemic stroke	-0.12553	0.0174	0.0376	0.0262			
981	rs76975037	8	19852134	XL.VLDL.TG	Small vessel disease	-0.12553	0.0174	0.0289	0.0596			
982	rs76975037	8	19852134	XL.VLDL.TG	Large vessel disease	-0.12553	0.0174	0.0167	0.0536			
983	rs76975037	8	19852134	XL.VLDL.TG	Cardioembolic stroke	-0.12553	0.0174	0.0885	0.0497			
984	rs964184	11	116648917	XL.VLDL.TG	Small vessel disease	-0.17881	0.01398	-0.0253	0.0533	ZNF259	BUD13	ZNF259
985	rs964184	11	116648917	XL.VLDL.TG	Large vessel disease	-0.17881	0.01398	-0.0184	0.0505	ZNF259	BUD13	ZNF259
986	rs964184	11	116648917	XL.VLDL.TG	Cardioembolic stroke	-0.17881	0.01398	-0.0127	0.0464	ZNF259	BUD13	ZNF259
987	rs964184	11	116648917	XL.VLDL.TG	Coronary heart disease	-0.17881	0.01398	-0.049958	0.012399	ZNF259	BUD13	ZNF259
988	rs964184	11	116648917	XL.VLDL.TG	Ischemic stroke	-0.17881	0.01398	-0.0074	0.024	ZNF259	BUD13	ZNF259
989	rs964184	11	116648917	XL.VLDL.TG	Myocardial infarction	-0.17881	0.01398	-0.0487708	0.013873	ZNF259	BUD13	ZNF259
990	rs113531395	17	4886829	XS.VLDL.TG	Coronary heart disease	-0.21548	0.03635	-0.046539	0.061719			
991	rs113531395	17	4886829	XS.VLDL.TG	Myocardial infarction	-0.21548	0.03635	-0.0573797	0.067105			
992	rs115849089	8	19912370	XS.VLDL.TG	Coronary heart disease	-0.15619	0.01718	-0.057989	0.015294			
993	rs115849089	8	19912370	XS.VLDL.TG	Myocardial infarction	-0.15619	0.01718	-0.0667741	0.017021			
994	rs1168041	1	62960250	XS.VLDL.TG	Coronary heart disease	0.093589	0.01174	0.011014	0.01011	DOCK7	USP1	ANGPTL3
995	rs1168041	1	62960250	XS.VLDL.TG	Myocardial infarction	0.093589	0.01174	0.00067632	0.011148	DOCK7	USP1	ANGPTL3
996	rs1168041	1	62960250	XS.VLDL.TG	Cardioembolic stroke	0.093589	0.01174	-0.0236	0.0353	DOCK7	USP1	ANGPTL3
997	rs1168041	1	62960250	XS.VLDL.TG	Ischemic stroke	0.093589	0.01174	-0.0095	0.0178	DOCK7	USP1	ANGPTL3
998	rs1168041	1	62960250	XS.VLDL.TG	Large vessel disease	0.093589	0.01174	-0.0152	0.0373	DOCK7	USP1	ANGPTL3
999	rs1168041	1	62960250	XS.VLDL.TG	Small vessel disease	0.093589	0.01174	-0.0781	0.0389	DOCK7	USP1	ANGPTL3
1000	rs1260326	2	27730940	XS.VLDL.TG	Ischemic stroke	-0.08106	0.01065	0.012	0.0162	GCKR	FNDC4	LOC100130981
1001	rs1260326	2	27730940	XS.VLDL.TG	Myocardial infarction	-0.08106	0.01065	0.00111651	0.010666	GCKR	FNDC4	LOC100130981
1002	rs1260326	2	27730940	XS.VLDL.TG	Cardioembolic stroke	-0.08106	0.01065	0.0401	0.0314	GCKR	FNDC4	LOC100130981
1003	rs1260326	2	27730940	XS.VLDL.TG	Small vessel disease	-0.08106	0.01065	-0.0441	0.0353	GCKR	FNDC4	LOC100130981
1004	rs1260326	2	27730940	XS.VLDL.TG	Large vessel disease	-0.08106	0.01065	0.0392	0.0341	GCKR	FNDC4	LOC100130981
1005	rs1260326	2	27730940	XS.VLDL.TG	Coronary heart disease	-0.08106	0.01065	0.003257	0.00962	GCKR	FNDC4	LOC100130981
1006	rs144064722	4	73406173	XS.VLDL.TG	Coronary heart disease	0.202156	0.0341	0.028313	0.031588			
1007	rs144064722	4	73406173	XS.VLDL.TG	Myocardial infarction	0.202156	0.0341	0.032534	0.034056			
1008	rs157594	19	45425175	XS.VLDL.TG	Coronary heart disease	0.11115	0.01174	0.021906	0.011888	LOC100129500	APOC1	APOC4

1009	rs157594	19	45425175	XS.VLDL.TG	Myocardial infarction	0.11115	0.01174	0.0265114	0.013237	LOC100129500	APOC1	APOC4
1010	rs174418	15	58687603	XS.VLDL.TG	Myocardial infarction	-0.08787	0.01048	-0.00891688	0.010502		LOC441726	LIPC
1011	rs174418	15	58687603	XS.VLDL.TG	Coronary heart disease	-0.08787	0.01048	-0.011405	0.009419		LOC441726	LIPC
1012	rs1848922	2	21471603	XS.VLDL.TG	Coronary heart disease	0.092776	0.0126	0.047369	0.011832		LOC100129278	LOC645949
1013	rs1848922	2	21471603	XS.VLDL.TG	Myocardial infarction	0.092776	0.0126	0.0301768	0.013003		LOC100129278	LOC645949
1014	rs1883711	20	39179822	XS.VLDL.TG	Myocardial infarction	0.148584	0.02474	0.0985739	0.040277		HSPEP1	MAFB
1015	rs1883711	20	39179822	XS.VLDL.TG	Coronary heart disease	0.148584	0.02474	0.133601	0.034913		HSPEP1	MAFB
1016	rs1883711	20	39179822	XS.VLDL.TG	Cardioembolic stroke	0.148584	0.02474	0.178	0.205		HSPEP1	MAFB
1017	rs2044332	15	58646641	XS.VLDL.TG	Myocardial infarction	0.092618	0.01526	-0.00567182	0.014995		LOC441726	LIPC
1018	rs2044332	15	58646641	XS.VLDL.TG	Coronary heart disease	0.092618	0.01526	0.005484	0.013358		LOC441726	LIPC
1019	rs247617	16	56990716	XS.VLDL.TG	Myocardial infarction	-0.11588	0.01146	-0.0261377	0.011415		HERPUD1	CETP
1020	rs247617	16	56993324	XS.VLDL.TG	Large vessel disease	-0.11588	0.01146	0.0256	0.0369		HERPUD1	CETP
1021	rs247617	16	56993324	XS.VLDL.TG	Ischemic stroke	-0.11588	0.01146	0.0102	0.0175		HERPUD1	CETP
1022	rs247617	16	56993324	XS.VLDL.TG	Cardioembolic stroke	-0.11588	0.01146	0.0074	0.0345		HERPUD1	CETP
1023	rs247617	16	56990716	XS.VLDL.TG	Coronary heart disease	-0.11588	0.01146	-0.030884	0.010212		HERPUD1	CETP
1024	rs247617	16	56993324	XS.VLDL.TG	Small vessel disease	-0.11588	0.01146	0.0027	0.0382		HERPUD1	CETP
1025	rs261334	15	58726744	XS.VLDL.TG	Cardioembolic stroke	-0.11838	0.01232	0.0623	0.0423	LIPC	LOC441726	ADAM10
1026	rs261334	15	58726744	XS.VLDL.TG	Ischemic stroke	-0.11838	0.01232	0.0383	0.0211	LIPC	LOC441726	ADAM10
1027	rs261334	15	58726744	XS.VLDL.TG	Large vessel disease	-0.11838	0.01232	0.0677	0.0452	LIPC	LOC441726	ADAM10
1028	rs261334	15	58726744	XS.VLDL.TG	Myocardial infarction	-0.11838	0.01232	-0.0402744	0.012395	LIPC	LOC441726	ADAM10
1029	rs261334	15	58726744	XS.VLDL.TG	Small vessel disease	-0.11838	0.01232	-0.0739	0.0451	LIPC	LOC441726	ADAM10
1030	rs261334	15	58726744	XS.VLDL.TG	Coronary heart disease	-0.11838	0.01232	-0.03756	0.011043	LIPC	LOC441726	ADAM10
1031	rs2878419	5	74655726	XS.VLDL.TG	Large vessel disease	0.060999	0.01045	-0.0577	0.0357	HMGCR	LOC728775	COL4A3BP
1032	rs2878419	5	74640490	XS.VLDL.TG	Coronary heart disease	0.060999	0.01045	0.030809	0.009553	HMGCR	LOC728775	COL4A3BP
1033	rs2878419	5	74655726	XS.VLDL.TG	Small vessel disease	0.060999	0.01045	0.0513	0.0371	HMGCR	LOC728775	COL4A3BP
1034	rs2878419	5	74655726	XS.VLDL.TG	Ischemic stroke	0.060999	0.01045	-0.0123	0.0168	HMGCR	LOC728775	COL4A3BP
1035	rs2878419	5	74655726	XS.VLDL.TG	Cardioembolic stroke	0.060999	0.01045	-0.0077	0.033	HMGCR	LOC728775	COL4A3BP
1036	rs2878419	5	74640490	XS.VLDL.TG	Myocardial infarction	0.060999	0.01045	0.0311687	0.010473	HMGCR	LOC728775	COL4A3BP
1037	rs34346326	7	73016181	XS.VLDL.TG	Coronary heart disease	-0.07646	0.01367	-0.010364	0.012808	MLXIPL MLXIPL MLXIPL	TBL2	VPS37D
1038	rs34346326	7	73016181	XS.VLDL.TG	Myocardial infarction	-0.07646	0.01367	-0.00956639	0.014338	MLXIPL MLXIPL MLXIPL	TBL2	VPS37D
1039	rs4609471	1	55493584	XS.VLDL.TG	Coronary heart disease	-0.17343	0.02981	-0.088176	0.037828		BSND	PCSK9
1040	rs4609471	1	55493584	XS.VLDL.TG	Myocardial infarction	-0.17343	0.02981	-0.110147	0.042779		BSND	PCSK9
1041	rs58542926	19	19610596	XS.VLDL.TG	Small vessel disease	-0.15327	0.02107	0.0136	0.0675	TM6SF2	HAPLN4	SF4
1042	rs58542926	19	19379549	XS.VLDL.TG	Myocardial infarction	-0.15327	0.02107	-0.0459691	0.020412	TM6SF2	HAPLN4	SF4
1043	rs58542926	19	19610596	XS.VLDL.TG	Cardioembolic stroke	-0.15327	0.02107	0.1322	0.0595	TM6SF2	HAPLN4	SF4
1044	rs58542926	19	19610596	XS.VLDL.TG	Large vessel disease	-0.15327	0.02107	0.0518	0.0641	TM6SF2	HAPLN4	SF4
1045	rs58542926	19	19379549	XS.VLDL.TG	Coronary heart disease	-0.15327	0.02107	-0.051253	0.018343	TM6SF2	HAPLN4	SF4

1046	rs58542926	19	19610596	XS.VLDL.TG	Ischemic stroke	-0.15327	0.02107	0.0224	0.0304	TM6SF2	HAPLN4	SF4
1047	rs6511720	19	11202306	XS.VLDL.TG	Small vessel disease	-0.14492	0.01736	-0.013	0.0549	LDLR	SMARCA4	SPC24
1048	rs6511720	19	11202306	XS.VLDL.TG	Large vessel disease	-0.14492	0.01736	-0.0924	0.0525	LDLR	SMARCA4	SPC24
1049	rs6511720	19	11202306	XS.VLDL.TG	Cardioembolic stroke	-0.14492	0.01736	-0.0567	0.048	LDLR	SMARCA4	SPC24
1050	rs6511720	19	11202306	XS.VLDL.TG	Ischemic stroke	-0.14492	0.01736	-0.0708	0.0249	LDLR	SMARCA4	SPC24
1051	rs6511720	19	11202306	XS.VLDL.TG	Myocardial infarction	-0.14492	0.01736	-0.100786	0.01894	LDLR	SMARCA4	SPC24
1052	rs6511720	19	11202306	XS.VLDL.TG	Coronary heart disease	-0.14492	0.01736	-0.125298	0.016945	LDLR	SMARCA4	SPC24
1053	rs6544366	2	21204025	XS.VLDL.TG	Myocardial infarction	-0.12061	0.01144	-0.0116187	0.01142		C2orf43	APOB
1054	rs6544366	2	21204025	XS.VLDL.TG	Large vessel disease	-0.12061	0.01144	0.0118	0.0407		C2orf43	APOB
1055	rs6544366	2	21204025	XS.VLDL.TG	Coronary heart disease	-0.12061	0.01144	-0.003091	0.0103		C2orf43	APOB
1056	rs6544366	2	21204025	XS.VLDL.TG	Cardioembolic stroke	-0.12061	0.01144	-0.0062	0.0377		C2orf43	APOB
1057	rs6544366	2	21204025	XS.VLDL.TG	Small vessel disease	-0.12061	0.01144	0.0259	0.0416		C2orf43	APOB
1058	rs6544366	2	21204025	XS.VLDL.TG	Ischemic stroke	-0.12061	0.01144	0.0109	0.0188		C2orf43	APOB
1059	rs72660594	1	55636240	XS.VLDL.TG	Coronary heart disease	-0.20082	0.02886	-0.080262	0.046373			
1060	rs72660594	1	55636240	XS.VLDL.TG	Myocardial infarction	-0.20082	0.02886	-0.0986201	0.051013			
1061	rs964184	11	116648917	XS.VLDL.TG	Small vessel disease	-0.21633	0.01453	-0.0253	0.0533	ZNF259	BUD13	ZNF259
1062	rs964184	11	116648917	XS.VLDL.TG	Myocardial infarction	-0.21633	0.01453	-0.0487708	0.013873	ZNF259	BUD13	ZNF259
1063	rs964184	11	116648917	XS.VLDL.TG	Cardioembolic stroke	-0.21633	0.01453	-0.0127	0.0464	ZNF259	BUD13	ZNF259
1064	rs964184	11	116648917	XS.VLDL.TG	Coronary heart disease	-0.21633	0.01453	-0.049958	0.012399	ZNF259	BUD13	ZNF259
1065	rs964184	11	116648917	XS.VLDL.TG	Large vessel disease	-0.21633	0.01453	-0.0184	0.0505	ZNF259	BUD13	ZNF259
1066	rs964184	11	116648917	XS.VLDL.TG	Ischemic stroke	-0.21633	0.01453	-0.0074	0.024	ZNF259	BUD13	ZNF259
1067	rs10455872	6	161010118	XXL.VLDL.TG	Large vessel disease	-0.19364	0.02787	0.227	0.0906	LPA	LPAL2	PLG
1068	rs10455872	6	161010118	XXL.VLDL.TG	Myocardial infarction	-0.19364	0.02787	0.284774	0.026592	LPA	LPAL2	PLG
1069	rs10455872	6	161010118	XXL.VLDL.TG	Coronary heart disease	-0.19364	0.02787	0.318598	0.024399	LPA	LPAL2	PLG
1070	rs10455872	6	161010118	XXL.VLDL.TG	Ischemic stroke	-0.19364	0.02787	0.0418	0.045	LPA	LPAL2	PLG
1071	rs10455872	6	161010118	XXL.VLDL.TG	Cardioembolic stroke	-0.19364	0.02787	0.0167	0.0957	LPA	LPAL2	PLG
1072	rs10455872	6	161010118	XXL.VLDL.TG	Small vessel disease	-0.19364	0.02787	-0.0807	0.1015	LPA	LPAL2	PLG
1073	rs1168041	1	62960250	XXL.VLDL.TG	Myocardial infarction	0.077153	0.01121	0.00067632	0.011148	DOCK7	USP1	ANGPTL3
1074	rs1168041	1	62960250	XXL.VLDL.TG	Coronary heart disease	0.077153	0.01121	0.011014	0.01011	DOCK7	USP1	ANGPTL3
1075	rs1168041	1	62960250	XXL.VLDL.TG	Ischemic stroke	0.077153	0.01121	-0.0095	0.0178	DOCK7	USP1	ANGPTL3
1076	rs1168041	1	62960250	XXL.VLDL.TG	Small vessel disease	0.077153	0.01121	-0.0781	0.0389	DOCK7	USP1	ANGPTL3
1077	rs1168041	1	62960250	XXL.VLDL.TG	Large vessel disease	0.077153	0.01121	-0.0152	0.0373	DOCK7	USP1	ANGPTL3
1078	rs1168041	1	62960250	XXL.VLDL.TG	Cardioembolic stroke	0.077153	0.01121	-0.0236	0.0353	DOCK7	USP1	ANGPTL3
1079	rs1260326	2	27730940	XXL.VLDL.TG	Cardioembolic stroke	-0.09349	0.01021	0.0401	0.0314	GCKR	FNDC4	LOC100130981
1080	rs1260326	2	27730940	XXL.VLDL.TG	Ischemic stroke	-0.09349	0.01021	0.012	0.0162	GCKR	FNDC4	LOC100130981
1081	rs1260326	2	27730940	XXL.VLDL.TG	Large vessel disease	-0.09349	0.01021	0.0392	0.0341	GCKR	FNDC4	LOC100130981
1082	rs1260326	2	27730940	XXL.VLDL.TG	Coronary heart disease	-0.09349	0.01021	0.003257	0.00962	GCKR	FNDC4	LOC100130981
1083	rs1260326	2	27730940	XXL.VLDL.TG	Myocardial infarction	-0.09349	0.01021	0.00111651	0.010666	GCKR	FNDC4	LOC100130981
1084	rs1260326	2	27730940	XXL.VLDL.TG	Small vessel disease	-0.09349	0.01021	-0.0441	0.0353	GCKR	FNDC4	LOC100130981
1085	rs13233571	7	72971231	XXL.VLDL.TG	Myocardial infarction	-0.09437	0.01519	0.00652852	0.016594	BCL7B	BAZ1B	TBL2
1086	rs13233571	7	72971231	XXL.VLDL.TG	Large vessel disease	-0.09437	0.01519	0.0028	0.0547	BCL7B	BAZ1B	TBL2

1087	rs13233571	7	72971231	XXL.VLDL.TG	Small vessel disease	-0.09437	0.01519	0.0093	0.0568	BCL7B	BAZ1B	TBL2
1088	rs13233571	7	72971231	XXL.VLDL.TG	Ischemic stroke	-0.09437	0.01519	0.0371	0.0253	BCL7B	BAZ1B	TBL2
1089	rs13233571	7	72971231	XXL.VLDL.TG	Cardioembolic stroke	-0.09437	0.01519	0.0631	0.05	BCL7B	BAZ1B	TBL2
1090	rs13233571	7	72971231	XXL.VLDL.TG	Coronary heart disease	-0.09437	0.01519	0.00613	0.014865	BCL7B	BAZ1B	TBL2
1091	rs483082	19	45416178	XXL.VLDL.TG	Myocardial infarction	0.0972	0.01209	0.0237461	0.014422	APOC1	APOE	APOC1
1092	rs483082	19	45416178	XXL.VLDL.TG	Coronary heart disease	0.0972	0.01209	0.011561	0.012875	APOC1	APOE	APOC1
1093	rs72999033	19	19407718	XXL.VLDL.TG	Ischemic stroke	-0.12619	0.02101	0.0212	0.0335			
1094	rs72999033	19	19407718	XXL.VLDL.TG	Large vessel disease	-0.12619	0.02101	0.1255	0.0702			
1095	rs72999033	19	19366632	XXL.VLDL.TG	Coronary heart disease	-0.12619	0.02101	-0.051959	0.021377			
1096	rs72999033	19	19407718	XXL.VLDL.TG	Small vessel disease	-0.12619	0.02101	0.0398	0.0744			
1097	rs72999033	19	19366632	XXL.VLDL.TG	Myocardial infarction	-0.12619	0.02101	-0.0370382	0.023873			
1098	rs72999033	19	19407718	XXL.VLDL.TG	Cardioembolic stroke	-0.12619	0.02101	0.0972	0.0645			
1099	rs77729186	8	19844222	XXL.VLDL.TG	Cardioembolic stroke	-0.11594	0.01693	0.0785	0.0518			
1100	rs77729186	8	19844222	XXL.VLDL.TG	Ischemic stroke	-0.11594	0.01693	0.0319	0.027			
1101	rs77729186	8	19826318	XXL.VLDL.TG	Coronary heart disease	-0.11594	0.01693	-0.051641	0.015873			
1102	rs77729186	8	19844222	XXL.VLDL.TG	Small vessel disease	-0.11594	0.01693	0.0113	0.0612			
1103	rs77729186	8	19844222	XXL.VLDL.TG	Large vessel disease	-0.11594	0.01693	-0.0069	0.0559			
1104	rs77729186	8	19826318	XXL.VLDL.TG	Myocardial infarction	-0.11594	0.01693	-0.0663204	0.017713			
1105	rs821840	16	56993886	XXL.VLDL.TG	Coronary heart disease	-0.06546	0.01164	-0.040648	0.011129	CETP	HERPUD1	CETP
1106	rs821840	16	56993886	XXL.VLDL.TG	Myocardial infarction	-0.06546	0.01164	-0.0346335	0.012304	CETP	HERPUD1	CETP
1107	rs964184	11	116648917	XXL.VLDL.TG	Large vessel disease	-0.1529	0.01401	-0.0184	0.0505	ZNF259	BUD13	ZNF259
1108	rs964184	11	116648917	XXL.VLDL.TG	Ischemic stroke	-0.1529	0.01401	-0.0074	0.024	ZNF259	BUD13	ZNF259
1109	rs964184	11	116648917	XXL.VLDL.TG	Cardioembolic stroke	-0.1529	0.01401	-0.0127	0.0464	ZNF259	BUD13	ZNF259
1110	rs964184	11	116648917	XXL.VLDL.TG	Myocardial infarction	-0.1529	0.01401	-0.0487708	0.013873	ZNF259	BUD13	ZNF259
1111	rs964184	11	116648917	XXL.VLDL.TG	Small vessel disease	-0.1529	0.01401	-0.0253	0.0533	ZNF259	BUD13	ZNF259
1112	rs964184	11	116648917	XXL.VLDL.TG	Coronary heart disease	-0.1529	0.01401	-0.049958	0.012399	ZNF259	BUD13	ZNF259

Abbreviation: Apo, apolipoprotein; HDL-C, high-density lipoprotein cholesterol; IDL, intermediate density lipoprotein; LDL-C, low-density lipoprotein cholesterol; SD, standard deviation; TC, total cholesterol; TG, triglycerides; VLDL, very low density lipoprotein; XXL.VLDL.TG, triglycerides in largest VLDL; XL.VLDL.TG, triglycerides in very large VLDL; L.VLDL.TG, triglycerides in large VLDL; M.VLDL.TG, triglycerides in medium VLDL; S.VLDL.TG, triglycerides in small VLDL; XS.VLDL.TG, triglycerides in very small VLDL; IDL.TG, triglycerides in IDL; XL.HDL.TG, triglycerides in very large HDL; S.HDL.TG, triglycerides in small HDL

eTable 2. Summary information of instrumental variables in TSMR analysis.

Exposure	Outcome	R^2(%)	Number of SNPs	F. value	Number of Outliers
ApoA1	Cardioembolic stroke	5.03	10	109.4	0
ApoA1	Coronary heart disease	5.67	13	95.51	2
ApoA1	Ischemic stroke	5.03	10	109.4	0
ApoA1	Large vessel disease	5.03	10	109.4	0
ApoA1	Myocardial infarction	5.67	13	95.51	2
ApoA1	Small vessel disease	5.03	10	109.4	0
ApoB	Cardioembolic stroke	3.93	12	70.4	0
ApoB	Coronary heart disease	9.93	25	91.1	3
ApoB	Ischemic stroke	3.7	11	72.29	0
ApoB	Large vessel disease	3.7	11	72.29	0
ApoB	Myocardial infarction	9.93	25	91.1	3
ApoB	Small vessel disease	3.7	11	72.29	0
HDL.C	Cardioembolic stroke	5.95	14	97.36	0
HDL.C	Coronary heart disease	6.8	17	92.46	3
HDL.C	Ischemic stroke	5.95	14	97.36	0
HDL.C	Large vessel disease	5.95	14	97.36	0
HDL.C	Myocardial infarction	6.8	17	92.46	1
HDL.C	Small vessel disease	5.95	14	97.36	0
IDL.TG	Cardioembolic stroke	5.46	11	101.22	0
IDL.TG	Coronary heart disease	10.85	27	86.75	3
IDL.TG	Ischemic stroke	5.21	10	105.83	0
IDL.TG	Large vessel disease	5.21	10	105.83	0
IDL.TG	Myocardial infarction	10.85	27	86.75	1
IDL.TG	Small vessel disease	5.21	10	105.83	0
L.VLDL.TG	Cardioembolic stroke	2.42	6	87.9	0
L.VLDL.TG	Coronary heart disease	3.29	9	80.18	2
L.VLDL.TG	Ischemic stroke	2.42	6	87.9	0
L.VLDL.TG	Large vessel disease	2.42	6	87.9	0
L.VLDL.TG	Myocardial infarction	3.29	9	80.18	2
L.VLDL.TG	Small vessel disease	2.42	6	87.9	0
LDL.C	Cardioembolic stroke	4.57	15	68.74	0
LDL.C	Coronary heart disease	14.69	31	119.55	3
LDL.C	Ischemic stroke	4.35	14	69.96	0
LDL.C	Large vessel disease	4.35	14	69.96	0
LDL.C	Myocardial infarction	14.69	31	119.55	3
LDL.C	Small vessel disease	4.35	14	69.96	0
M.VLDL.TG	Cardioembolic stroke	3.26	8	89.38	0
M.VLDL.TG	Coronary heart disease	4.52	13	77.27	1
M.VLDL.TG	Ischemic stroke	3.26	8	89.38	0
M.VLDL.TG	Large vessel disease	3.26	8	89.38	0
M.VLDL.TG	Myocardial infarction	4.52	13	77.27	1
M.VLDL.TG	Small vessel disease	3.26	8	89.38	0
S.HDL.TG	Cardioembolic stroke	3.2	7	101.63	0
S.HDL.TG	Coronary heart disease	4.57	13	79.43	3
S.HDL.TG	Ischemic stroke	3.2	7	101.63	0
S.HDL.TG	Large vessel disease	3.2	7	101.63	0
S.HDL.TG	Myocardial infarction	4.57	13	79.43	3
S.HDL.TG	Small vessel disease	3.2	7	101.63	0
S.VLDL.TG	Cardioembolic stroke	3.64	9	90.54	0
S.VLDL.TG	Coronary heart disease	6.26	17	84.68	3
S.VLDL.TG	Ischemic stroke	3.64	9	90.54	0
S.VLDL.TG	Large vessel disease	3.64	9	90.54	0
S.VLDL.TG	Myocardial infarction	6.09	16	87.31	2
S.VLDL.TG	Small vessel disease	3.64	9	90.54	0
TC	Cardioembolic stroke	3.18	13	54.29	0
TC	Coronary heart disease	9.75	25	92.73	1

TC	Ischemic stroke	2.97	12	54.87	1
TC	Large vessel disease	2.97	12	54.87	0
TC	Myocardial infarction	9.75	25	92.73	1
TC	Small vessel disease	2.97	12	54.87	0
TG	Cardioembolic stroke	2.96	6	109.5	0
TG	Coronary heart disease	5	12	94.36	3
TG	Ischemic stroke	2.96	6	109.5	0
TG	Large vessel disease	2.96	6	109.5	0
TG	Myocardial infarction	5	12	94.36	1
TG	Small vessel disease	2.96	6	109.5	0
XL.HDL.TG	Cardioembolic stroke	10.4	13	192.07	0
XL.HDL.TG	Coronary heart disease	15.15	27	142.26	2
XL.HDL.TG	Ischemic stroke	10.4	13	192.07	0
XL.HDL.TG	Large vessel disease	10.4	13	192.07	0
XL.HDL.TG	Myocardial infarction	15.15	27	142.26	0
XL.HDL.TG	Small vessel disease	10.4	13	192.07	0
XL.VLDL.TG	Cardioembolic stroke	2.43	7	76.66	0
XL.VLDL.TG	Coronary heart disease	3.01	10	66.86	5
XL.VLDL.TG	Ischemic stroke	2.43	7	76.66	0
XL.VLDL.TG	Large vessel disease	2.43	7	76.66	0
XL.VLDL.TG	Myocardial infarction	3.01	10	66.86	4
XL.VLDL.TG	Small vessel disease	2.43	7	76.66	0
XS.VLDL.TG	Cardioembolic stroke	4.5	10	90.66	0
XS.VLDL.TG	Coronary heart disease	7.53	20	78.42	1
XS.VLDL.TG	Ischemic stroke	4.26	9	95.22	0
XS.VLDL.TG	Large vessel disease	4.26	9	95.22	0
XS.VLDL.TG	Myocardial infarction	7.53	20	78.42	1
XS.VLDL.TG	Small vessel disease	4.26	9	95.22	0
XXL.VLDL.TG	Cardioembolic stroke	2.09	7	65.56	0
XXL.VLDL.TG	Coronary heart disease	2.59	9	63.62	4
XXL.VLDL.TG	Ischemic stroke	2.09	7	65.56	0
XXL.VLDL.TG	Large vessel disease	2.09	7	65.56	0
XXL.VLDL.TG	Myocardial infarction	2.59	9	63.62	3
XXL.VLDL.TG	Small vessel disease	2.09	7	65.56	0

Number of Outliers was identified form the MR-PRESSO method.

Abbreviation: Apo, apolipoprotein; HDL-C, high-density lipoprotein cholesterol; IDL, intermediate density lipoprotein; LDL-C, low-density lipoprotein cholesterol; SD, standard deviation; TC, total cholesterol; TG, triglycerides; VLDL, very low density lipoprotein; XXL.VLDL.TG, triglycerides in largest VLDL; XL.VLDL.TG, triglycerides in very large VLDL; L.VLDL.TG, triglycerides in large VLDL; M.VLDL.TG, triglycerides in medium VLDL; S.VLDL.TG, triglycerides in small VLDL; XS.VLDL.TG, triglycerides in very small VLDL; IDL.TG, triglycerides in IDL; XL.HDL.TG, triglycerides in very large HDL; S.HDL.TG, triglycerides in small HDL

eTable 3. In-depth analysis by the MR-TRYX method.

Exposure	Outcome	Raw.OR	Raw.P	Removed.all.outliers.OR	Removed.all.P	Removed.candidate.outliers.OR	Removed.candidate.outliers.P	Adjusted.OR	Adjusted.P	Mark1	Mark2	Mark3
IDL.TG	IS	0.95(0.84 to 1.08)	0.462	0.89(0.84 to 0.94)	0.004	0.89(0.84 to 0.94)	0.004	0.91(0.85 to 0.98)	0.031			
S.VLDL.TG	IS	1.01(0.88 to 1.18)	0.855	0.97(0.90 to 1.06)	0.535	0.97(0.90 to 1.06)	0.535	0.99(0.90 to 1.10)	0.893	√	√	√
TC	IS	1.01(0.83 to 1.23)	0.928	0.96(0.81 to 1.14)	0.679	0.96(0.81 to 1.14)	0.679	0.96(0.82 to 1.13)	0.664	√	√	√
S.HDL.TG	IS	0.99(0.83 to 1.18)	0.943	0.95(0.85 to 1.05)	0.346	0.95(0.85 to 1.05)	0.346	0.96(0.86 to 1.07)	0.470	√	√	√
XS.VLDL.TG	IS	0.97(0.84 to 1.11)	0.643	0.91(0.84 to 0.98)	0.048	0.91(0.84 to 0.98)	0.048	0.93(0.85 to 1.01)	0.136			√
TC	CHD	1.44(1.31 to 1.59)	0.000	1.40(1.28 to 1.52)	0.000	1.40(1.28 to 1.52)	0.000	1.40(1.29 to 1.52)	0.000	√	√	√
XL.VLDL.TG	CHD	0.92(0.59 to 1.45)	0.735	1.06(0.96 to 1.17)	0.325	1.06(0.96 to 1.17)	0.325	1.04(0.98 to 1.11)	0.254	√	√	√
XS.VLDL.TG	CHD	1.30(1.19 to 1.42)	0.000	1.26(1.17 to 1.36)	0.000	1.26(1.17 to 1.36)	0.000	1.26(1.18 to 1.35)	0.000	√	√	√
ApoB	CHD	1.49(1.34 to 1.66)	0.000	1.44(1.32 to 1.57)	0.000	1.44(1.32 to 1.57)	0.000	1.44(1.32 to 1.57)	0.000	√	√	√
TG	CHD	1.28(1.13 to 1.46)	0.003	1.28(1.15 to 1.43)	0.002	1.28(1.15 to 1.43)	0.002	1.29(1.17 to 1.42)	0.000	√	√	√
M.VLDL.TG	CHD	1.19(1.06 to 1.34)	0.011	1.22(1.10 to 1.36)	0.004	1.22(1.10 to 1.36)	0.004	1.22(1.10 to 1.35)	0.003	√	√	√
L.VLDL.TG	CHD	1.08(0.75 to 1.56)	0.685	1.23(1.11 to 1.37)	0.005	1.23(1.11 to 1.37)	0.005	1.23(1.12 to 1.36)	0.003			
S.VLDL.TG	CHD	1.23(1.10 to 1.38)	0.002	1.20(1.10 to 1.32)	0.002	1.20(1.10 to 1.32)	0.002	1.20(1.11 to 1.30)	0.000	√	√	√
XL.HDL.TG	CHD	1.09(1.03 to 1.15)	0.005	1.10(1.05 to 1.15)	0.001	1.10(1.05 to 1.15)	0.001	1.10(1.05 to 1.15)	0.001	√	√	√
IDL.TG	CHD	1.33(1.21 to 1.47)	0.000	1.28(1.20 to 1.38)	0.000	1.28(1.20 to 1.38)	0.000	1.28(1.20 to 1.37)	0.000	√	√	√
XXL.VLDL.TG	CHD	1.03(0.67 to 1.56)	0.910	1.09(0.94 to 1.26)	0.327	1.09(0.94 to 1.26)	0.327	1.07(0.97 to 1.18)	0.200	√	√	√
ApoA1	CHD	1.04(0.93 to 1.16)	0.484	1.13(1.03 to 1.25)	0.031	1.13(1.03 to 1.25)	0.031	1.12(1.03 to 1.23)	0.024			
LDL.C	CHD	1.39(1.27 to 1.51)	0.000	1.35(1.26 to 1.44)	0.000	1.35(1.26 to 1.44)	0.000	1.35(1.27 to 1.44)	0.000	√	√	√
S.HDL.TG	CHD	1.29(1.10 to 1.53)	0.010	1.25(1.14 to 1.38)	0.001	1.25(1.14 to 1.38)	0.001	1.24(1.14 to 1.35)	0.000	√	√	√
HDL.C	CHD	1.00(0.90 to 1.12)	0.929	1.09(0.99 to 1.20)	0.099	1.09(0.99 to 1.20)	0.099	1.07(0.99 to 1.16)	0.127	√	√	√
ApoB	MI	1.47(1.30 to 1.65)	0.000	1.41(1.29 to 1.54)	0.000	1.41(1.29 to 1.54)	0.000	1.41(1.29 to 1.54)	0.000	√	√	√
LDL.C	MI	1.37(1.24 to 1.50)	0.000	1.33(1.24 to 1.42)	0.000	1.33(1.24 to 1.42)	0.000	1.33(1.25 to 1.42)	0.000	√	√	√
XS.VLDL.TG	MI	1.28(1.18 to 1.38)	0.000	1.25(1.16 to 1.34)	0.000	1.25(1.16 to 1.34)	0.000	1.25(1.17 to 1.34)	0.000	√	√	√
IDL.TG	MI	1.30(1.19 to 1.42)	0.000	1.27(1.19 to 1.37)	0.000	1.27(1.19 to 1.37)	0.000	1.27(1.19 to 1.37)	0.000	√	√	√
L.VLDL.TG	MI	1.09(0.78 to 1.54)	0.622	1.20(1.08 to 1.33)	0.013	1.20(1.08 to 1.33)	0.013	1.18(1.09 to 1.29)	0.005			
HDL.C	MI	1.01(0.92 to 1.11)	0.828	0.98(0.90 to 1.07)	0.620	0.98(0.90 to 1.07)	0.620	0.98(0.90 to 1.06)	0.613	√	√	√
ApoA1	MI	1.04(0.94 to 1.15)	0.462	1.12(1.02 to 1.23)	0.040	1.12(1.02 to 1.23)	0.040	1.11(1.03 to 1.21)	0.025			
S.HDL.TG	MI	1.27(1.09 to 1.48)	0.009	1.24(1.15 to 1.34)	0.000	1.24(1.15 to 1.34)	0.000	1.23(1.14 to 1.32)	0.000	√	√	√

XL.VLDL.TG	MI	0.93(0.61 to 1.42)	0.741	1.08(0.96 to 1.22)	0.251	1.08(0.96 to 1.22)	0.251	1.02(0.92 to 1.12)	0.767	√	√	√
S.VLDL.TG	MI	1.23(1.11 to 1.36)	0.001	1.25(1.14 to 1.37)	0.000	1.25(1.14 to 1.37)	0.000	1.24(1.14 to 1.36)	0.000	√	√	√
TC	MI	1.40(1.27 to 1.53)	0.000	1.36(1.25 to 1.48)	0.000	1.36(1.25 to 1.48)	0.000	1.36(1.25 to 1.48)	0.000	√	√	√
TG	MI	1.28(1.12 to 1.45)	0.003	1.24(1.11 to 1.38)	0.003	1.24(1.11 to 1.38)	0.003	1.24(1.12 to 1.37)	0.002	√	√	√
XXL.VLDL.TG	MI	1.04(0.70 to 1.54)	0.865	1.13(0.96 to 1.33)	0.211	1.13(0.96 to 1.33)	0.211	1.13(1.00 to 1.28)	0.093	√	√	√
M.VLDL.TG	MI	1.19(1.08 to 1.32)	0.006	1.22(1.12 to 1.34)	0.001	1.22(1.12 to 1.34)	0.001	1.22(1.12 to 1.33)	0.001	√	√	√

Mark1: √ represent consistent results of Removed.all.outliers.OR and Raw.OR

Mark2: √ represent consistent results of Removed.candidate.outliers.OR and Raw.OR

Mark3: √ represent consistent results of Adjusted.OR and Raw.OR

The consistent results were defined as the same causal directions and significance.

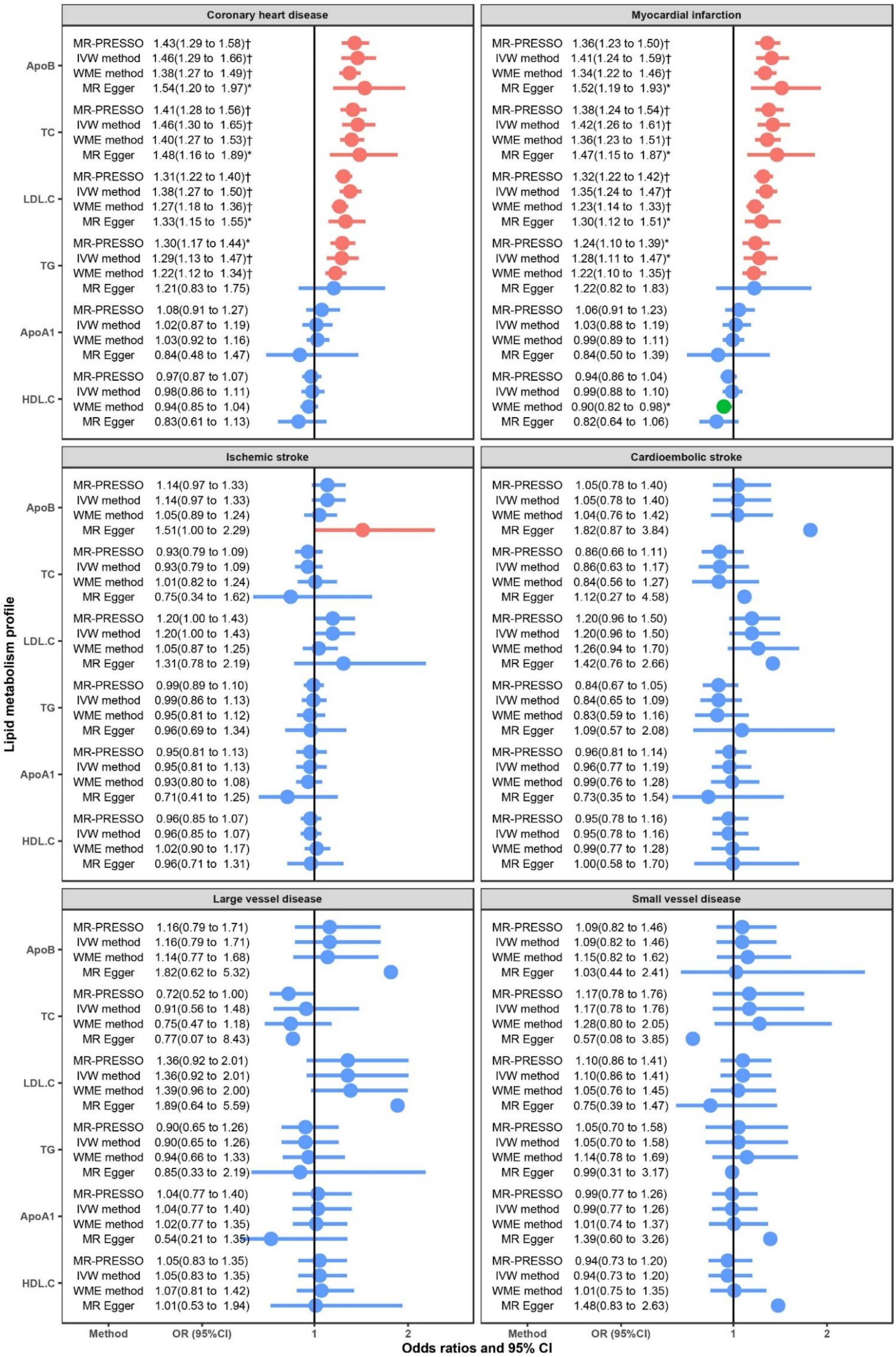

Coronary heart disease
ApoB
MR-PRESSO 1.43(1.29 to 1.58)†
IVW method 1.46(1.29 to 1.66)†
WME method 1.38(1.27 to 1.49)†
MR Egger 1.54(1.20 to 1.97)*
TC
MR-PRESSO 1.41(1.28 to 1.56)†
IVW method 1.46(1.30 to 1.65)†
WME method 1.40(1.27 to 1.53)†
MR Egger 1.48(1.16 to 1.89)*
LDL.C
MR-PRESSO 1.31(1.22 to 1.40)†
IVW method 1.38(1.27 to 1.50)†
WME method 1.27(1.18 to 1.36)†
MR Egger 1.33(1.15 to 1.55)*
TG
MR-PRESSO 1.30(1.17 to 1.44)*
IVW method 1.29(1.13 to 1.47)†
WME method 1.22(1.12 to 1.34)†
MR Egger 1.21(0.83 to 1.75)
ApoA1
MR-PRESSO 1.08(0.91 to 1.27)
IVW method 1.02(0.87 to 1.19)
WME method 1.03(0.92 to 1.16)
MR Egger 0.84(0.48 to 1.47)
HDL.C
MR-PRESSO 0.97(0.87 to 1.07)
IVW method 0.98(0.86 to 1.11)
WME method 0.94(0.85 to 1.04)
MR Egger 0.83(0.61 to 1.13)
Myocardial infarction
MR-PRESSO 1.36(1.23 to 1.50)†
IVW method 1.41(1.24 to 1.59)†
WME method 1.34(1.22 to 1.46)†
MR Egger 1.52(1.19 to 1.93)*
MR-PRESSO 1.38(1.24 to 1.54)†
IVW method 1.42(1.26 to 1.61)†
WME method 1.36(1.23 to 1.51)†
MR Egger 1.47(1.15 to 1.87)*
MR-PRESSO 1.32(1.22 to 1.42)†
IVW method 1.35(1.24 to 1.47)†
WME method 1.23(1.14 to 1.33)†
MR Egger 1.30(1.12 to 1.51)*
MR-PRESSO 1.24(1.10 to 1.39)*
IVW method 1.28(1.11 to 1.47)*
WME method 1.22(1.10 to 1.35)†
MR Egger 1.22(0.82 to 1.83)
MR-PRESSO 1.06(0.91 to 1.23)
IVW method 1.03(0.88 to 1.19)
WME method 0.99(0.89 to 1.11)
MR Egger 0.84(0.50 to 1.39)
MR-PRESSO 0.94(0.86 to 1.04)
IVW method 0.99(0.88 to 1.10)
WME method 0.90(0.82 to 0.98)*
MR Egger 0.82(0.64 to 1.06)
Ischemic stroke
ApoB
MR-PRESSO 1.14(0.97 to 1.33)
IVW method 1.14(0.97 to 1.33)
WME method 1.05(0.89 to 1.24)
MR Egger 1.51(1.00 to 2.29)
TC
MR-PRESSO 0.93(0.79 to 1.09)
IVW method 0.93(0.79 to 1.09)
WME method 1.01(0.82 to 1.24)
MR Egger 0.75(0.34 to 1.62)
LDL.C
MR-PRESSO 1.20(1.00 to 1.43)
IVW method 1.20(1.00 to 1.43)
WME method 1.05(0.87 to 1.25)
MR Egger 1.31(0.78 to 2.19)
TG
MR-PRESSO 0.99(0.89 to 1.10)
IVW method 0.99(0.86 to 1.13)
WME method 0.95(0.81 to 1.12)
MR Egger 0.96(0.69 to 1.34)
ApoA1
MR-PRESSO 0.95(0.81 to 1.13)
IVW method 0.95(0.81 to 1.13)
WME method 0.93(0.80 to 1.08)
MR Egger 0.71(0.41 to 1.25)
HDL.C
MR-PRESSO 0.96(0.85 to 1.07)
IVW method 0.96(0.85 to 1.07)
WME method 1.02(0.90 to 1.17)
MR Egger 0.96(0.71 to 1.31)
Lipid metabolism profile
Cardioembolic stroke
MR-PRESSO 1.05(0.78 to 1.40)
IVW method 1.05(0.78 to 1.40)
WME method 1.04(0.76 to 1.42)
MR Egger 1.82(0.87 to 3.84)
MR-PRESSO 0.86(0.66 to 1.11)
IVW method 0.86(0.63 to 1.17)
WME method 0.84(0.56 to 1.27)
MR Egger 1.12(0.27 to 4.58)
MR-PRESSO 1.20(0.96 to 1.50)
IVW method 1.20(0.96 to 1.50)
WME method 1.26(0.94 to 1.70)
MR Egger 1.42(0.76 to 2.66)
MR-PRESSO 0.84(0.67 to 1.05)
IVW method 0.84(0.65 to 1.09)
WME method 0.83(0.59 to 1.16)
MR Egger 1.09(0.57 to 2.08)
MR-PRESSO 0.96(0.81 to 1.14)
IVW method 0.96(0.77 to 1.19)
WME method 0.99(0.76 to 1.28)
MR Egger 0.73(0.35 to 1.54)
MR-PRESSO 0.95(0.78 to 1.16)
IVW method 0.95(0.78 to 1.16)
WME method 0.99(0.77 to 1.28)
MR Egger 1.00(0.58 to 1.70)
Large vessel disease
ApoB
MR-PRESSO 1.16(0.79 to 1.71)
IVW method 1.16(0.79 to 1.71)
WME method 1.14(0.77 to 1.68)
MR Egger 1.82(0.62 to 5.32)
TC
MR-PRESSO 0.72(0.52 to 1.00)
IVW method 0.91(0.56 to 1.48)
WME method 0.75(0.47 to 1.18)
MR Egger 0.77(0.07 to 8.43)
LDL.C
MR-PRESSO 1.36(0.92 to 2.01)
IVW method 1.36(0.92 to 2.01)
WME method 1.39(0.96 to 2.00)
MR Egger 1.89(0.64 to 5.59)
TG
MR-PRESSO 0.90(0.65 to 1.26)
IVW method 0.90(0.65 to 1.26)
WME method 0.94(0.66 to 1.33)
MR Egger 0.85(0.33 to 2.19)
ApoA1
MR-PRESSO 1.04(0.77 to 1.40)
IVW method 1.04(0.77 to 1.40)
WME method 1.02(0.77 to 1.35)
MR Egger 0.54(0.21 to 1.35)
HDL.C
MR-PRESSO 1.05(0.83 to 1.35)
IVW method 1.05(0.83 to 1.35)
WME method 1.07(0.81 to 1.42)
MR Egger 1.01(0.53 to 1.94)
Method OR (95%CI) 1 2
Small vessel disease
MR-PRESSO 1.09(0.82 to 1.46)
IVW method 1.09(0.82 to 1.46)
WME method 1.15(0.82 to 1.62)
MR Egger 1.03(0.44 to 2.41)
MR-PRESSO 1.17(0.78 to 1.76)
IVW method 1.17(0.78 to 1.76)
WME method 1.28(0.80 to 2.05)
MR Egger 0.57(0.08 to 3.85)
MR-PRESSO 1.10(0.86 to 1.41)
IVW method 1.10(0.86 to 1.41)
WME method 1.05(0.76 to 1.45)
MR Egger 0.75(0.39 to 1.47)
MR-PRESSO 1.05(0.70 to 1.58)
IVW method 1.05(0.70 to 1.58)
WME method 1.14(0.78 to 1.69)
MR Egger 0.99(0.31 to 3.17)
MR-PRESSO 0.99(0.77 to 1.26)
IVW method 0.99(0.77 to 1.26)
WME method 1.01(0.74 to 1.37)
MR Egger 1.39(0.60 to 3.26)
MR-PRESSO 0.94(0.73 to 1.20)
IVW method 0.94(0.73 to 1.20)
WME method 1.01(0.75 to 1.35)
MR Egger 1.48(0.83 to 2.63)
Method OR (95%CI) 1 2
Odds ratios and 95% CI

eFigure 1. Causal relationship between main lipoprotein/lipids and CVDs in sensitivity analysis.

Red error bar: significantly positive association.

Green error bar: significantly negative association.

Blue error bar: insignificant association.

*: $P<0.05$.

†: Significant result after Bonferroni correction.

§: Results with potential horizontal pleiotropic tested by MR-Egger method.

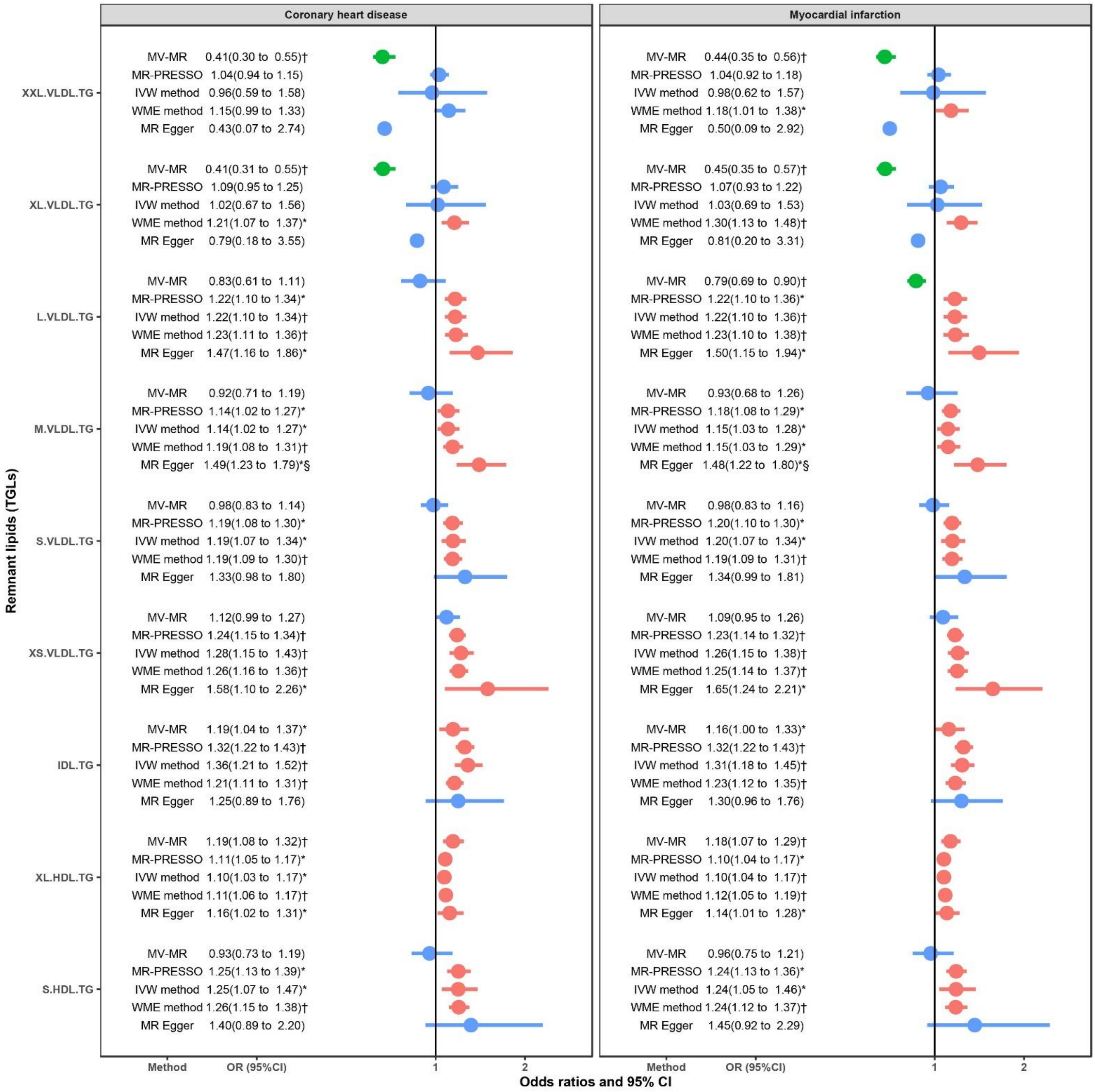

eFigure 2. Causal relationship between circulating remnant lipids and CHD and MI in sensitivity analysis.

Red error bar: significantly positive association.

Green error bar: significantly negative association.

Blue error bar: insignificant association.

*: $P<0.05$.

†: Significant result after Bonferroni correction.

§: Results with potential horizontal pleiotropic tested by MR-Egger method.

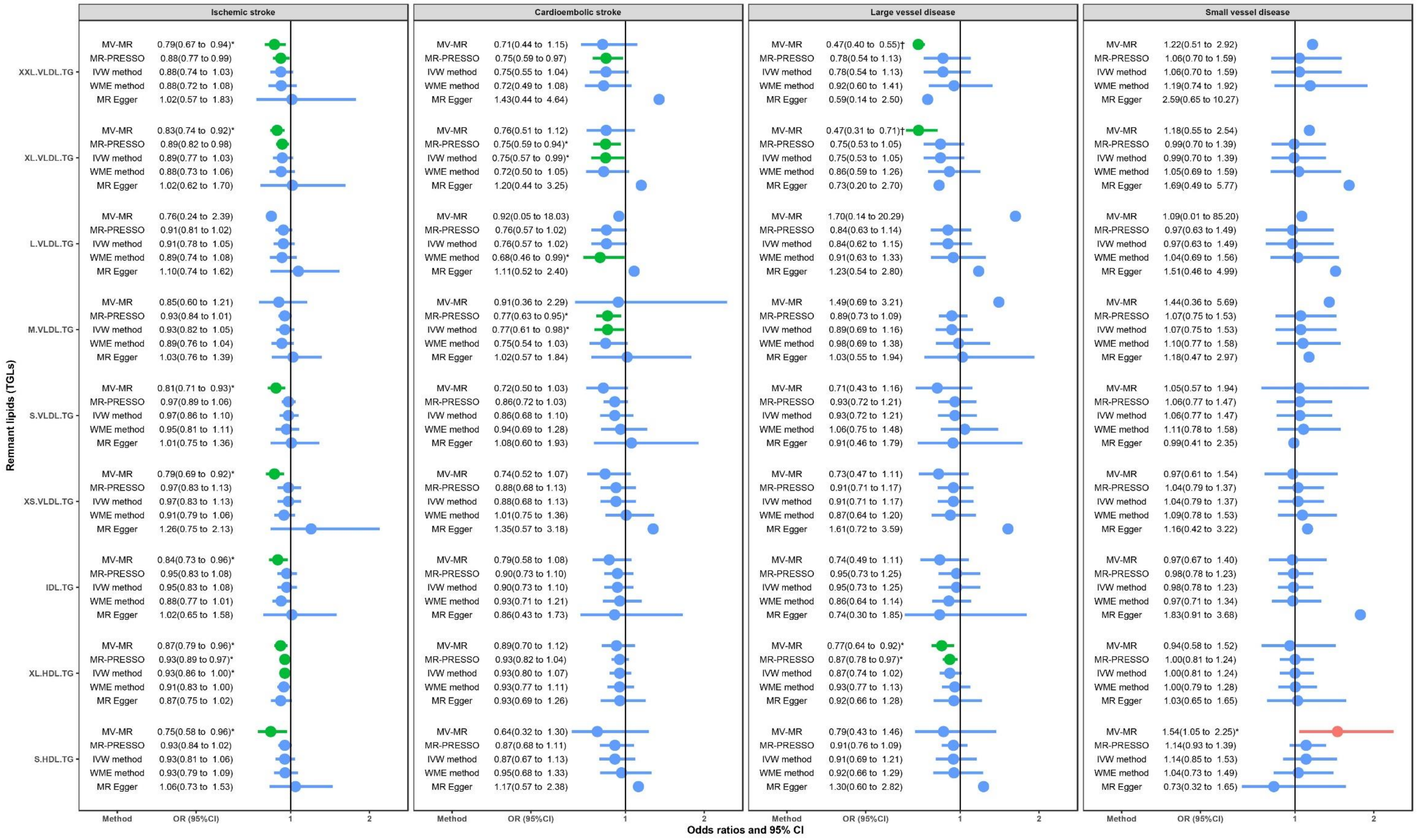
Ischemic stroke
Cardioembolic stroke
Large vessel disease
Small vessel disease
Remnant lipids (TGLs)
XXL.VLDL.TG
XL.VLDL.TG
L.VLDL.TG
M.VLDL.TG
S.VLDL.TG
XS.VLDL.TG
IDL.TG
XL.HDL.TG
S.HDL.TG
Method
OR (95%CI)
Odds ratios and 95% CI
Ischemic stroke:
MV-MR 0.79(0.67 to 0.94)*
MR-PRESSO 0.88(0.77 to 0.99)
IVW method 0.88(0.74 to 1.03)
WME method 0.88(0.72 to 1.08)
MR Egger 1.02(0.57 to 1.83)
MV-MR 0.83(0.74 to 0.92)*
MR-PRESSO 0.89(0.82 to 0.98)
IVW method 0.89(0.77 to 1.03)
WME method 0.88(0.73 to 1.06)
MR Egger 1.02(0.62 to 1.70)
MV-MR 0.76(0.24 to 2.39)
MR-PRESSO 0.91(0.81 to 1.02)
IVW method 0.91(0.78 to 1.05)
WME method 0.89(0.74 to 1.08)
MR Egger 1.10(0.74 to 1.62)
MV-MR 0.85(0.60 to 1.21)
MR-PRESSO 0.93(0.84 to 1.01)
IVW method 0.93(0.82 to 1.05)
WME method 0.89(0.76 to 1.04)
MR Egger 1.03(0.76 to 1.39)
MV-MR 0.81(0.71 to 0.93)*
MR-PRESSO 0.97(0.89 to 1.06)
IVW method 0.97(0.86 to 1.10)
WME method 0.95(0.81 to 1.11)
MR Egger 1.01(0.75 to 1.36)
MV-MR 0.79(0.69 to 0.92)*
MR-PRESSO 0.97(0.83 to 1.13)
IVW method 0.97(0.83 to 1.13)
WME method 0.91(0.79 to 1.06)
MR Egger 1.26(0.75 to 2.13)
MV-MR 0.84(0.73 to 0.96)*
MR-PRESSO 0.95(0.83 to 1.08)
IVW method 0.95(0.83 to 1.08)
WME method 0.88(0.77 to 1.01)
MR Egger 1.02(0.65 to 1.58)
MV-MR 0.87(0.79 to 0.96)*
MR-PRESSO 0.93(0.89 to 0.97)*
IVW method 0.93(0.86 to 1.00)*
WME method 0.91(0.83 to 1.00)
MR Egger 0.87(0.75 to 1.02)
MV-MR 0.75(0.58 to 0.96)*
MR-PRESSO 0.93(0.84 to 1.02)
IVW method 0.93(0.81 to 1.06)
WME method 0.93(0.79 to 1.09)
MR Egger 1.06(0.73 to 1.53)
Cardioembolic stroke:
MV-MR 0.71(0.44 to 1.15)
MR-PRESSO 0.75(0.59 to 0.97)
IVW method 0.75(0.55 to 1.04)
WME method 0.72(0.49 to 1.08)
MR Egger 1.43(0.44 to 4.64)
MV-MR 0.76(0.51 to 1.12)
MR-PRESSO 0.75(0.59 to 0.94)*
IVW method 0.75(0.57 to 0.99)*
WME method 0.72(0.50 to 1.05)
MR Egger 1.20(0.44 to 3.25)
MV-MR 0.92(0.05 to 18.03)
MR-PRESSO 0.76(0.57 to 1.02)
IVW method 0.76(0.57 to 1.02)
WME method 0.68(0.46 to 0.99)*
MR Egger 1.11(0.52 to 2.40)
MV-MR 0.91(0.36 to 2.29)
MR-PRESSO 0.77(0.63 to 0.95)*
IVW method 0.77(0.61 to 0.98)*
WME method 0.75(0.54 to 1.03)
MR Egger 1.02(0.57 to 1.84)
MV-MR 0.72(0.50 to 1.03)
MR-PRESSO 0.86(0.72 to 1.03)
IVW method 0.86(0.68 to 1.10)
WME method 0.94(0.69 to 1.28)
MR Egger 1.08(0.60 to 1.93)
MV-MR 0.74(0.52 to 1.07)
MR-PRESSO 0.88(0.68 to 1.13)
IVW method 0.88(0.68 to 1.13)
WME method 1.01(0.75 to 1.36)
MR Egger 1.35(0.57 to 3.18)
MV-MR 0.79(0.58 to 1.08)
MR-PRESSO 0.90(0.73 to 1.10)
IVW method 0.90(0.73 to 1.10)
WME method 0.93(0.71 to 1.21)
MR Egger 0.86(0.43 to 1.73)
MV-MR 0.89(0.70 to 1.12)
MR-PRESSO 0.93(0.82 to 1.04)
IVW method 0.93(0.80 to 1.07)
WME method 0.93(0.77 to 1.11)
MR Egger 0.93(0.69 to 1.26)
MV-MR 0.64(0.32 to 1.30)
MR-PRESSO 0.87(0.68 to 1.11)
IVW method 0.87(0.67 to 1.13)
WME method 0.95(0.68 to 1.33)
MR Egger 1.17(0.57 to 2.38)
Large vessel disease:
MV-MR 0.47(0.40 to 0.55)†
MR-PRESSO 0.78(0.54 to 1.13)
IVW method 0.78(0.54 to 1.13)
WME method 0.92(0.60 to 1.41)
MR Egger 0.59(0.14 to 2.50)
MV-MR 0.47(0.31 to 0.71)†
MR-PRESSO 0.75(0.53 to 1.05)
IVW method 0.75(0.53 to 1.05)
WME method 0.86(0.59 to 1.26)
MR Egger 0.73(0.20 to 2.70)
MV-MR 1.70(0.14 to 20.29)
MR-PRESSO 0.84(0.63 to 1.14)
IVW method 0.84(0.62 to 1.15)
WME method 0.91(0.63 to 1.33)
MR Egger 1.23(0.54 to 2.80)
MV-MR 1.49(0.69 to 3.21)
MR-PRESSO 0.89(0.73 to 1.09)
IVW method 0.89(0.69 to 1.16)
WME method 0.98(0.69 to 1.38)
MR Egger 1.03(0.55 to 1.94)
MV-MR 0.71(0.43 to 1.16)
MR-PRESSO 0.93(0.72 to 1.21)
IVW method 0.93(0.72 to 1.21)
WME method 1.06(0.75 to 1.48)
MR Egger 0.91(0.46 to 1.79)
MV-MR 0.73(0.47 to 1.11)
MR-PRESSO 0.91(0.71 to 1.17)
IVW method 0.91(0.71 to 1.17)
WME method 0.87(0.64 to 1.20)
MR Egger 1.61(0.72 to 3.59)
MV-MR 0.74(0.49 to 1.11)
MR-PRESSO 0.95(0.73 to 1.25)
IVW method 0.95(0.73 to 1.25)
WME method 0.86(0.64 to 1.14)
MR Egger 0.74(0.30 to 1.85)
MV-MR 0.77(0.64 to 0.92)*
MR-PRESSO 0.87(0.78 to 0.97)*
IVW method 0.87(0.74 to 1.02)
WME method 0.93(0.77 to 1.13)
MR Egger 0.92(0.66 to 1.28)
MV-MR 0.79(0.43 to 1.46)
MR-PRESSO 0.91(0.76 to 1.09)
IVW method 0.91(0.69 to 1.21)
WME method 0.92(0.66 to 1.29)
MR Egger 1.30(0.60 to 2.82)
Small vessel disease:
MV-MR 1.22(0.51 to 2.92)
MR-PRESSO 1.06(0.70 to 1.59)
IVW method 1.06(0.70 to 1.59)
WME method 1.19(0.74 to 1.92)
MR Egger 2.59(0.65 to 10.27)
MV-MR 1.18(0.55 to 2.54)
MR-PRESSO 0.99(0.70 to 1.39)
IVW method 0.99(0.70 to 1.39)
WME method 1.05(0.69 to 1.59)
MR Egger 1.69(0.49 to 5.77)
MV-MR 1.09(0.01 to 85.20)
MR-PRESSO 0.97(0.63 to 1.49)
IVW method 0.97(0.63 to 1.49)
WME method 1.04(0.69 to 1.56)
MR Egger 1.51(0.46 to 4.99)
MV-MR 1.44(0.36 to 5.69)
MR-PRESSO 1.07(0.75 to 1.53)
IVW method 1.07(0.75 to 1.53)
WME method 1.10(0.77 to 1.58)
MR Egger 1.18(0.47 to 2.97)
MV-MR 1.05(0.57 to 1.94)
MR-PRESSO 1.06(0.77 to 1.47)
IVW method 1.06(0.77 to 1.47)
WME method 1.11(0.78 to 1.58)
MR Egger 0.99(0.41 to 2.35)
MV-MR 0.97(0.61 to 1.54)
MR-PRESSO 1.04(0.79 to 1.37)
IVW method 1.04(0.79 to 1.37)
WME method 1.09(0.78 to 1.53)
MR Egger 1.16(0.42 to 3.22)
MV-MR 0.97(0.67 to 1.40)
MR-PRESSO 0.98(0.78 to 1.23)
IVW method 0.98(0.78 to 1.23)
WME method 0.97(0.71 to 1.34)
MR Egger 1.83(0.91 to 3.68)
MV-MR 0.94(0.58 to 1.52)
MR-PRESSO 1.00(0.81 to 1.24)
IVW method 1.00(0.81 to 1.24)
WME method 1.00(0.79 to 1.28)
MR Egger 1.03(0.65 to 1.65)
MV-MR 1.54(1.05 to 2.25)*
MR-PRESSO 1.14(0.93 to 1.39)
IVW method 1.14(0.85 to 1.53)
WME method 1.04(0.73 to 1.49)
MR Egger 0.73(0.32 to 1.65)
1
2

eFigure 3. Causal relationship between circulating remnant lipids and IS and subtypes in sensitivity analysis.

Red error bar: significantly positive association.

Green error bar: significantly negative association.

Blue error bar: insignificant association.

*: $P<0.05$.

†: Significant result after Bonferroni correction.

§: Results with potential horizontal pleiotropic tested by MR-Egger method.
